# DNA choreography: correlating mobility and organization of DNA across different resolutions from loops to chromosomes

**DOI:** 10.1007/s00418-024-02285-x

**Published:** 2024-05-17

**Authors:** Maruthi K. Pabba, Janis Meyer, Kerem Celikay, Lothar Schermelleh, Karl Rohr, M. Cristina Cardoso

**Affiliations:** 1 Department of Biology, Technical University of Darmstadt, Darmstadt, Germany; 2 Biomedical Computer Vision Group, BioQuant, IPMB, Heidelberg University, Heidelberg, Germany; 3 Department of Biochemistry, University of Oxford, Oxford, UK

**Keywords:** Live cell DNA labeling, Super-resolution microscopy, Widefield microscopy, Image registration, Single particle tracking, Motion analysis

## Abstract

The dynamics of DNA in the cell nucleus plays a role in cellular processes and fates but the interplay of DNA mobility with the hierarchical levels of DNA organization is still underexplored. Here, we made use of DNA replication to directly label genomic DNA in an unbiased genome-wide manner. This was followed by live-cell time-lapse microscopy of the labeled DNA combining imaging at different resolutions levels simultaneously and allowing one to trace DNA motion across organization levels within the same cells. Quantification of the labeled DNA segments at different microscopic resolution levels revealed sizes comparable to the ones reported for DNA loops using 3D super-resolution microscopy, topologically associated domains (TAD) using 3D widefield microscopy, and also entire chromosomes. By employing advanced chromatin tracking and image registration, we discovered that DNA exhibited higher mobility at the individual loop level compared to the TAD level and even less at the chromosome level. Additionally, our findings indicate that chromatin movement, regardless of the resolution, slowed down during the S phase of the cell cycle compared to the G1/G2 phases. Furthermore, we found that a fraction of DNA loops and TADs exhibited directed movement with the majority depicting constrained movement. Our data also indicated spatial mobility differences with DNA loops and TADs at the nuclear periphery and the nuclear interior exhibiting lower velocity and radius of gyration than the intermediate locations. On the basis of these insights, we propose that there is a link between DNA mobility and its organizational structure including spatial distribution, which impacts cellular processes.

**Supplementary Information:**

The online version contains supplementary material available at 10.1007/s00418-024-02285-x.

## Introduction

The dynamic yet functionally stable organization of cellular processes is a crucial feature of biological systems. Their dynamic nature is indispensable as it enables biological systems to respond to external stimuli and survive. The eukaryotic nucleus is a sophisticated subcellular organelle in which the intricate processes of DNA and RNA metabolism take place. The nucleus serves as the spatial and temporal epicenter for orchestrating cellular processes such as DNA replication, repair, and transcription. DNA is spatiotemporally organized within the cell nucleus in the form of chromatin. This architectural arrangement undergoes regulation at various tiers of genomic organization. Multiple studies have substantiated the dynamic nature of chromatin, revealing that it undergoes alterations in composition and architecture in response to diverse cellular processes.

Notably, chromatin, the blend of DNA and proteins that constitutes chromosomes, is far from a static entity but rather exhibits motion within the cell nucleus. This motion dynamically interplays with (patho)physiological cellular processes such as DNA transcription (Nozaki et al. 2017; Tortora et al. 2020; Miron et al. 2020; Laghmach et al. 2021; Mach et al. 2022), DNA replication (Heun et al. 2001; Levi et al. 2005; Levi and Gratton 2008; Zidovska et al. 2013; Tortora et al. 2020; Gabriele et al. 2022; Pabba et al. 2023), DNA repair (Miné-Hattab et al. 2017), and cellular senescence (Shaban and Gasser 2023) and diseases (Li et al. 2022). For instance, during transcriptional activation or repression, specific genetic loci undergo conformational changes that involve alterations in chromatin structure and motion (Germier et al. 2017; Shaban et al. 2018; Shaban and Suter 2022). Furthermore, the dynamic nature of chromatin is indispensable for the successful execution of DNA replication and repair processes by facilitating the access to the DNA segments undergoing replication or repair (Zidovska et al. 2013; Miné‑Hattab et al. 2017; Ma et al. 2019; Ochs et al. 2019; Pabba et al. 2023). Previous studies have shown that chromatin motion plays an important role in maintaining interactions between topologically associated domains (TADs) for an average of 10 min to facilitate gene expression and this was shown to be enabled by cohesin and CTCF (Mach et al. 2022). Fluctuations in chromatin dynamics can have profound effects on gene expression and contribute to the onset or progression of diseases, such as cancer (Guasconi and Ait‑Si-Ali 2004; Gurova 2022; Li et al. 2022). Hence, understanding the mechanisms that underlie chromatin architecture and dynamics is of paramount importance, which requires strategies to label and visualize DNA in an unbiased genome-wide manner in living cells.

In eukaryotes, DNA/chromatin is folded hierarchically. At the megabase scale, gene-rich transcriptionally active regions tend to interact among them, while gene-poor heterochromatic regions also interact more frequently, giving rise to A and B compartments, respectively, in contact frequency maps from Hi-C experiments. These interactions between Mb-large chromatin regions remain mostly unchanged between different cell types (McArthur and Capra 2021; Harris et al. 2023). At the sub-megabase scale, chromatin domains with high/malleable interaction frequencies and relatively isolated from neighboring regions form TADs. TADs are large self-interacting genomic regions that compartmentalize chromosomes into discrete domains with distinct functional characteristics and are characterized by intradomain chromatin contact frequency (Sexton et al. 2012; Dixon et al. 2012, 2016). TAD boundaries are typically associated with a signature set of proteins, including CCCTC-binding factor (CTCF), structural maintenance of chromosomes (SMC) complex such as cohesin and condensin, and RNA polymerase II (Dixon et al. 2012; Phillips‑Cremins et al. 2013; Van Bortle et al. 2014; Rao et al. 2014; Jung et al. 2017). TADs form as a result of loop extrusion, wherein the DNA is translocated through the cohesin or SMC ring complex forming loop domains or nano-foci (Rao et al. 2014; de Wit et al. 2015; Sanborn et al. 2015; Fudenberg et al. 2016, 2017; Knoch et al. 2016; Natale et al. 2017; Ganji et al. 2018; Grubert et al. 2020; Cremer et al. 2020; Mach et al. 2022). Previous studies using Hi-C have shown that the TAD domains are significantly variable in sizes and numbers and that it is challenging to define the average TAD sizes across cell types (Dixon et al. 2016; Zufferey et al. 2018). Various conformation capture techniques have revealed that the TAD domains range from few hundred kilobase pairs (kbp) to megabase pairs (Mbp) in sizes (Szabo et al. 2019; Long et al. 2022). Earlier studies have estimated chromatin loop sizes accurately in HeLa cells using DNA halo measurements between 5 and 200 kbp (Jackson et al. 1990). Recent advances have narrowed down the average chromatin loop sizes to 185 kbp (median) (Rao et al. 2014; Mamberti and Cardoso 2020).

Recent advancements in microscopy techniques, including fluorescent in situ hybridization, super-resolution microscopy, and polymer simulations, have facilitated the visualization of chromatin loop domains within cells (Trzaskoma et al. 2020; Hao et al. 2021; Park et al. 2021; Maslova and Krasikova 2021; Brandstetter et al. 2022; Parteka‑Tojek et al. 2022; Sabaté et al. 2023). However, these studies lack a direct comparison of DNA mobility at the level of TADs and chromatin loops and entire chromosomes, which would allow us a better understanding of the nature of chromatin dynamics. 3D structured illumination microscopy (SIM) is a technique that enables computational reconstruction of super-resolved images from a series of widefield images that are illuminated with shifted periodic stripe pattern with a frequency close to the resolution limit (Gustafsson 2008). Average projecting the raw structured illumination data generate conventional (pseudo)widefield images, which has been widely employed to obtain corresponding images of the same samples at different resolution level (Neil et al. 1997; Albiez et al. 2006; Baddeley et al. 2010; Chagin et al. 2016; Brandstetter et al. 2022). However, there is a notable absence of correlative microscopy comparisons pertaining to DNA mobility at the different levels of organization. Correlative microscopy, a powerful technique that combines different imaging modalities of the same sample, allows one to visualize chromatin in real time at both higher and lower resolutions. In this study, we address the question of whether the motion of DNA/chromatin is correlated across multiple resolutions from individual DNA loops to (sub)megabase chromosome domains to entire chromosomes. In this context, we directly labeled DNA in living cells in an unbiased manner encompassing the whole genome and quantified its hierarchical organization levels. We used single particle tracking combined with image registration and motion analysis to characterize and compare the mobility of DNA loops, (sub)megabase chromosome domains, and entire chromosomes.

## Methods

### Cells

All cells used were tested and negative for mycoplasma. Human cervical cancer cell line HeLa Kyoto (Erfle et al. 2007) and HeLa K cells expressing GFP-PCNA (Chagin et al. 2016; Pabba et al. 2023) fusion protein were used to monitor cell cycle progression. All cells were maintained in Dulbecco’s modified Eagle medium (DMEM) (Cat. No. 41965039, Thermo Fisher Scientific, USA) supplemented with 10% fetal calf serum (Cat. No. FBS 11A, Capricorn Scientific GmbH, Germany), 1× l-glutamine (Cat. No. 392-0441, VWR, Germany), 1× sodium pyruvate (Cat. No. 10703688, Th.Geyer GmbH, Germany), and 1× gentamicin (Cat. No. G1397-100 ml, Th.Geyer GmbH, Germany) in a humidified atmosphere with 5% CO_2_ at 37 °C. Previously published experiments confirmed that the transgenic gene product colocalized with the endogenous protein and was present at sites of active replication (Chagin et al. 2016; Pabba et al. 2023). The culture medium was changed every day and cells were split every 2 days. Cell line characteristics are summarized in Supplementary Table 1.

### Replication labeling and live cell imaging

HeLa K GFP-PCNA cells were seeded in low density onto Ibidi μ-dish chambers (Cat. No. 80826, Ibidi, Germany). To fluorescently mark the active synthesis of nascent DNA at the replication sites, the cells were seeded on glass and were scratch loaded using a needle with 100 µM of ATTO-590-dUTP or Cy3-dUTP (Schermelleh et al. 2001; Sadoni et al. 2004) (Supplementary Table 2). After scratch loading cells were allowed to recover overnight and were imaged the next day. All imaging was performed at 37 °C (unless specified) with a humidified atmosphere and 5% CO_2_ (Supplementary Table 4).

### Chromatin compaction analysis

**Fig. 1:**
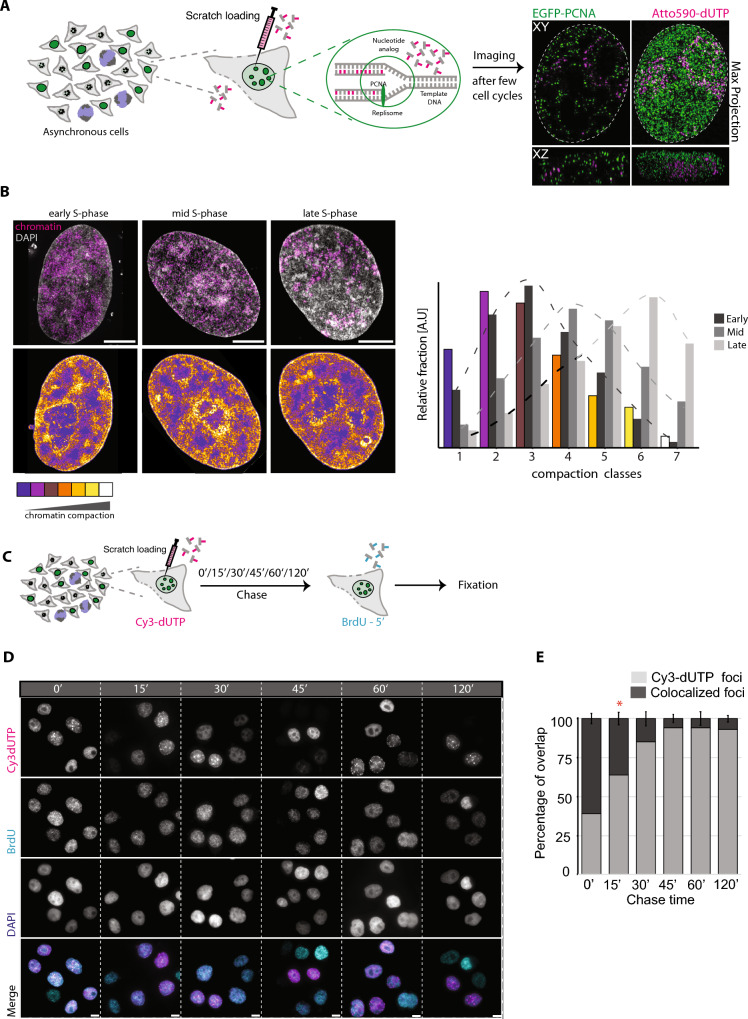
Genome-wide DNA labeling and estimation of nucleotide pulse duration. **A** Illustration of the labeling scheme. Fluorescently labeled nucleotides are introduced in an asynchronous population of HeLa K GFP-PCNA cells (Supplementary Table 1) using scratch loading before imaging in subsequent cell cycle stages (Replication labeling and live cell imaging, Supplementary Table 4). On the right, a single Z slice and maximum Z projection of a representative cell with GFP-PCNA (green) and ATTO-590 dUTP (magenta) is shown at SIM resolution (Supplementary Table 2). **B** Representative images of HeLa K cells labeled with nucleotides (magenta) and DAPI (gray). The replication labeling pattern was used to determine the S phase stage where the chromatin was labeled in the previous cell cycle. We then performed chromatin compaction class analysis using DAPI intensity (Methods) of 3D-SIM imaged fixed cells and mapped the distribution of chromatin label within the compaction class and plotted it as a bar plot. **C** The labeling scheme to determine the DNA labeling duration using scratch loading, we first labeled cells with Cy3-dUTP (magenta) using scratch loading followed by different chase times (0′/15′/30′/45′/60′/120′), which was then followed by a second nucleoside pulse (BrdU—40 µM) for 5 min to label cells. The cells were then fixed after a few hours and BrdU detection was performed (Methods, Supplementary Table 3). **D** The cells were then imaged using a high-throughput widefield microscope (Supplementary Table 4) and representative images of all samples are shown (Cy3-dUTP—magenta, BrdU—cyan, DAPI—blue). **E** We performed colocalization analysis of both labels to determine the overlap percentage over time (Supplementary Fig. 2), which was then plotted as bar plots with error bars. Scale 5 µm

HeLa K GFP-PCNA cells were labeled with nucleotides using scratch loading on coverslips. Next day the cells were fixed using 3.7% formaldehyde for 10 min. Then, 1 mg/ml 4′,6-diamidino-2-phenylindole (DAPI) (Cat. No. 6335.1, Carl Roth, Germany) was used to stain the DNA for 10 min. The cells were then imaged in 3D (Z stacks) using super-resolution 3D-SIM to image DNA and nucleotides. The reconstructed and thresholded 16-bit super-resolved images were analyzed using “Nucim” library available on platform “R” to subdivide individual nuclei into chromatin compaction classes and mapping signals from other channels to individual compaction classes (Smeets et al. 2014). First, the DAPI channel was segmented and used to mask the region of interest. Individual voxels within a single nucleus were assigned to a certain compaction class based on the probability of this voxel belonging to the same class computed from a hidden Markov random field (HMRF) stochastic model, classifying the nuclei into seven different compaction classes, where the first class represents interchromatin (IC), as opposed to the chromatin compartment of chromatin domain clusters (CDCs). Classes two, and three represent less compacted perichromatin located towards the surface of CDCs wich together with IC form the active nuclear compartment (ANC), whereas classes four to seven located towards the interior of chromatin domain clusters (CDC) represent the more compacted inactive nuclear compartment (INC) (Fig. 1B). Spots from other channels were further mapped into these subclasses based on a combined threshold and intensity method where first the spots were segmented with the threshold method followed by an intensity-weighted calculation of the relative fraction, leading to more intense signals having a larger impact and low-intensity signals having less impact.

### Double pulse analysis to determine the labeling duration

HeLa Kyoto cells were seeded on coverslips (glass) at 50% confluency the day before. Next day the cells were labeled with 100 µM Cy3-dUTP using scratch loading to label the active replication sites (Supplementary Table 2). The scratch-loaded cells (coverslips) were then submerged 10% DMEM media (Cells) with 40 µM BrdU nucleoside added for 5 min at different chase times (0′, 15′, 30′, 45′, 60′, 120′). The coverslips with cells were then fixed in 3.7% formaldehyde for 10 min and washed twice with 1× PBS. The cells were then treated with 0.7% TritonX for 20 min and washed three times with 1× PBS to remove excess TritonX. BrdU detection was performed using (2% BSA in 1× PBS, 1× DNase buffer, DNase 2000 U/ml, rabbit anti-BrdU (1:500) for 1 h (Supplementary Table 3). The excess antibody was washed using 1× PBS–0.05% Tween20. The BrdU was then detected using anti-rabbit A488 (1:500) for 1 h (Supplementary Table 3). The excess secondary antibody was washed using 1× PBS–0.05% Tween20. Then, 1 mg/ml DAPI (Cat. No. 6335.1, Carl Roth, Germany) in H_2_O was used to stain the DNA for 10 min. Excess DAPI was washed away using H_2_O. The slides were mounted using Vectashield  mounting medium (Cat. No. VEC-H-1400, Vector Laboratories, USA). The cells were then imaged using Nikon TiE2 microscope (Supplementary Table 4) and analyzed using CellProfiler to determine the colocalization percentage of Cy3-dUTP and BrdU for different chase times (Supplementary Table 5, Supplementary Fig. 2, Fig. 1B).

### Cell cycle profiling using flow cytometry

HeLa K cells were seeded at 30% confluency in p100 cell culture dishes (Cat. No. 83.3902, Sarstedt, Germany). After 24 h, the culture medium was removed and the cells were washed with 1× PBS/EDTA followed by 5 min trypsin (Cat. No. TRY-3B, Capricorn Scientific GmbH, Germany) treatment. Once the cells were dissociated, they were collected in a 15-ml tube and pelleted down at 500 r.c.f. (relative centrifugal force) for 5 min. The pellet was resuspended in a small volume of 1× PBS until cells were dissociated completely. Then, 100% ice-cold ethanol was added dropwise to a final concentration of 70%. The cells were fixed in 70% ethanol for at least 4 h. Cells were then pelleted in ethanol at 500 r.c.f. for 5 min. The ethanol was removed, and cells were resuspended in DNA staining solution (1× PBS with 100 µg/mL RNase A, 50 µg/ml propidium iodide (PI), and 0.1% Triton X-100) for overnight staining at 4 °C. Next day the cells were again washed with 1× PBS before proceeding with FACS sorter. The cells were then resuspended in fresh ice cold 1× PBS at 0.5 million cells/mL. A Bio Rad cell sorter (Supplementary Table 4) was used to gate single cells, followed by removal of doublets from the population. The FL-2 laser line was used to detect the PI, and a histogram was plotted of the number of cells versus PI intensity. The data was collected for five million cells (Supplementary Fig. 3). The data from the cell sorter was analyzed using FCS express (Denovo software by Dotmatics) to obtain the cell cycle profile (Supplementary Table 5).

### Metaphase spreads

HeLa K cells were passaged into 4 p100 (Cat. No. 83.3902, Sarstedt, Germany) cell culture dishes at 60% confluency. The next day Colcemid (Cat. No. COL-H, Capricorn Scientific GmbH, Germany) was added to the cell culture media to a final concentration of 0.1 µg/ml for 4 h. The medium was aspirated, and the cells were then washed with 1× PBS to remove excess Colcemid. The cells were then trypsinized (Cat. No. TRY-3B, Capricorn Scientific GmbH, Germany) for 5 min and dissociated cells were collected into 50-ml tubes and centrifuged at 500 r.c.f. for 5 min. The supernatant was aspirated and was followed by dropwise addition of 0.75 mM 37 °C KCl in H_2_O and incubated at room temperature (RT) for 20 min. The cells were then centrifuged for 5 min at 500 r.c.f. The supernatant was then aspirated followed by dropwise addition of ice cold 3:1 methanol/acetic acid fixative solution. The fixation was repeated two times. Finally, the pellet was resuspended in 2.5 ml cold fixative and stored at −20 °C overnight. The next day, the cells were again pelleted and resuspended in 5 ml of ice-cold fixative. The metaphases were then added dropwise on a wet microscope slide from a height of 10–20 cm using a 1-ml pipette before drying at RT overnight. The next day, the slides with metaphases were rehydrated for 5 min in H_2_O. and stained with 1 mg/ml DAPI (Cat. No. 6335.1, Carl Roth, Germany) in for 10 min. Excess DAPI was washed away using H_2_O. The slides were mounted using Vectashield (Cat. No. VEC-H-1400, Vector Laboratories, USA) and imaged using the Nikon TiE2 microscope (Supplementary Table 4).

### Microscopy

Imaging was performed within a few days after the labeling of cells using a DeltaVision OMX V3 Blaze system (Applied Precision), equipped with a 60×/1.42 NA Plan Apo oil objective and Olympus and pco.edge 4.2 sCMOS cameras (PCO, Kelheim, Germany) for high-speed stack acquisition. 3D-SIM image stacks were acquired with a z-distance of 125 nm and with 15 raw images per plane (five phases, three angles). For time-lapse acquisitions, typically stacks of 7 consecutive z-sections covering ~1 µm height were recorded with 10 s intervals. 3D-pseudo-widefield (WF) images were generated by average projecting the raw SI images per plane and have a lateral (x–y) resolution of approximately 240 nm and an axial (z) resolution of ~600 nm. The raw data were computationally reconstructed with the SoftWoRx 6.1 software (GE Healthcare) using channel-specific optical transfer functions (OTFs) and a Wiener filter setting of 0.0030 to obtain a super-resolution 3D-SIM image stack with a lateral (x–y) resolution of ~120 nm and an axial (z) resolution of ~300 nm. Post-reconstruction quality control, thresholding & 16-bit conversion and channel alignment was performed as described elsewhere (Ochs et al. 2019; Miron et al. 2020). In some cases, WF dataset were subjected to an additional iterative 3D deconvolution with SoftWoRx using the default settings. 

ATTO-590 and GFP were excited sequentially using 592 nm or 488 nm laser lines to minimize cross talk. Imaging was performed at 37 °C with a humidified atmosphere using an environmental chamber unless otherwise mentioned (Supplementary Table 4).

The standard protocol for examining chromatin mobility in ATTO-590-labeled nuclei proceeded in the following manner: First, a reference image of GFP-PCNA and ATTO-590-dUTP, was collected from a single focal plane corresponding to the middle of the nucleus. This image demarcated the nuclear boundary, provided cell cycle information, and, in the case of S phase cells, allowed us to correlate the positions of ATTO-590-dUTP foci with sites of DNA replication. Second, while maintaining the same central focal plane, a time series (frame interval of 10 s, 12 frames) and 3D volume with fewer stacks were captured to minimize photo toxicity. To perform the imaging at different temperatures, the labeled cells were imaged in both channels at 37 °C and RT.

The GFP-PCNA was used to distinguish the cell cycle stages. In some cases, only one channel ATTO-590-dUTP was imaged to minimize bleaching. The imaging was performed over multiple experiments to have reproducibility and sufficient replicates.

### DNA combing

HeLa K cells were transfected with digoxigenin (DIG)-11 dUTP using the Neon transfection system (Cat. No. MPK5000, Invitrogen, USA) (Supplementary Fig. 6). Three hours after transfection the cells were trypsinized and dissolved as 50,000 cells per 50 µl PBS. The cells were then embedded in equal volume of 2% sieve GP low melt agarose (Cat. No. 850090, Biozym scientific GmbH) and the plugs were cooled for 30 min at 4 ºC . The plugs were then incubated in lysis buffer (1% sarcosyl (Cat. No. 8147150500, Merck millipore GmbH, Germany) 125 mM EDTA pH 9.5) with 1 mg/ml proteinase K (Cat. No. P2308-500 mg, Merck millipore GmbH, Germany) overnight at 42 °C. The agarose plugs were then transferred to 1× TE 100 mM NaCl and washed for 3 days by changing the buffer periodically. The agarose plugs were then melted in 2 ml 50 mM MES (Cat. No. 475893-100 GM, EMD millipore, MA, USA) 100 mM NaCl pH 6 at 75 ºC. The DNA was combed using the FiberComb (Cat. No. MCS-001, Genomic vision, France) on silanized coverslips (Cat. No. COV-002-RUO, Genomic vision, France). We then let the coverslips air dry for a few hours and fixed them using 3:1 methanol acetic acid (Cat. No. 6727.2 and 6755.2, Carl Roth GmbH) for 10 min. We then proceeded with immunostaining to detect DIG nucleotides using rabbit primary anti-DIG (1:500) and secondary anti-rabbit IgG conjugated with Cy3 (1:500) (Supplementary Table 3). The DNA was then detected using 1 µM YOYO-1 Iodide (Cat. No. Y3601, Thermo Fisher Scientific, USA) in TE and mounted in Vectashield (Cat. No. H-1000-10, Vector Laboratories, USA). The slides were then imaged using a Nikon TiE2 spinning disk (Supplementary Table 4).

To calculate the stretching factor of combed DNA, 50 µl (500 µg/ml) of the bacteriophage lambda DNA (cI857ind 1 Sam 7) (Cat. No N3011S, New England Biolabs, USA) was dissolved in 3 ml of MES buffer (50 mM MES (Cat. No. 475893-100 GM, EMD millipore, MA, USA) 100 mM NaCl pH 6). The DNA solution was homogenized for 2 days at 4 °C. The phage DNA was then combed as described above and stained with 1 µM YOYO-1 Iodide (Cat. No. Y3601, Thermo Fisher Scientific, USA) in TE and mounted in Vectashield (Vector Laboratories, USA, Cat. No. H-1000-10).

### DNA quantification of labeled chromatin

HeLa K GFP-PCNA cells were labeled with Cy3-dUTP using scratch loading and fixed with 3.7% paraformaldehyde in 1× PBS for 10 min and washed twice with 1× PBS. Then, 1 mg/ml DAPI (Cat. No. 6335.1, Carl Roth, Germany) in H_2_O was used to stain the DNA for 10 min. Excess DAPI was washed away using H_2_O. The slides were mounted using Vectashield mounting medium (Cat. No. VEC-H-1400, Vector Laboratories, USA). The full Z stacks of fixed cells were imaged as described above on a DeltaVision OMX microscope to detect DNA and labeled chromatin (Supplementary Table 4). The relative genome size (cell cycle correction factor,* C*) of HeLa K GFP-PCNA cells in different cell cycle stages was determined using the cell sorter by measuring the DNA (PI) intensity over cell cycle (Cell cycle profiling using flow cytometry). DNA quantification of the labeled foci was done using image analysis. The images were pre-processed using Fiji to remove the noise from the images (Supplementary Fig. 5). For segmentation of replication foci, the protocol used was originally described in Chagin et al. (2015). In brief, the images were converted into 16-bit images. Replication foci were thresholded in ImageJ with the auto-threshold using the triangle method on the stack histogram, the thresholded image was combined with the original image via Image calculator (method: min) creating a new image that contains the intensities of the original image but only in the thresholded areas. This new image and the corresponding other channels were then imported into the image analysis software Perkin Elmer Volocity 6.3 and converted into volumes. The pixel dimensions of the images were set to the specifications of the 3D-SIM and WF images. Find objects (“nucleus”) using the DAPI channel, method “Intensity” (set manually to the optimal value), use fill holes in object/dilate/erode until the object optimally fits the nucleus, exclude objects by size < 500 μm^3^. Find objects using the label channel, method “Intensity” (lower limit, 1; upper limit, maximum), separate touching objects, exclude “foci '' not touching the “nucleus”. The DNA content of the foci was determined via the DAPI sum intensities (Supplementary Fig. 5). The relative DNA ratio of labeled chromatin foci and total nuclear DNA (DAPI) was obtained. This ratio was then multiplied by the cell cycle correction factor (*C*, determined by PCNA pattern) and the genome size of HeLa K GFP-PCNA (Chagin et al. 2016). The amounts of DNA (in kbp) of individual foci were then plotted as a histogram for both SIM and WF datasets (Fig. 3).

### Registration of live-cell image data

To cope with cell movement and deformations when determining the motility of 3D chromatin structures in live-cell image data, affine image registration was performed using the deep learning method in Celikay et al. (2022) followed by non-rigid registration using the method in Balakrishnan et al. (2019). The registration transformation was computed based on the PCNA channel of the WF images and subsequently applied to the chromatin channel of the 3D-SIM and WF images. Smooth non-rigid transformations were obtained by downscaling the original input images followed by upscaling the computed transformation to the original image size. All frames of a video were registered to the first frame.

### Tracking of 3D chromatin structures in live-cell image data

Three-dimensional chromatin structures were tracked in 3D (x, y, z) within single cell nuclei in 3D live-cell fluorescence microscopy images to determine the motility. A probabilistic particle tracking method was used to determine the movement of multiple fluorescently labeled 3D chromatin structures (Ritter et al. 2021). The method is based on Bayesian filtering and multi-sensor data fusion and combines Kalman filtering with particle filtering. Multiple measurements are integrated by separate sensor models and sequential multi-sensor data fusion, which allows including different uncertainties. Elliptical sampling is employed to obtain detection-based and prediction-based measurements (Godinez and Rohr 2015), and for correspondence finding, motion information based on displacements from past time points is exploited. The spot-enhancing filter (SEF) (Sage et al. 2005) is used for the detection of 3D chromatin structures. First, a Laplacian of Gaussian (LoG) filter is applied to the images of an image sequence. Then, thresholding of the filtered images is performed using a threshold determined by the mean of the filtered absolute intensity values plus a factor times the standard deviation. The same factor is used for all images of an image sequence. Finally, local maxima are computed yielding object detections (Supplementary Fig. 8). Note that as a result of lower resolution in the z-direction compared to the x–y plane of the 3D data, the localization and tracking accuracy in the z-direction is generally lower. Chromosome territories were tracked in 2D (x, y) using a maximum intensity projection of the WF images since no movement in the z-direction was observed. We used nearest neighbor association after region segmentation employing a difference of Gaussian (DoG) filter followed by intensity thresholding.

### Motion analysis of 3D chromatin structures

The motility of 3D chromatin structures was analyzed for 3D-SIM and corresponding WF image data using a mean squared displacement (MSD) analysis (Saxton 1997). We determined the MSD as a function of the time interval Δ*t* for each computed trajectory (Supplementary Fig. 8) and averaged the MSD curves for all trajectories. To improve the accuracy of the motility analysis, we considered only trajectories with a minimum time duration of 40 s (corresponding to four time steps). We fitted both the diffusion model and the anomalous diffusion model to the calculated MSD values to obtain the diffusion coefficient *D* [μm^2 ^s^−1^] and the anomalous diffusion coefficient α (motion type parameter), respectively. The motion of chromatin structures was further characterized by different 3D motion properties: radius of gyration (Saxton 1993), mean velocity over time, distance start–end (distance between the first and last position of a trajectory) (Beltman et al. 2009), and track straightness (Beltman et al. 2009) (distance between the first and last position divided by the sum of the distances between all consecutive points of a trajectory).

To analyze the motility of the 3D chromatin structures with respect to the location within the cell nucleus (location-based motion analysis), we divided each cell nucleus into seven shells of equal volume. For this, we performed segmentation of the cell nucleus using Otsu thresholding (Otsu 1979) and determined the convex hull from the contour of the segmentation mask using the Jarvis–March algorithm (Jarvis 1973). The resulting polygon was then scaled relative to its center-of-mass to generate seven polygons representing shells of equal volume. Each trajectory was assigned to the shell which contains the majority of its points.

Motion subpopulations were determined by classifying the trajectories based on the anomalous diffusion coefficient α using k-means clustering (MacQueen 1967). We initialized the clustering algorithm using the k-means++ method (Arthur and Vassilvitskii 2007), where initial population centroids are selected from all data points (α values) to be well spread out. The centroids are defined as the mean of the data points of a subpopulation. Their initial values are iteratively refined in the k-means algorithm by changing the assignment of the data points to their closest centroid and recomputing the centroids as the new mean of the assigned data points. The classification was performed separately for 3D-SIM and WF images. The spatial distribution of the motion subpopulations inside the cell nucleus was computed using the same shells of equal volume as for location-based motion analysis.

To test for significance of the difference of the motion properties between two sets of trajectories, we performed two-sided Wilcoxon rank-sum tests.

## Results and discussion

### DNA labeling genome-wide

Earlier studies have employed various labeling techniques to mark chromatin, such as the insertion of ectopic DNA sequence arrays into chromosomes alongside ectopic expression of bacterial proteins that bind to these arrays, thereby labeling them. Additionally, specific genomic sequences have been visualized using techniques based on TALE or CRISPR/Cas9 (Ma et al. 2016, 2019) or the inclusion of ectopic sites such as LacO or TALE sites (Robinett et al. 1996; Vazquez et al. 2001; Dimitrova et al. 2008; Mach et al. 2022), where the TAL effector protein or catalytically inactive Cas9 protein binds to and labels specific loci. These methodologies have facilitated the study of chromatin dynamics across different processes, circumventing technical and analytical challenges associated with larger, clustered chromatin labels. However, it is important to note that these ectopic manipulations may introduce artifacts that do not accurately represent the true nature of chromatin.

To address this concern, we adopted the scratch loading technique, which involves the introduction of labeled deoxyribonucleotides into cells to label DNA directly (Cells) (Schermelleh et al. 2001; Sadoni et al. 2004).

This method has proven effective for the rapid labeling of DNA in cells without altering their native chromatin state. The process of genome duplication, known as DNA replication, occurs during the synthesis (S) phase, where the chromosomes are duplicated. By directly labeling chromatin/DNA in replicating S phase cells, we can label any chromatin type (euchromatin, facultative heterochromatin, constitutive heterochromatin) as well as DNA repeat elements like LINEs and SINEs as well as tandem repeats, which are overlooked in most studies. As the human genome is GC poor, we selected dUTP for labeling. This is also advantageous over dCTP as the latter could interfere with cytosine modifications. Through the use of directly labeled deoxyribonucleotides and scratch loading, we can label the DNA genome-wide (Fig. 1A, B) and examine chromatin dynamics in its native state over several cell cycles. As a larger portion of genomic DNA is packaged into heterochromatin, this would be reflected also by this labeling method.

In our study, we utilized an asynchronous population of human HeLa K GFP-PCNA cells (Chagin et al. 2016; Pabba et al. 2023) containing fluorescently tagged proliferating cell nuclear antigen (PCNA). We labeled these cells using scratch loading with 100 µM ATTO590-dUTP (Methods, Supplementary Tables 1, 2). PCNA is a component of the DNA replication machinery and serves as a marker for cell cycle progression (Fig. 1A; Prelich et al. 1987; Leonhardt et al. 2000; Easwaran et al. 2005; Moldovan et al. 2007; Chagin et al. 2016; Pabba et al. 2023). The labeled cells were allowed to divide through multiple cell cycles, thereby distributing the label to daughter cells and increasing the population of cells with labeled chromatin. These labeled cells were then subjected to two-color 3D live-cell time-lapse correlative microscopy of an approximately 1-µm-high central subvolume acquired with 10-s intervals, where we obtained high-resolution 3D-SIM images along with the corresponding lower-resolution pseudo-WF images, generated from the same raw data. Additionally, we acquired multiple full Z stacks (volumetric imaging) per cell (Fig. 1A, Supplementary Fig. 1, Video 1, Microscopy). To cover cell populations in different cell cycle stages, we utilized GFP-PCNA patterns to select cells in specific stages for live cell microscopy. The representative images of a live mid S phase (GFP-PCNA focal pattern, green) cell with labeled chromatin (nucleotides, magenta) in pseudo-WF, deconvolved widefield (Deconv WF), 3D-SIM along with the zoom section are shown in Supplementary Fig. 1.

Given that the measurement of chromatin motion of labeled chromatin domains may depend on the size of the object (labeled DNA), it is imperative to assess the labeling duration and the size of the labeled DNA domains. This evaluation enables correlating between chromatin domain sizes and their diffusion rates. As the scratch loading method involves a short-term permeabilization and uptake of fluorescent dUTPs from the medium, the pulse duration cannot be controlled. Therefore, we conducted a pulse-chase-pulse experiment to determine the labeling/pulse duration. Briefly, we employed an asynchronous population of HeLa K GFP-PCNA cells and performed scratch loading with labeled nucleotides (1st pulse) to label replicating DNA (Methods, Supplementary Tables 1, 2). We then performed a chased of different durations (0′, 15′, 30′, 45′, 60′, 120′) and labeled the cells with a second pulse of 40 µM BrdU (5-bromo-2′-deoxyuridine, a cell-permeable nucleoside), followed by fixation and detection of BrdU using antibodies (Fig. 1B, C; Double pulse analysis to determine the labeling duration; Supplementary Tables 2, 3). We then performed high-throughput imaging, which allowed us to obtain a larger cell population for quantification (Fig. 1C, Supplementary Table 4). We segmented the nuclei marked by DAPI staining and DNA foci labeled with fluorescently labeled nucleotides (1st pulse, magenta, scratch loading) and BrdU (2nd pulse, cyan, nucleoside) channels and performed colocalization analysis between the pulses to calculate the percentage of labeled nucleotides foci overlapping with BrdU foci (Fig. 1C, Supplementary Fig. 2, Supplementary Tables 5, 6). The lack of colocalization is used as a proxy for the duration of the first pulse. Our findings indicate that the nucleotide pulse during scratch loading is primarily incorporated into the genome within the initial 15 min, providing insight into the labeling duration of DNA/chromatin (Fig. 1D).

### Quantification of the size of labeled chromatin domains

As the chromatin diffusion/dynamic rates may be influenced by the chromatin domain sizes, we wanted to investigate the DNA domain sizes that were labeled within the 15 min of labeling duration after scratch loading. Hence, we performed microscopic imaging at different modes of resolution (WF, WF deconvolution, and super-resolution 3D-SIM) to quantify the corresponding labeled DNA domain sizes.

In previous studies, we have quantified the genome size (GS) of HeLa K cells to be GS = 9.682 ± 0.002 Gbp (Chagin et al. 2016). During S phase the genome is duplicated, whereby the total DNA is doubled from G1 to G2 progression before cell division in mitosis. Therefore, to precisely measure the relative DNA amount during S phase we utilized flow cytometry to determine the relative DNA amounts during the cell cycle progression (Supplementary Fig. 3). Briefly, we used ice-cold methanol-fixed HeLa K GFP-PCNA cells labeled with the DNA/RNA dye PI in combination with RNase A (to remove the RNA detection) and performed flow cytometry to detect the total amount of DNA. We then plotted the total DNA (PI) intensity on the x-axis and the number of cells on the y-axis (Methods, Supplementary Fig. 3). The DNA intensity profile over the cell cycle was fitted with the aneuploid profile of HeLa K cells (Metaphase spreads, Supplementary Fig. 4), to obtain the relative DNA amount present in early, mid, late S phase cell cycle stages. Using these data we obtained the cell cycle correction factor (*C*; G1—1, eS—1.06, mS—1.27, ls—1.71, G2—1.98) for all cell cycle stages (Supplementary Fig. 3).

**Fig. 2:**
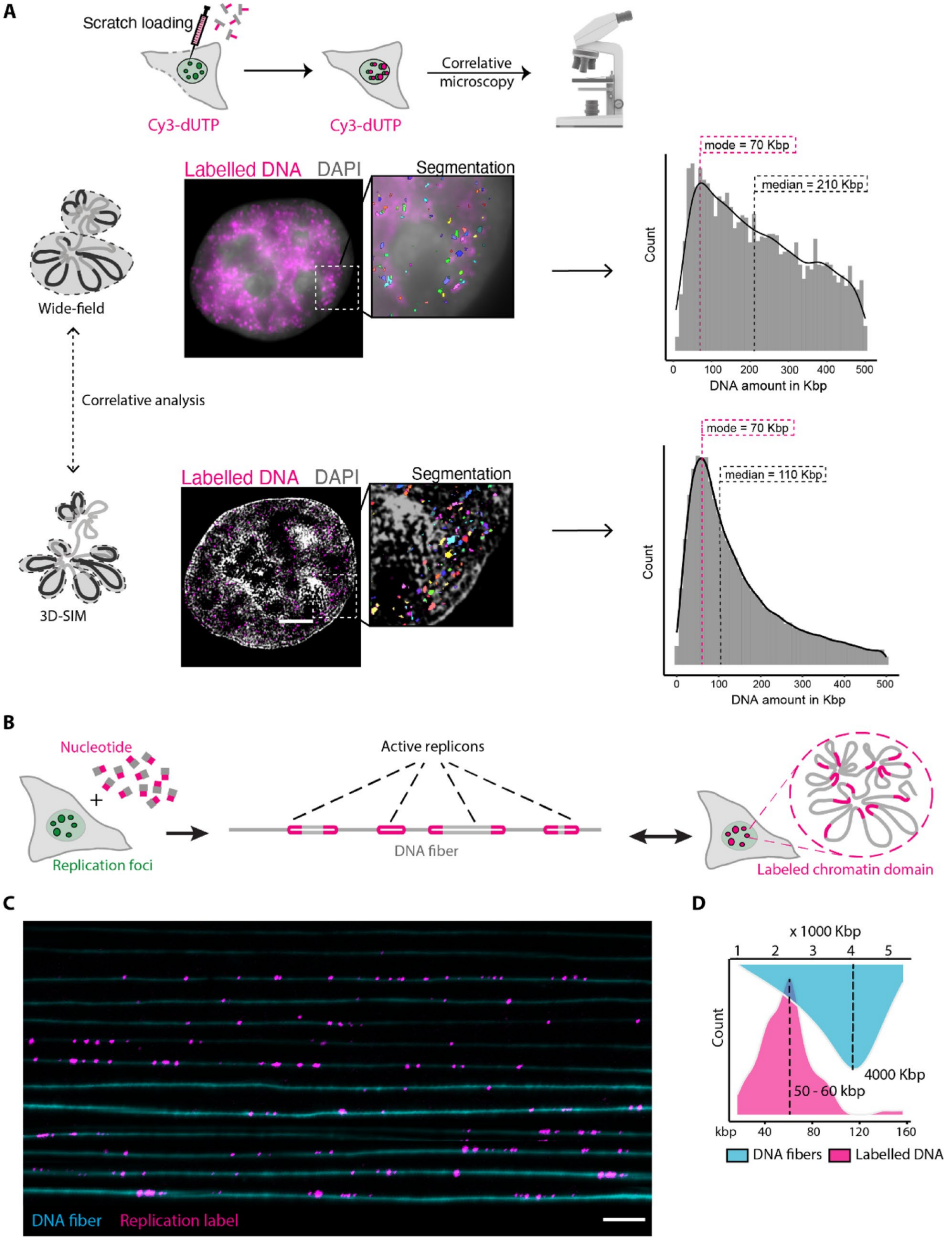
Quantification and correlation of labeled replication domains using microscopy and single molecule DNA fibers. **A** HeLa K GFP-PCNA cells were labeled with Cy3-dUTP (magenta) (Supplementary Table 2) using scratch loading technique and were then fixed using 3.7% paraformaldehyde for 15 min and DNA was stained using DAPI. The cells were then imaged using the DeltaVision OMX microscope for both GFP-PCNA and Cy3-dUTP (Supplementary Tables 1, 2, 3, 4) and processed to display corresponding widefield and 3D-SIM images. GFP-PCNA patterns were used to determine the cell cycle stage and the cell cycle correction factor (Supplementary Figs. 3, 4). Representative images of DNA (DAPI—gray) and labeled chromatin (Cy3-dUTP—magenta) in WF and 3D-SIM resolution  are shown. Individual cells were segmented, analyzed, and corrected for genome size and the histograms representing the quantified DNA amount per focus (*N* = 30) was plotted. The mode ± 5 bin of the histogram was represented in the figure (DNA quantification of labeled chromatin, Supplementary Figs. 3, 4, 5). The statistics of the histogram are shown in Supplementary Table 6. Scale bar 5 µm. **B** Single molecule DNA fiber experiment of DIG-dUTP labeled cells was performed to estimate the labeling efficiency of cells and correlate the DNA domain quantification using microscopy (DNA combing, Supplementary Figs. 6, 7, Supplementary Tables 3, 4). **C** Representative image of a single linearly stretched DNA fiber (cyan) and the labeled replication foci (DIG-dUTP: magenta). **D** The DNA fiber length and the labeled replication foci in kbp were plotted using the calibration measurements performed using lambda DNA (Supplementary Fig. 7). Scale bar 100 kbp

To quantify the DNA domain sizes, we subjected HeLa K GFP-PCNA cells to scratch loading with Cy3-dUTP followed by chemical fixation using formaldehyde (DNA quantification of labeled chromatin, Supplementary Tables 1, 2). The total DNA was subsequently stained with DAPI. We performed fixed cell imaging of DAPI (total DNA), GFP-PCNA (green), and Cy3-dUTP (labeled chromatin) and imaged the whole nuclear volume in SIM and WF resolutions (voxel size 41 ×  41 ×  125 nm). Then, we segmented both the entire nucleus and the individual labeled chromatin foci within the same cell. The fraction of DAPI intensity within the segmented replication focus (IRFi) relative to the total DNA intensity within the cell (IDNA total) provided the amount of DNA per labeled chromatin focus (DNA quantification of labeled chromatin, Fig. 2A, Supplementary Fig. 5). The DNA content (kbp) present in each labeled foci for SIM and WF images (*N* = 30) was plotted as a histogram where the x-axis represents the DNA amount present per focus (kbp) and the y-axis represents the count (Fig. 2A). The mode and median values of the histogram are indicated in Fig. 2A. We observed that in WF microscopy, a large number of labeled foci with a DNA content ranging from 100 to 500 kbp of DNA (median 210 kbp), which correspond to TAD-like structures (Giorgetti and Heard 2016). Whereas, using super-resolution microscopy (SIM), a large number of labeled foci have a size between 100 and 200 kbp of DNA (median 110 kbp), which corresponds to smaller loop like structures as shown by previous studies (median 185 kbp) (Rao et al. 2014; Mamberti and Cardoso 2020). Therefore, we propose that replication labeled DNA domains resolved by conventional WF microscopy correspond to TAD-sized domains whereas DNA domains resolved by 3D-SIM correspond to chromatin loops or sub-TADs. This is solely based on DNA content but not DNA sequence and need not correspond to TADs and loops identified by Hi-C methods.

To validate our results with an orthogonal method, we employed DNA fiber combing to quantify the labeled chromatin domain sizes using a single molecule DNA fiber method (Parra and Windle 1993; Bensimon et al. 1994; Jackson and Pombo 1998; Daigaku et al. 2010; Técher et al. 2013; Moore et al. 2022). This allowed us to visualize the labeled DNA domains and translate the 3D chromatin structures into linear DNA fibers (Fig. 2B). To get significant results, we needed to optimize the DNA combing technique to obtain long stretches of DNA fibers up to 4 Mbp (commonly the fibers break after a few hundreds of kbps) to be able to visualize multi-loop domains. Briefly, we labeled HeLa K cells with 100 µM DIG-11 dUTP nucleotides using electroporation to enable nucleotides to enter cells. The cells were then allowed to recover from electroporation overnight and DNA strands were extracted into the DNA combing buffer (DNA combing, Supplementary Fig. 6). Using antibodies we detected the incorporated DIG-11dUTP signal (magenta) on the linear single genomic DNA fibers (YOYO, cyan) (Fig. 2C). We performed the calibration of DNA stretching using the Genomic Vision combing machine to determine the stretching factor using lambda DNA (48.5 kbp) and obtained a stretching factor of 1 µm = 2 kbp (DNA combing, Supplementary Fig. 7). We then used the calibration results to measure and plot a histogram with the size of DNA fibers (cyan) in kbp and the size of labeled nucleotides in kbp (magenta) present along the DNA fibers (Fig. 2D). The DNA fiber results showed that the labeled DNA domains are between 50 and 100 kbp in size, which corresponds to our DNA content measurements using 3D-SIM imaging.

### Correlative chromatin motion analysis shows that nano-foci chromatin loops are more mobile than larger TAD-like structures

**Fig. 3:**
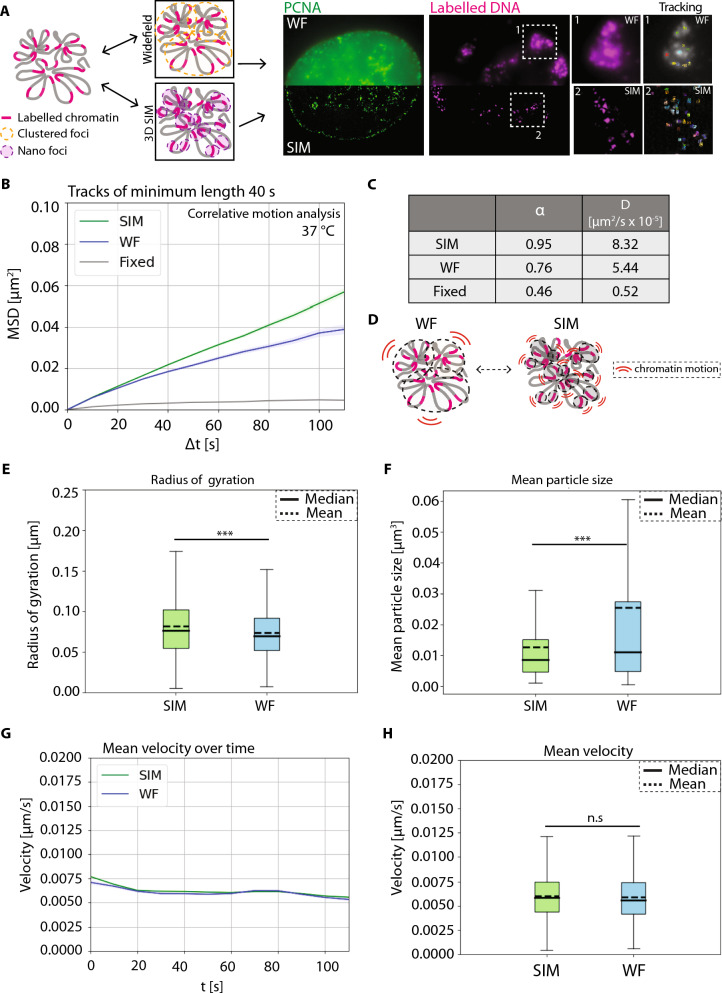
Correlative chromatin mobility of labeled DNA in HeLa K cells at widefield and structured illumination microscopy resolution. **A** HeLa K cells expressing GFP-PCNA were labeled with ATTO-590-dUTP (Methods, Supplementary Tables 1, 2) using scratch loading. After a few cell cycles, live cells were imaged in 3D and processed for structured illumination microscopy (SIM) and widefield (WF) resolutions, and time-lapse movies (frame interval of 10 s) of both GFP-PCNA (green) and labeled chromatin (magenta) were obtained (Supplementary Table 4). Representative images of HeLa K cells with PCNA (green) and labeled chromatin (magenta) in SIM and WF are shown. Inserts (white—1, 2) represent the zoom of chromatin imaged with SIM and WF resolutions, respectively. The zoomed inserts also show the computed tracks of chromatin over time (Supplementary Fig. 8, Videos 1, 2, 3, 4). **B** Non-rigid registration of the time-lapse movies using PCNA-GFP was performed to correct for the movement of cells (Methods, Supplementary Fig. 9). The registered movies were then used to detect labeled chromatin foci (Methods, Supplementary Fig. 10). These chromatin foci from the time-lapse movies of both SIM and WF were then analyzed to obtain the mean squared displacement curves (MSD, µm^2^) over time intervals (s) (Supplementary Fig. 8). MSD curves (µm^2^) over time intervals (s) for SIM (green), WF (blue), and control fixed cells (gray) were then plotted. **C** The table details the values of the anomalous diffusion coefficient α and the diffusion coefficient* D* (µm^2^/s × 10^−5^). **D** Illustration of labeled chromatin and its chromatin motion in SIM and WF. **E**, **F** Radius of gyration (µm) and mean particle size (µm^3^) for SIM (green) and WF (blue) chromatin domains (Supplementary Fig. 11). There is a highly significant difference between the SIM and WF foci (*p* < 0.001). The median and mean of the measurements are indicated in the figure. **G** Mean velocity (µm/s) of labeled chromatin foci for SIM (green) and WF (blue) plotted as a curve over time (s). **H** Mean velocity (µm/s) of labeled chromatin foci for SIM (green) and WF (blue) plotted as a box plot. There is a highly significant difference between the SIM and WF foci (*p* < 0.001). The median and mean values of the measurements are indicated in the figure. The statistics of the plots are shown in the figure and listed in Supplementary Table 6. Scale bar 5 µm. Also see Videos 1, 2, 3, 4

To perform correlative chromatin motion analysis, we imaged live HeLa K GFP-PCNA cells labeled with ATTO-590dUTP using scratch loading and performed dual-channel, multi-resolution, 3D sub-volume time-lapse imaging (frame interval of 10 s, 7 z-sections, 12 frames), to analyze and compare chromatin motion at different resolutions (Fig. 3A, Cells, Microscopy). During the acquisition of time-lapse movies, we observed a significant movement and deformation of the cells. We overlaid the chromatin channel from time point 1 (T1, magenta) and the final time point (T12, cyan) and observed a unidirectional motion of chromatin foci, which corresponds to cell movement (Supplementary Fig. 8). As there are fewer labeled chromatin foci in the nuclear sub-volumes, identification of nuclear boundaries was not possible in the chromatin channel. Therefore, we utilized the PCNA channel to segment the nuclear outlines. To register the time-lapse movies, we performed affine registration using our previous deep learning method to correct for rotational and translational motion of cells (Celikay et al. 2022). To correct for deformations of the nucleus during time-lapse microscopy, we used non-rigid registration after affine registration (Balakrishnan et al. 2019) improving the tracking accuracy considerably (Registration of live-cell image data, Video 2). To ensure that the particle detection algorithm only detects true chromatin foci, but not high-frequency noise artifacts generated during the SIM reconstruction, we used fixed control cells to detect any small-scale structures and their motion (Supplementary Fig. 9-A). This allowed us to optimize the particle detection threshold to detect true chromatin foci and perform tracking reliably (Supplementary Fig. 9-A, Methods). We also checked the number of chromatin foci detected over time in SIM and WF time-lapse movies. We observed a threefold increase in the number of foci detected between higher and lower resolution imaging (Supplementary Fig. 9-B, C). Having these controls, we then performed correlative motion analysis to obtain MSD (µm^2^) over time intervals in seconds (s) (Supplementary Fig. 10). We plotted the MSD curves for SIM (green), WF (blue), and fixed cells (gray) over time intervals (Fig. 3B, Video 3). In the SIM resolution datasets, we measured a diffusion constant (*D*) of 8.32 × 10^−5^ µm^2^/s with an alpha value (α) of 0.95, whereas with WF resolution the diffusion constant was lower with a* D* of 5.44 × 10^−5^ µm^2^/s and an alpha value (α) of 0.76 (Fig. 3C). We found that the chromatin nano-foci structures or loops imaged at SIM resolution are considerably more mobile than the clustered TAD-like domains imaged at WF resolution (Fig. 3D, Videos 3, 4). We next measured the radius of gyration (µm), which describes the extent of motion (*R*_g_) of a chromatin polymer over time and visualized it using box plots. We observed that chromatin foci in SIM (green) (mean 0.0818, median 0.0763) have a higher radius of gyration compared to chromatin foci in WF (blue) (mean 0.0738, median 0.0699) (*p* < 0.001) (Fig. 3E). We measured the mean particle size (in µm^3^), which allowed us to correlate the radius of gyration with the particle size and plotted these using box plots. We observed that chromatin foci in SIM (green) (mean 0.01267, median 0.008685) have significantly lower volumes compared to chromatin foci in WF (blue) (mean 0.02564, median 0.01121) (*p* < 0.001) (Fig. 3F). As a result of the higher chromatin domain sizes, we see a lower radius of gyration for chromatin foci in WF compared to SIM.

We then asked the question whether the mean velocity (µm/s) of chromatin foci in SIM is higher than for WF. We plotted the velocity curves and box plots for SIM (green, mean 0.006042, median 0.005854) and WF (blue, mean 0.005895, median 0.005598, and *p* = 0.09825) and observed no significant difference between the mean velocities of chromatin at different resolutions (Fig. 3G, H). We also measured the track straightness and the start-to-end distance and observed a significant difference between SIM (green) and WF (blue) chromatin foci (*p* < 0.005) (Supplementary Fig. 11, Supplementary Table 6). In summary, our results characterize the chromatin motion differences in smaller loop chromatin domains and larger TAD-like domains, which helps us to relate chromatin dynamics in the context of chromatin higher-order organization.

### Chromatin motion reduces in S phase relative to G1/G2

**Fig. 4:**
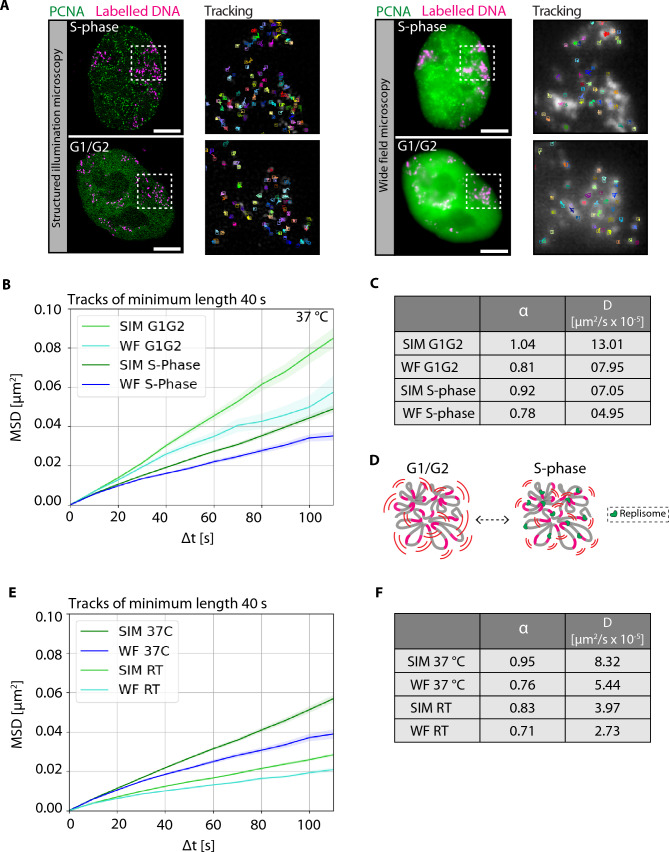
Correlative motion analysis of labeled chromatin during the cell cycle stages and depending on temperature. **A** HeLa K cells with labeled DNA were used to obtain live-cell time-lapse movies (frame interval of 10 s) (Methods, Supplementary Tables 1, 2). Correlative imaging of two channels GFP-PCNA and labeled chromatin in SIM and WF in 3D were obtained (Supplementary Table 4). During S phase, PCNA accumulates within the nucleus at sites of active DNA replication and exhibits a distinct puncta pattern. During G1 and G2, GFP-PCNA is diffusely distributed throughout the nucleus. GFP-PCNA patterns were used to classify cells in different cell cycle stages (Supplementary Fig. 3). The representative images show GFP-PCNA (green) and labeled DNA (magenta) for both SIM and WF resolutions. The tracks of chromatin mobility of the white inserts are shown in zoom. **B** The registered time-lapse movies were used to detect chromatin foci of both SIM and WF and then analyzed to obtain the mean squared displacement curves (MSD, µm^2^) over time intervals (s) (Supplementary Figs. 8, 13). The MSD curves over time intervals (s) were plotted for S phase and G1/G2 for both SIM and WF. **C** The table details the values of the anomalous diffusion coefficient α and the diffusion coefficient* D* (µm^2^/s × 10^−5^). **D** Illustration of labeled chromatin and in S phase and G1/G2. **E** During live cell imaging of chromatin labeled HeLa K GFP-PCNA cells, experiments at two different temperatures (37 ºC and room temperature (RT)) were performed. The MSD curves over time intervals (s) were plotted for imaging at 37 °C and RT for both SIM and WF (Supplementary Fig. 12). **F** The table details the values of the anomalous diffusion coefficient α and the diffusion coefficient* D* (µm^2^/s × 10^−5^). The statistics of the plots are shown in figure and listed in (Supplementary Table 6). Scale bar 5 µm. See Video 5

To determine how the global chromatin dynamics change during cell cycle progression at multiple resolutions (SIM and WF), we utilized chromatin labeled cells and GFP-PCNA to assign the cell cycle stage during S phase (Leonhardt et al. 2000; Sadoni et al. 2004; Chagin et al. 2016). First, the cells were annotated according to the different cell cycle stages (G1, S, G2) based on the PCNA subnuclear pattern (Methods). PCNA forms puncta or foci at the active replication sites during S phase, which was used to classify cells in S phase. We were able to distinguish between G1 and G2 cells, even though they exhibit a similar diffused PCNA subnuclear distribution, based on the information on the preceding cell cycle stage from the time-lapse analysis performed after labeling (Microscopy). Specifically, cells with diffusely distributed PCNA signal, which had previously undergone mitosis were in G1 phase, whereas the ones with similar diffuse PCNA pattern that had previously undergone S phase (punctated PCNA pattern) were classified as being in G2 phase. The PCNA signal was also used to segment the nucleus, and individual chromatin foci were detected within the segmented nuclei. Probabilistic tracking was performed to obtain individual chromatin trajectories at SIM and WF resolutions. Representative images of GFP-PCNA (green) and labeled DNA (magenta) of mid S phase and G1/G2 cells along with chromatin tracks are shown in Fig. 4A, Video 5. We performed probabilistic chromatin tracking (Supplementary Fig. 10, Methods) to obtain the MSD (µm^2^) over time intervals (s) of S phase versus non-S phase at different resolutions (Fig. 4B, Supplementary Fig. 12-A). The curves show significantly higher chromatin dynamics in SIM G1/G2 [light green, diffusion coefficient (*D*) = 13.01× 10^−5^ µm^2^/s] compared to SIM S phase [dark green, diffusion coefficient (*D*) = 7.05 × 10^−5^ µm^2^/s]. We also observe a significantly higher chromatin dynamics in WF G1/G2 (light blue, diffusion coefficient (*D* = 7.95 × 10^−5^ µm^2^/s) compared to WF S phase [dark blue, diffusion coefficient (*D*) = 4.95 × 10^−5^ µm^2^/s] (Fig. 4C, Supplementary Fig. 12-A). In summary, we see reduced chromatin motion in S phase compared to non-S phase independent of the resolution. These results concur with our previous observations at confocal resolution (Pabba et al. 2023). It is also interesting to see that the S phase chromatin mobility in SIM is lower than the G1/G2 chromatin mobility in WF, showing that there are more constraints in chromatin loop motion in S phase than the larger TAD-like domains.

### Chromatin motion reduces with decreasing temperature at different resolutions

To analyze the changes in chromatin mobility at different temperatures, we acquired 3D live-cell time-lapse images (frame interval of 10 s) of HeLa K GFP-PCNA, and labeled chromatin (ATTO590-dUTP) at 37 °C and RT (Microscopy, Supplementary Tables 1, 2, 4) and plotted the MSD curves for both conditions 37 °C and RT at different resolutions (Fig. 4E, Supplementary Fig. 12-B). The MSD curves show significantly higher chromatin dynamics in SIM at 37 °C [dark green, diffusion coefficient (*D*) = 8.32 × 10^−5^ µm^2^/s] compared to SIM at RT [light green, diffusion coefficient (*D*) = 3.97 × 10^−5^ µm^2^/s]. We also observe that the results show significantly higher chromatin dynamics in WF at 37 °C (dark blue, diffusion coefficient (*D*) = 5.44 × 10^−5^ µm^2^/s) compared to WF at RT [light blue, diffusion coefficient (*D*) = 2.73 × 10^−5^ µm^2^/s] (Fig. 4E, F; Supplementary Fig. 12-B). We found that regardless of the resolution, the chromatin mobility at 37 °C is significantly higher than at RT.

### Motion subpopulation analysis of chromatin shows different diffusion behaviors

**Fig. 5:**
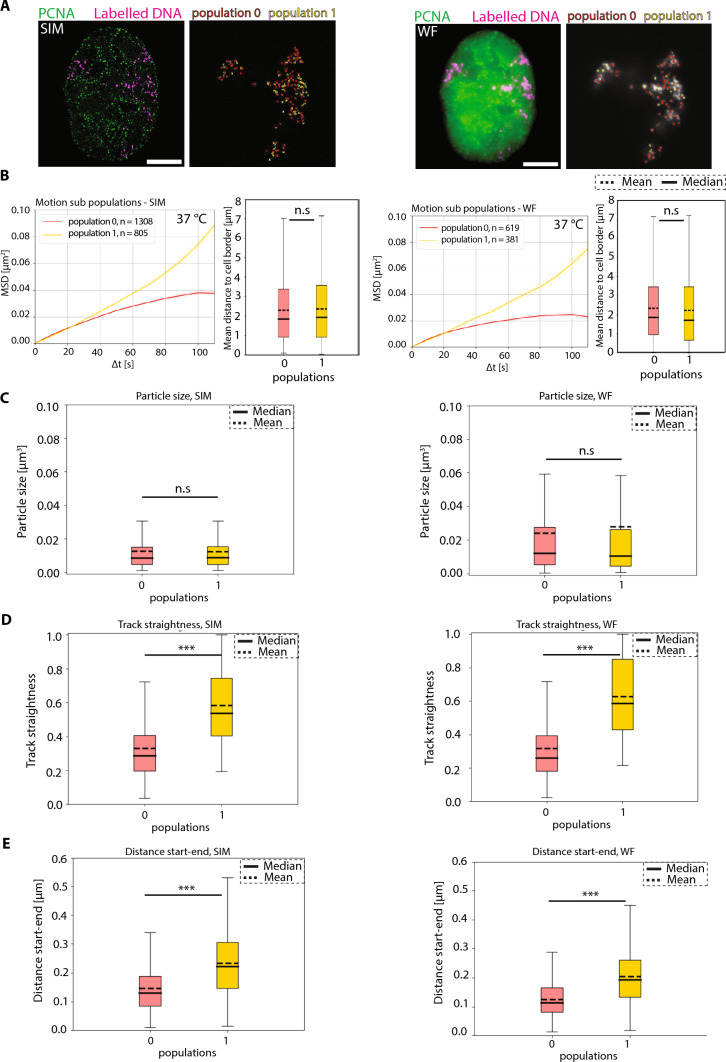
Subpopulation classification of chromatin motion. **A** Representative images of HeLa K GFP-PCNA (green) and labeled chromatin (magenta) live cells in SIM and WF. The computed chromatin tracks were classified into population 0 (red) and population 1 (yellow) based on k-means clustering of the α values. The tracks were colored in red (population 0) and yellow (population 1) in both SIM and WF images. The subpopulation mean values of α for SIM are 0.56 and 1.66 for population 0 (red) and population 1 (yellow), respectively. The subpopulation mean values of α for WF are 0.48 and 1.63 for population 0 (red) and population 1 (yellow), respectively. We analyzed different parameters for different subpopulations and plotted them (Supplementary Fig. 13). **B** Mean squared displacement (MSD, µm^2^) over time intervals (s) for population 0 (red) and population 1 (yellow) was plotted for SIM and WF time-lapse movies. We also plotted the mean distance (µm) to the cell border for the chromatin tracks of the subpopulations. There is no significant difference in mean distance (µm) to the cell border between the subpopulations. The mean and median of the box plots are also indicated. The statistics of the plots are shown in figure and listed in (Supplementary Table 6). **C** The mean particle size (µm^3^) of SIM and WF chromatin domains of population 0 (red) and population 1 (yellow) are plotted as box plots. There is no significant difference between the populations. The mean and median of the box plots are also indicated. **D** The track straightness of SIM and WF chromatin domains of population 0 (red) and population 1 (yellow) are plotted as box plots. There is a highly significant difference between the two populations (*p* < 0.001). The mean and median of the box plots are also indicated. **E** The distance start–end (µm) of SIM and WF chromatin domains of population 0 (red) and population 1 (yellow) are plotted as box plots. There is a highly significant difference between the two populations (*p* < 0.001). The mean and median of the box plots are also indicated. The statistics of the plots are shown in figure and listed in (Supplementary Table 6). Scale bar 5 µm

We next asked the question whether chromatin foci at SIM resolution (loops) or WF resolutions (“TADs”) behave differently in terms of chromatin motion. To answer this, we classified the chromatin foci tracks into two distinct motion populations (0—red, 1—yellow) using k-means clustering of the anomalous diffusion coefficient α at both SIM and WF resolutions (Fig. 5A). The subpopulation mean values of α for SIM are 0.56 and 1.66 for population 0 (red) and population 1 (yellow), respectively, and the subpopulation mean values of α for WF are 0.48 and 1.63 for population 0 (red) and population 1 (yellow), respectively (Supplementary Table 6). For these subpopulations, we computed and plotted the MSD (µm^2^) over time intervals (s) for SIM and WF microscopy (Fig. 5B). We observed that chromatin foci of population 0 (red) exhibit a constrained diffusion behavior at both SIM (mean α = 0.56) and WF (mean α = 0.48) resolutions as suggested by various studies (Marshall et al. 1997; Heun et al. 2001; Chuang and Belmont 2007; Oliveira et al. 2021; Pabba et al. 2023). To our surprise, we also observed a minor population of chromatin foci or population 1 (yellow) showing directed diffusion behavior at both SIM (mean α = 1.66) and WF (mean α = 1.63) resolutions (Fig. 5B, Supplementary Table 6). This effect was predominant in higher-resolution chromatin loops domains than compared to lower-resolution TADs (Fig. 5B).

We then asked the question whether there is a relation between the two motion populations and their spatial location. To answer this, we plotted and visualized the mean distance (µm) of each chromatin foci from the cell border of population 0 (red) and population 1 (yellow) at both resolutions. We observed no significant difference between the two populations in SIM and WF resolution (Fig. 5B). We then measured and visualized different parameters such as mean particle size (µm^3^), track straightness, and distance start–end (µm) for SIM and WF (population 0 and population 1). We observed no significant difference in particle size (µm^3^) between population 0 and population 1 for both SIM and WF chromatin foci (Fig. 5C, Supplementary Fig. 13, Supplementary Table 6). It is interesting to observe that the track straightness and distance start–end (µm) for population 1 was significantly higher than population 0, irrespective of SIM or WF (Fig. 5D, E; Supplementary Fig. 13, Supplementary Table 6). These results show that a minor population of chromatin foci exhibit a directed diffusion behavior, while most of the foci exhibit a constrained diffusion behavior. It leads us to ask the question whether these chromatin foci behavior is affected by their spatial location within the nucleus.

### Chromatin motion analysis show spatial differences with the nuclear border and the nuclear interior exhibiting decreased motion

**Fig. 6:**
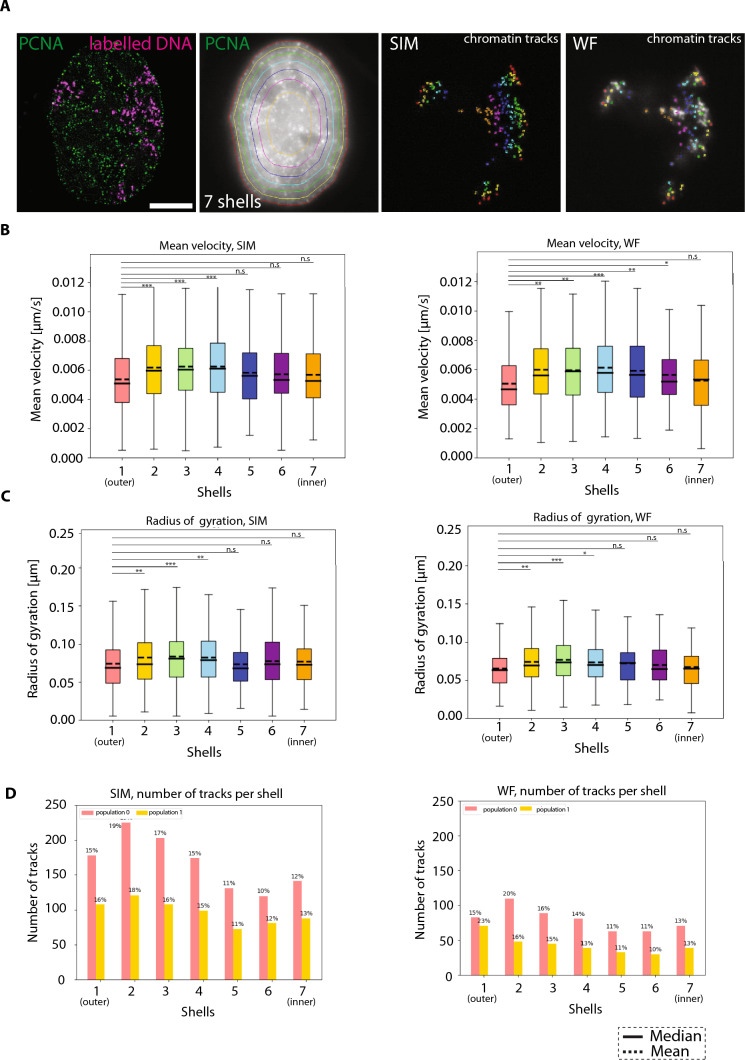
Location-based analysis of chromatin domains. **A** Representative images for chromatin classification (shells with equal volume) based on the spatial location of labeled foci. HeLa K GFP-PCNA cells with overlaid GFP-PCNA signal (green) and labeled chromatin (magenta) are shown. The PCNA signal was used to identify the nuclear border, and the whole nuclear volume was divided into 7 shells having equal volume. The 7 shells are represented in different colors. The chromatin tracks of SIM and WF within each shell were then marked and colored according to the shell (Supplementary Fig. 14). **B** The mean velocity (µm/s) of labeled chromatin domains subdivided into different shells are plotted in a box plot and the median and mean values of mean velocity are indicated. The significance test between chromatin in shell 1 (outer) versus other shells were plotted (Supplementary Fig. 13). **C** The radius of gyration (µm) of labeled chromatin domains subdivided into different shells are plotted in a box plot and the median and mean values of mean velocity are indicated. The significance test between chromatin in shell 1 (outer) versus other shells was plotted. **D** The number of particles present in each shell and subdivided into population 0 (red) and population 1 (yellow) were plotted in percentages for both SIM and WF time-lapse videos. The statistics of the plots are shown in the figure and listed in (Supplementary Table 6). Scale bar 5 µm

It is intriguing to speculate that on the basis of its location chromatin behaves differently as a result of the crowdedness inside the nucleus. To determine if chromatin dynamics is influenced by its spatial positioning, we conducted a comprehensive spatial analysis of chromatin tracks at both SIM and WF resolutions. We utilized time-lapse imaging of HeLa cells expressing GFP-PCNA and labeled chromatin in 3D. This approach enabled us to segment the entire nucleus into concentric shells of equal volumes, allowing us to assess how chromatin dynamics may differ across distinct nuclear environments. We segmented the nucleus using PCNA signal into shells of equal volumes. To cover a large number of volumes, we performed the analysis from two shells up to 10 shells. Segmenting the nuclear volume into more than 10 shells resulted in shell dimensions close to the dimension of DNA foci. In all conditions, we measured the MSD (µm^2^), mean velocity (µm/s), radius of gyration (µm), alpha value (α), track straightness, and distance start–end (Supplementary Fig. 14). We color-coded the shells and chromatin to identify the chromatin tracks within different shells in both SIM and WF datasets. We observed no changes in our results when we adjusted the tracks present in different shells within SIM or WF to the same number. We reasoned that to measure potential nuclear border effects a distance of less than 1 µm of the nuclear periphery would be acceptable, which corresponded to having seven concentric shells (outer shell 653 nm on average) (outer shell—red, inner shell—orange, Fig. 6A). We then identified and plotted the number of chromatin tracks present. We observed that the chromatin tracks within the outer shell (close to nuclear periphery, shell 1) and inner shells (5, 6, 7) have significantly lower mean velocity and radius of gyration (µm) than the chromatin tracks present in shells 2, 3, and 4 in both SIM and WF resolutions (Fig. 6B, C; Supplementary Fig. 14). We then asked the question whether the difference in chromatin motion based on spatial positioning is related to the diffusion type or the motion subpopulation analysis we performed before. Therefore, we visualized the distribution of the number of tracks of motion population 0 (α < 1, red) and motion population 1 (α > 1, yellow) within each shell volume (shells 1–7) and plotted them (Fig. 6D). We also plotted the ratio of tracks (population 0/population 1) present within each shell as a pie chart and observed no bias of one subpopulation over the other in SIM chromatin foci (Fig. 6D, Supplementary Fig. 14). This led us to determine that chromatin located at the nuclear periphery and in the nuclear interior exhibits slower movement independent of the motion characteristics compared to chromatin positioned in between these locations.

### Chromatin motion of segregated chromosome territories is slower than individual loops or TAD-like structures

**Fig. 7:**
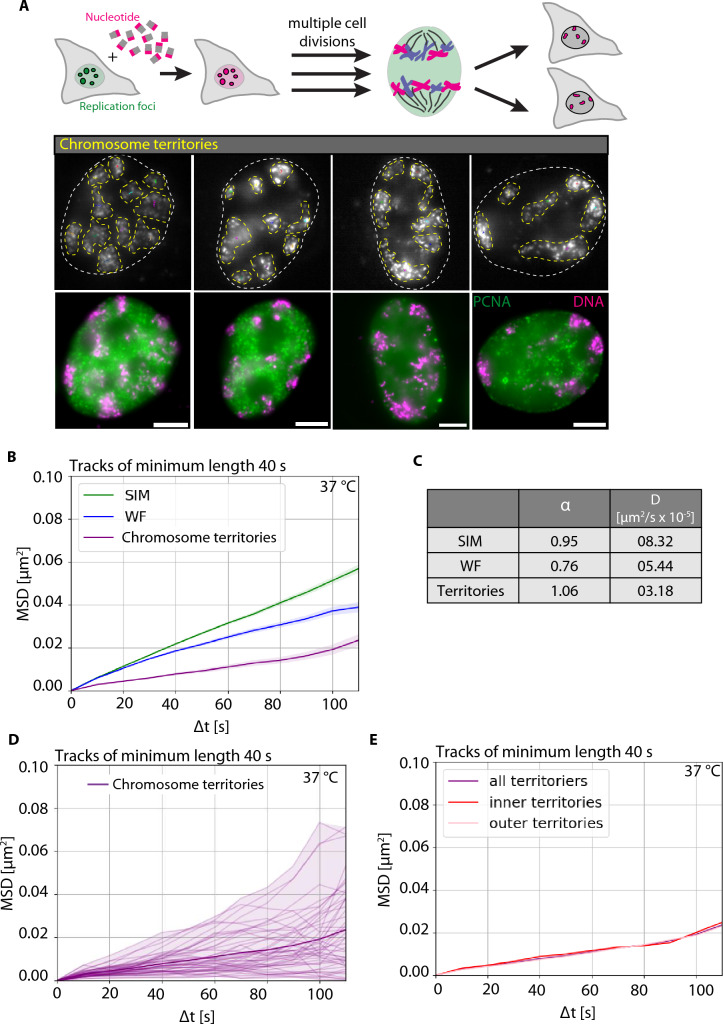
Mobility of chromosome territories. **A** Illustration of labeled DNA after segregation over several cell cycles resulting into individual chromosome territories labeled. Overlay image of HeLa K GFP-PCNA cells with PCNA (green) and labeled DNA (magenta). The borders of chromosome territories are marked with yellow dotted lines. The chromatin tracks of chromosome territories are overlaid. The white dotted lines represent the nuclear borders. **B** The chromosome territories were tracked as a whole chromosome from the widefield (WF) time-lapse movies to obtain the mean squared displacement curves (MSD, µm^2^) over time intervals (s) (Supplementary Fig. 8). The MSD curves (µm^2^) over time intervals (s) were plotted for SIM (green), WF (blue), and chromosome territories (purple) were then plotted. **C** The table details the values of the anomalous diffusion coefficient α and the diffusion coefficient* D* (µm^2^/s × 10^−5^). **D** Mean squared displacement curves (MSD, µm^2^) over time intervals (s) of individual chromosome territories were plotted. The dark curve represents the average MSD (µm^2^) of all chromosome territories. **E** MSD (µm^2^) over time intervals (s) of chromosome territories grouped as labeled chromosomes touching the nuclear border (outer territories, light red) and labeled chromosomes not touching the nuclear border (inner territories, red) was plotted. Scale bar 5 µm. See Video 6

There are many challenges involved in visualizing entire chromosomes in live cells. An advantage of employing the replisome (DNA replication) to integrate labeled deoxyribonucleotides into the genome is its random incorporation into both the leading and lagging strands (Jackson and Pombo 1998). Allowing cells to undergo multiple cell divisions results in the random segregation of individual chromosomes (few megabase pairs) with chromatin regions labeled in the initial cell cycle (Fig. 7A, Video 6). The segregated chromosome territories are clearly visible in cells after multiple cell divisions (magenta, boundaries marked by yellow dots, Fig. 7A). We identified the centroid of each chromosome territory in every image frame and conducted tracking over time. It is important to note that tracking all identified chromosome territories over time poses challenges, especially with chromosomes that exhibit variable shapes, making it difficult to determine their centroids for tracking (yellow dotted boundaries, Fig. 7A). In Fig. 7B, we plotted the MSD (µm^2^) over time intervals (s) for the chromosome territories (purple), overlaid with the SIM (green), WF (blue) DNA labeled (nano-)foci (Fig. 3). The DNA/chromatin motion in 3D-SIM resolution has a diffusion constant (*D*) of 8.32 × 10^−5^ µm^2^/s with an alpha value (α) of 0.95, in WF resolution has a diffusion constant (*D*) of 5.44 × 10^−5^ µm^2^/s with an alpha value (ɑ) of 0.76, whereas the chromosome territories exhibit a significantly lower diffusion constant (*D*) of 3.18 × 10^−5^ µm^2^/s with an alpha value (α) of 1.06 (Fig. 7C). It is clear that chromosome territories (serving as proxies for whole chromosomes) exhibit significantly less dynamics than individual loops (as observed in SIM) or TAD-like structures (as observed in WF). As we are tracking randomly segregated chromosomes and cannot identify which chromosome this is, we analyzed whether individual chromosome territories exhibit very different chromatin dynamics due to their different labeled sizes (Supplementary Fig. 15, Video 6) (Adey et al. 2013). We overlaid the MSD plots for individual chromosome territories (Fig. 7D). We observed a significant variation of diffusion constants across individual chromosome territories, and we hypothesize that this variability may be attributed to differences in chromosome sizes or their spatial positioning (Fig. 7D). As it is very difficult to label chromosome regions, we analyzed whether the spatial location of chromosome territories within the nucleus plays a role. For this, we separated the chromosome territories into two regions: the outer (light red) and inner (dark red) territories. This classification was determined by assessing whether the labeled regions were either near to or in direct contact with the nuclear border (identified using GFP-PCNA). We observed no significant differences in chromatin dynamics of chromosome territories whether or not they are present at the nuclear border (Fig. 7E).

In conclusion, we found that DNA mobility was higher at the individual loop level compared to the TAD level and even less so at the chromosome level. Regardless of the organization level, DNA motion was slowed down in the S phase of the cell cycle. Importantly, irrespective of the DNA organization level, the motion was complex, and we identified a population of DNA loops and TADs that exhibited directed movement while the majority depicted constrained movement. Our data also indicated spatial mobility differences highlighting that DNA structures (loops as well as TADs) at the nuclear periphery and the nuclear interior exhibited lower velocity and lower radius of gyration than at the intermediate locations. On the basis of these insights, we propose that DNA mobility is inherently linked to its organizational structure including its spatial distribution, and this impacts cellular processes.

### Supplementary Information

Below is the link to the electronic supplementary material.

Supplementary file1 (DOCX 15342 KB)

Supplementary file2 (AVI 997 KB)

Supplementary file3 (AVI 1776 KB)

Supplementary file4 (AVI 1481 KB)

Supplementary file5 (AVI 1036 KB)

Supplementary file6 (AVI 2261 KB)

Supplementary file7 (AVI 2332 KB)

## Data Availability

All data are available from the OMERO open microscopy environment public repository http://cc-omero.bio.tu-darmstadt.de/webclient/?show=dataset-903 and 10.48328/tudatalib-1398. All renewable biological materials and software will be made available upon request from M. Cristina Cardoso (cardoso@bio.tu-darmstadt.de) and Karl Rohr (K.Rohr@dkfz-heidelberg.de), respectively.
