# Supplementary material for: DNA choreography: correlating mobility and organization of DNA across different resolutions from loops to chromosomes
Source: Histochem Cell Biol. 2024 May 17;162(1-2):109–31. doi: 10.1007/s00418-024-02285-x (PMC11227476; doi:10.1007/s00418-024-02285-x)
Supplement: Supplementary file 1 — Supplementary file1 (DOCX 15342 KB) [file 418_2024_2285_MOESM1_ESM.docx]

**DNA choreography: correlating mobility and organization of DNA across different resolutions from loops to chromosomes**

Maruthi K. Pabba^1^*, Janis Meyer^2^*, Kerem Celikay^2^*, Lothar Schermelleh^3^, Karl Rohr^2^#, M. Cristina Cardoso^1^#

1. Department of Biology, Technical University of Darmstadt, Germany

2. Biomedical Computer Vision Group, BioQuant, IPMB, Heidelberg University, Germany

3. Department of Biochemistry, University of Oxford, United Kingdom

* these authors have contributed equally

# corresponding authors

Requests should be directed to [cardoso@bio.tu-darmstadt.de](mailto:cardoso@bio.tu-darmstadt.de) and [k.rohr@dkfz-heidelberg.de](mailto:K.Rohr@dkfz-heidelberg.de)

# **Supplementary figures**

**
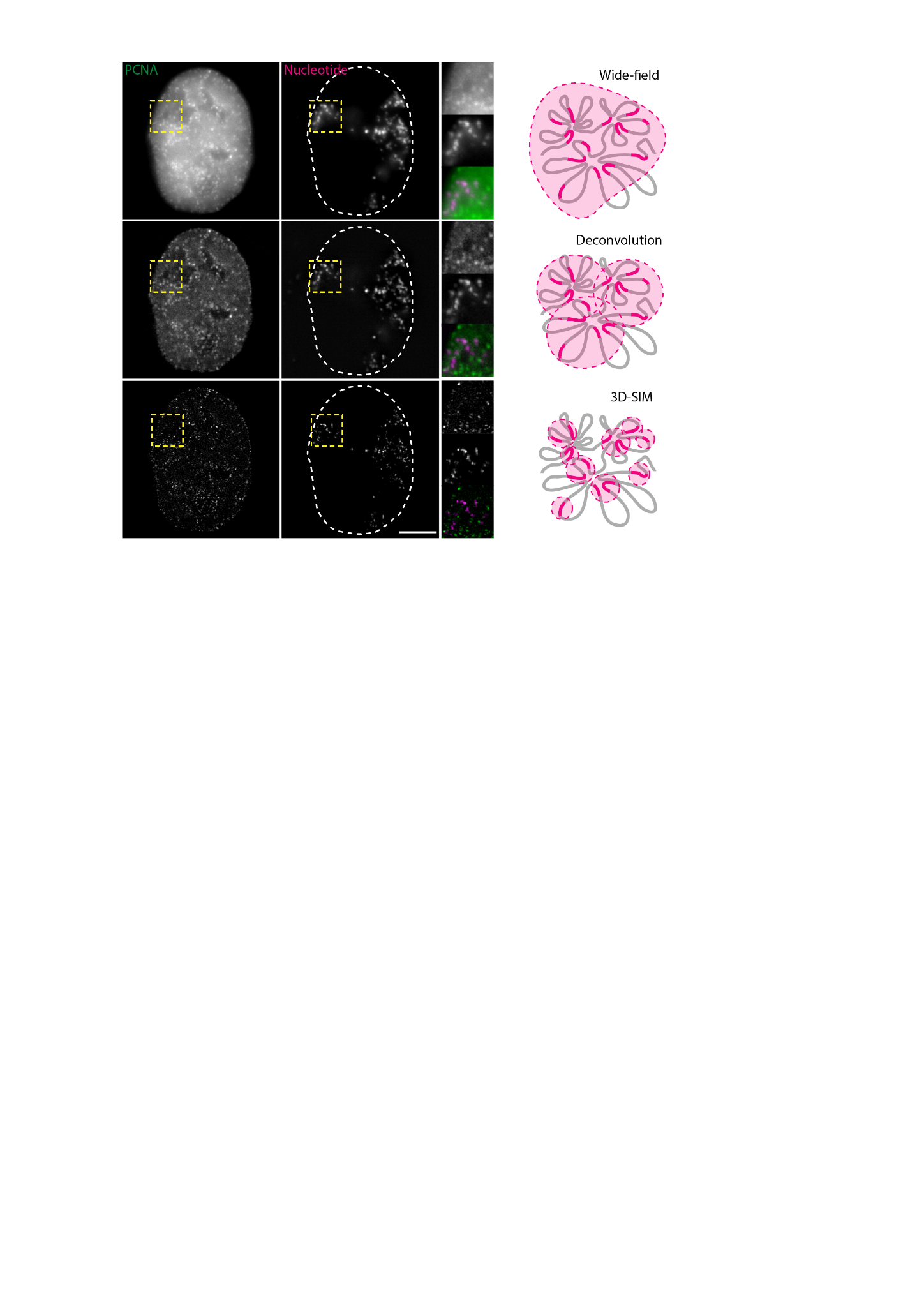
**

## Supplementary figure 1: Live cell simultaneous correlative imaging of HeLa K GFP-PCNA cells with fluorescently labeled chromatin at different modes of resolution.

The GFP-PCNA (green) and labeled DNA/chromatin (magenta) channels were imaged in 3D using simultaneous acquisition in Wide-Field (WF), deconvolved Wide-Field (deconv WF) and Structured Illumination Microscopy (SIM). The yellow inserts show the zoomed region for all channels. Scale bar: 5 µm.


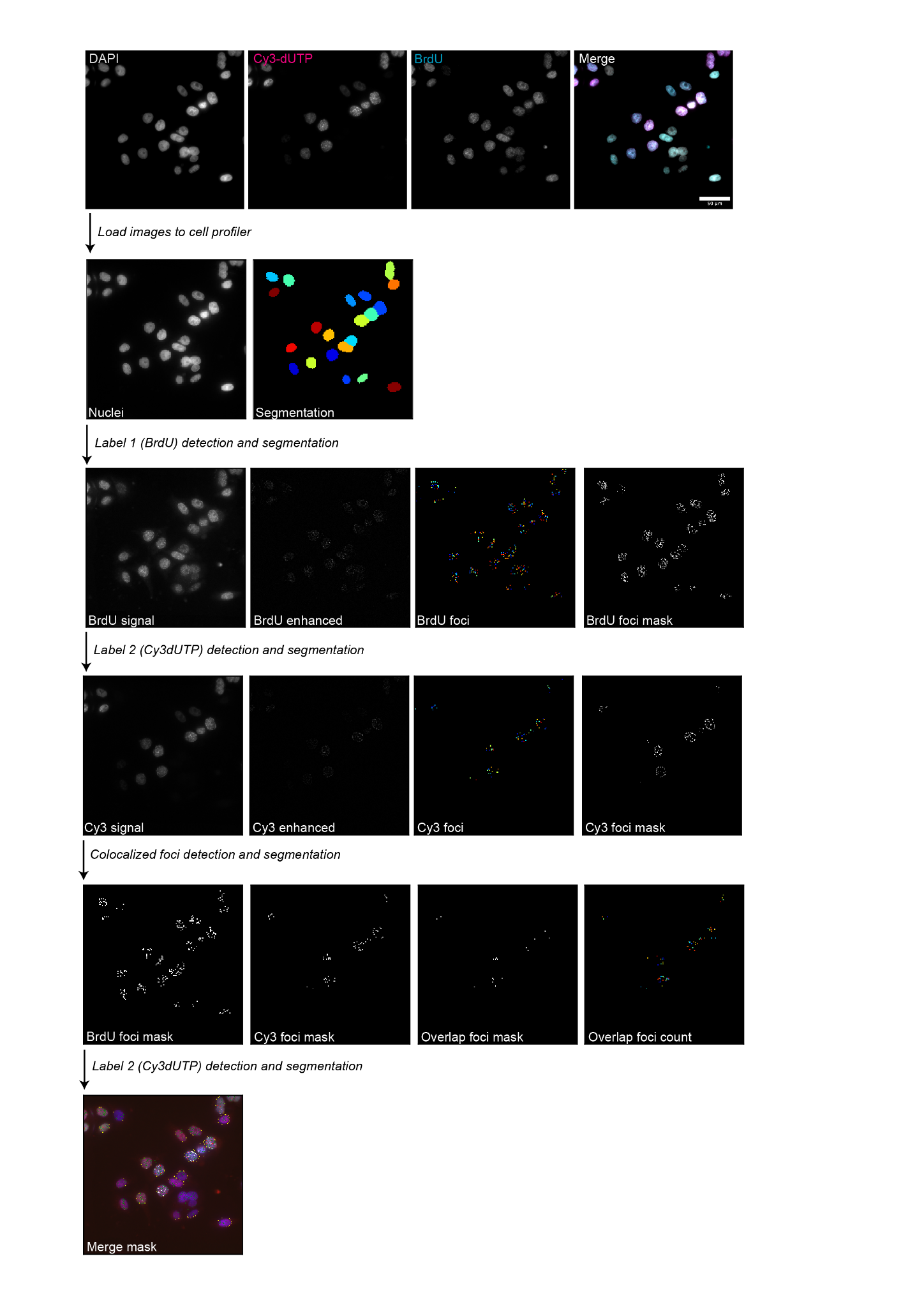


## Supplementary figure 2: Colocalization analysis to determine the nucleotide pulse length

## (A) Step by step description of analysis pipeline using cell profiler to calculate the percentage of colocalized nucleotides. (B) The labeling scheme to determine the DNA labeling duration using scratch loading, we first labeled cells with Cy3-dUTP (magenta) using scratch loading followed by different chase times (0’/10’/15’/20’/25’/30’), which was then followed by a second nucleoside pulse (BrdU - 40 µM) for 5 minutes to label cells. The cells were then fixed after a few hours and BrdU detection was performed (Methods, Supplementary table 3). (C) The cells were then imaged using a high throughput wide-field microscope (Supplementary table 4). We performed colocalization analysis of both labels to determine the overlap percentage over time, which was then plotted as bar plots. Scale: 10 µm.


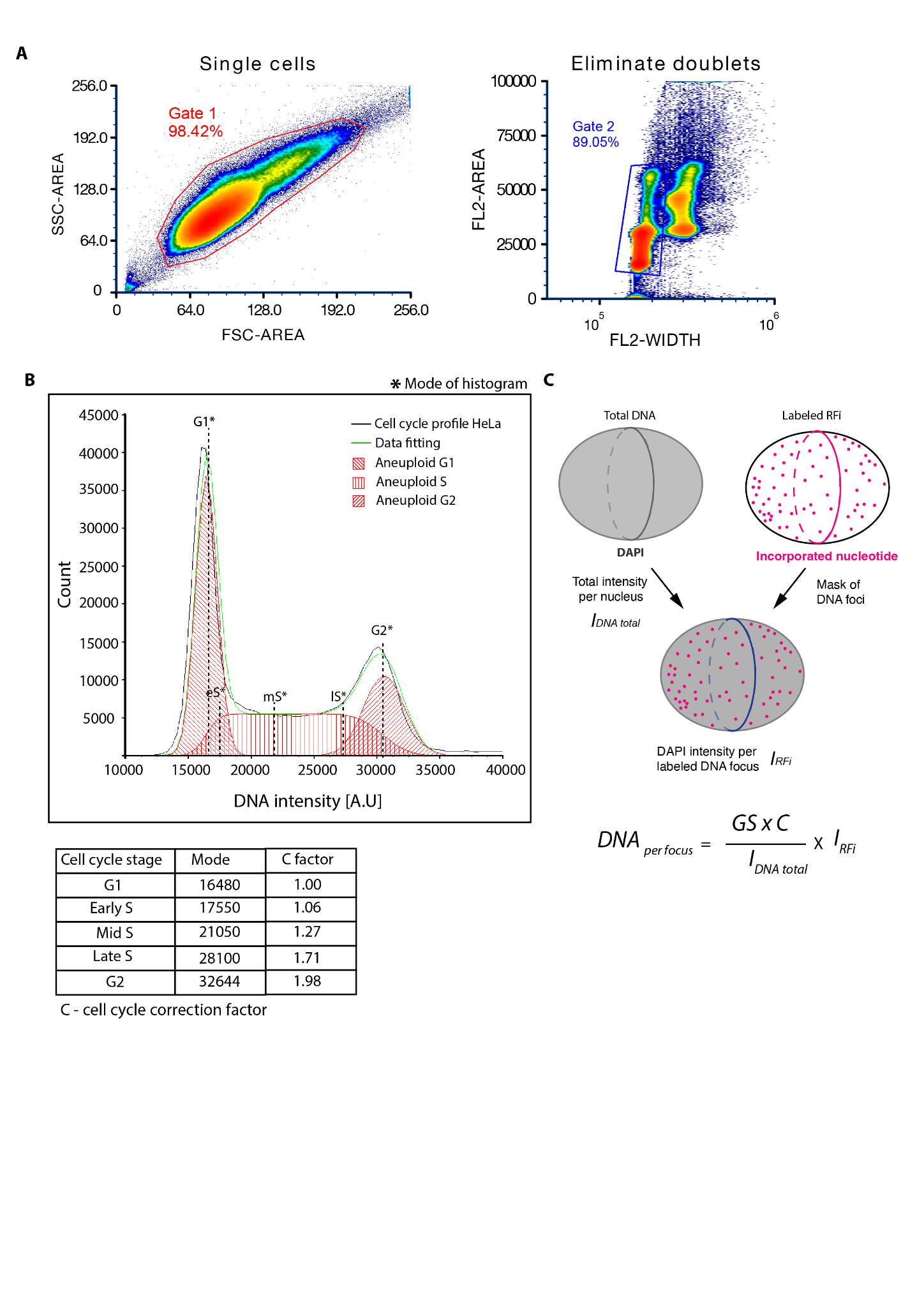


## Supplementary figure 3: Flow cytometry analysis of fixed HeLa K GFP-PCNA cells to determine the relative genome size (correction factor) during DNA replication using DNA intensity (methods, flow cytometry).


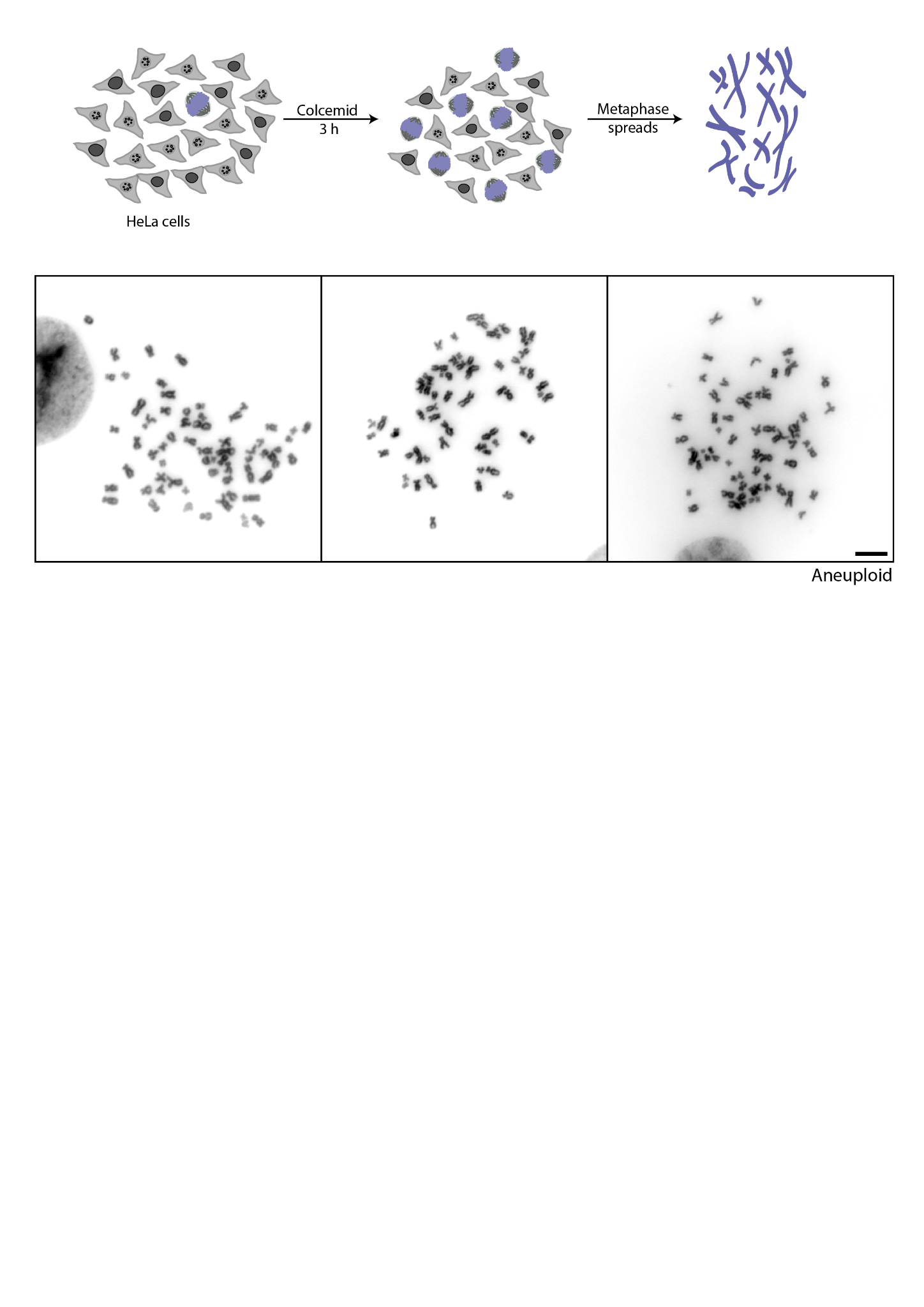


## Supplementary figure 4: Metaphase spreads of HeLa K GFP-PCNA cells to determine the ploidy in the cell population (methods, metaphase spreads). Scale bar: 10 µm.


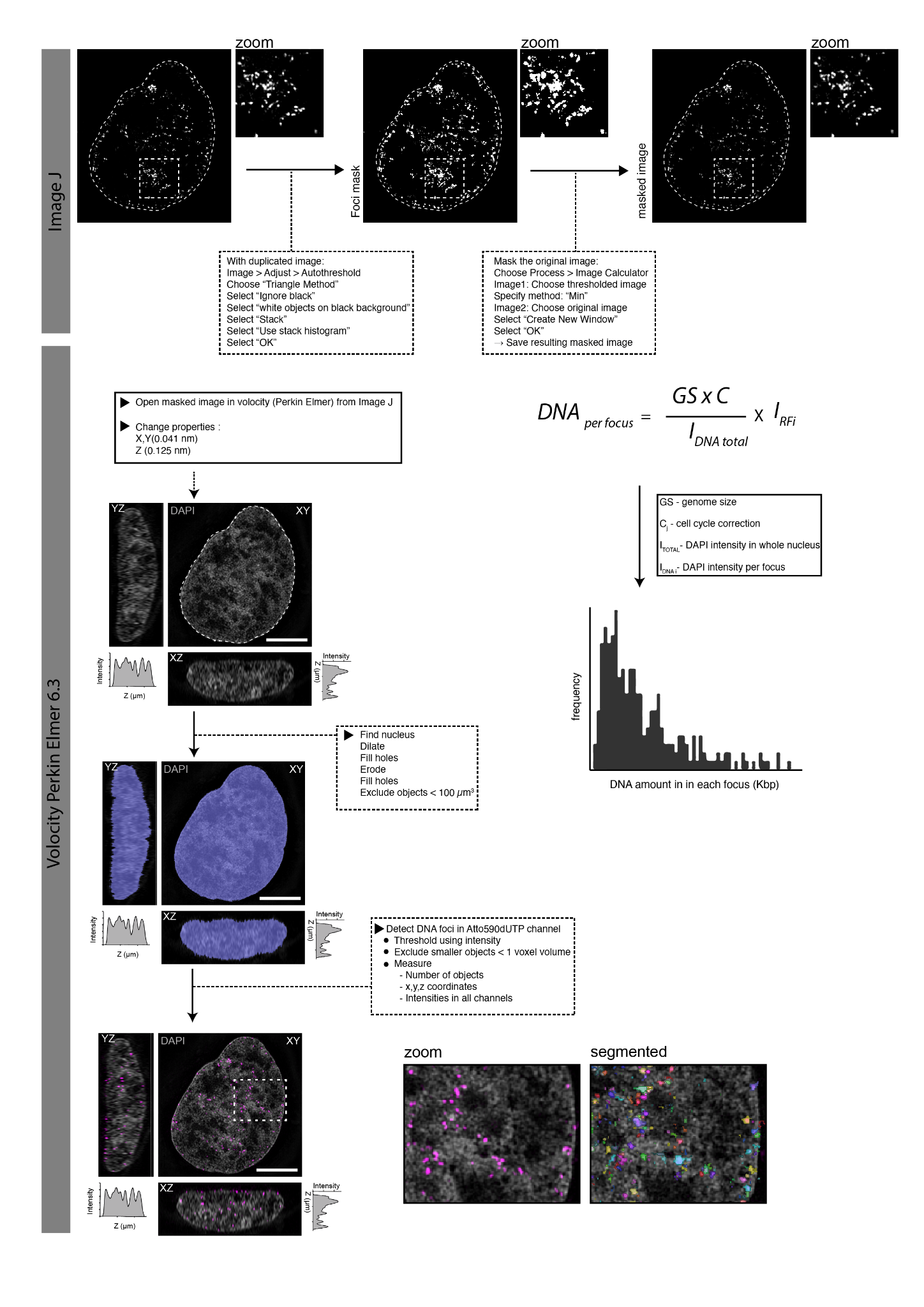


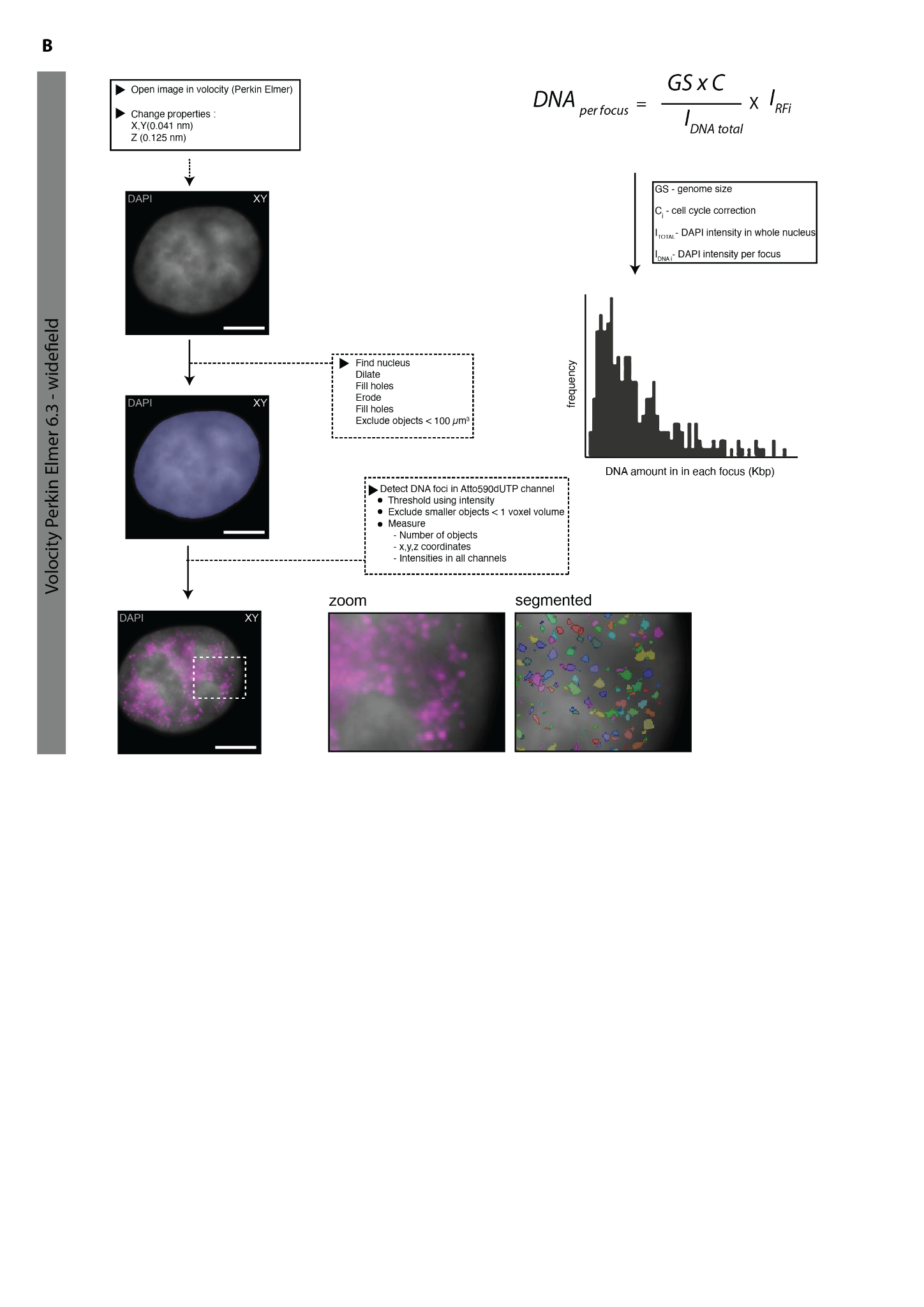


##
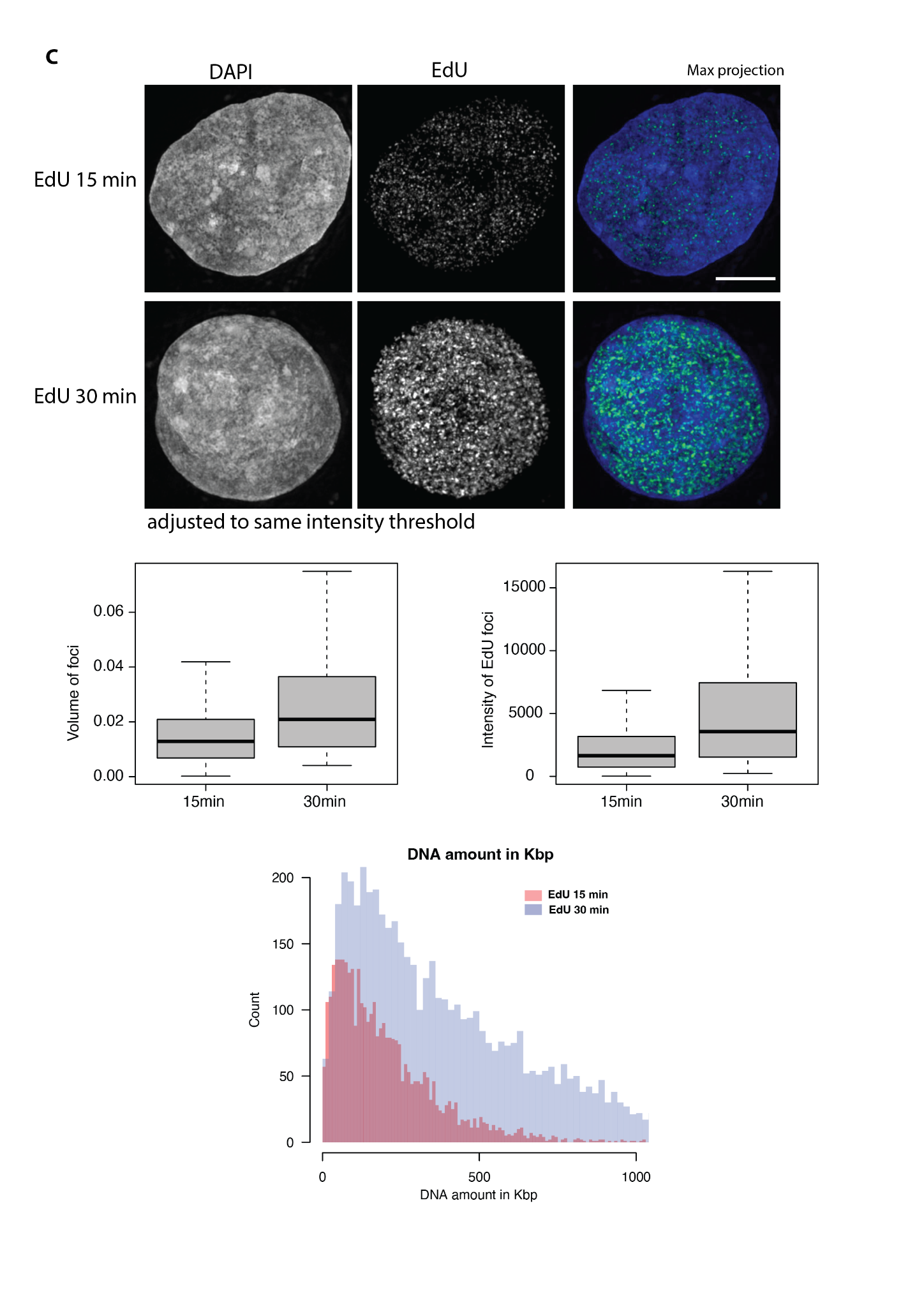
Supplementary figure 5: Analysis pipeline to quantify the DNA amount (in Kbp)

## (A) Step by step description of analysis pipeline for 3D SIM data (fixed cells) to quantify the DNA amount (in Kbp) within labeled chromatin in HeLa K GFP-PCNA cells (methods, DNA quantification). (B) Step by step description of analysis pipeline for 3D SIM data (fixed cells) to quantify the DNA amount (in Kbp) within labeled chromatin in HeLa K GFP-PCNA cells (methods, DNA quantification). (C) Volume (µm3), intensity of foci, DNA amount (in Kbp) quantification of HeLa K cells pulse labeled with EdU for 15 min and 30 min. Scale bar: 5 µm.


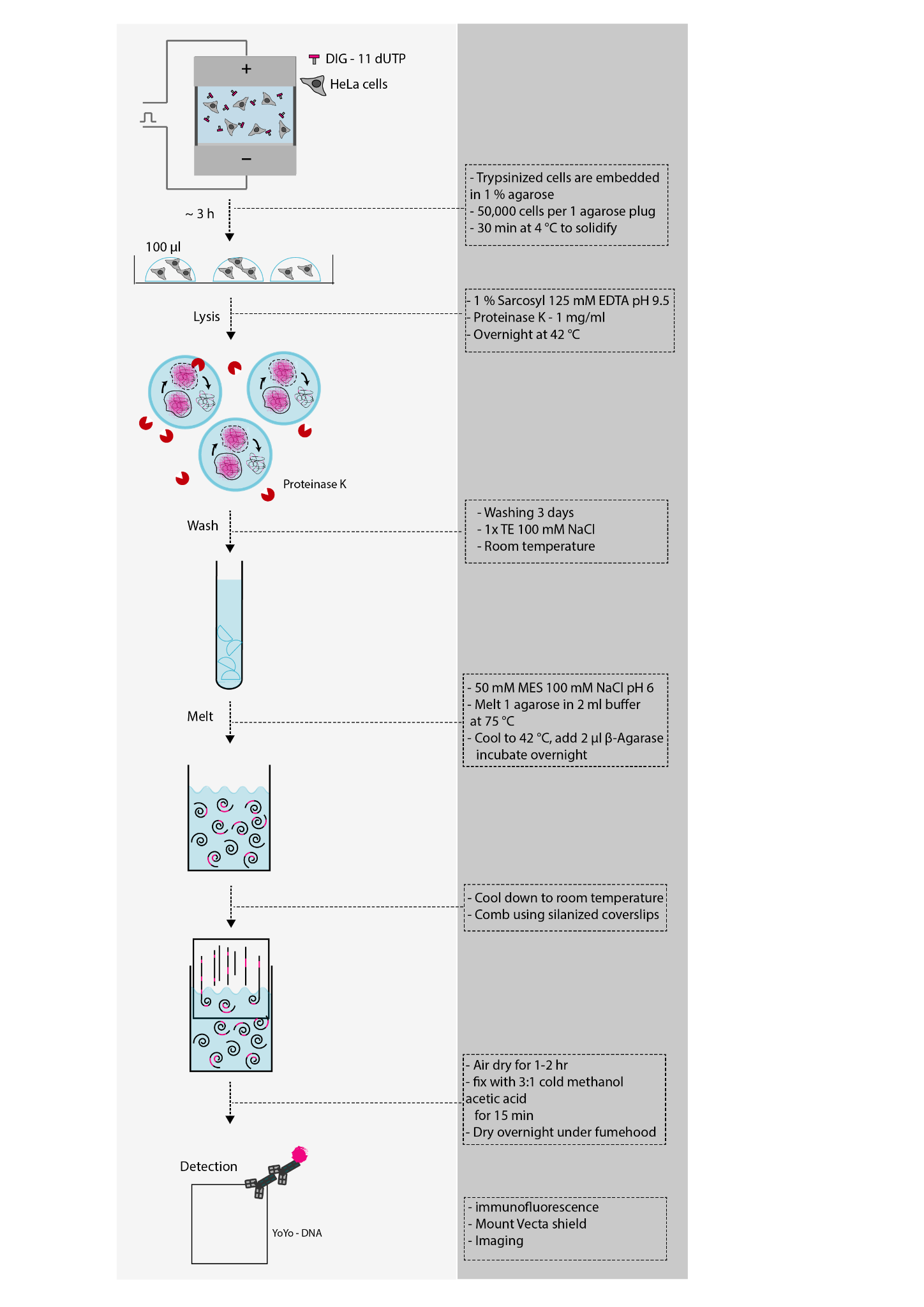


## Supplementary figure 6: Step by step protocol for preparation of DNA fibers for single molecule DNA fiber analysis (methods, DNA combing).


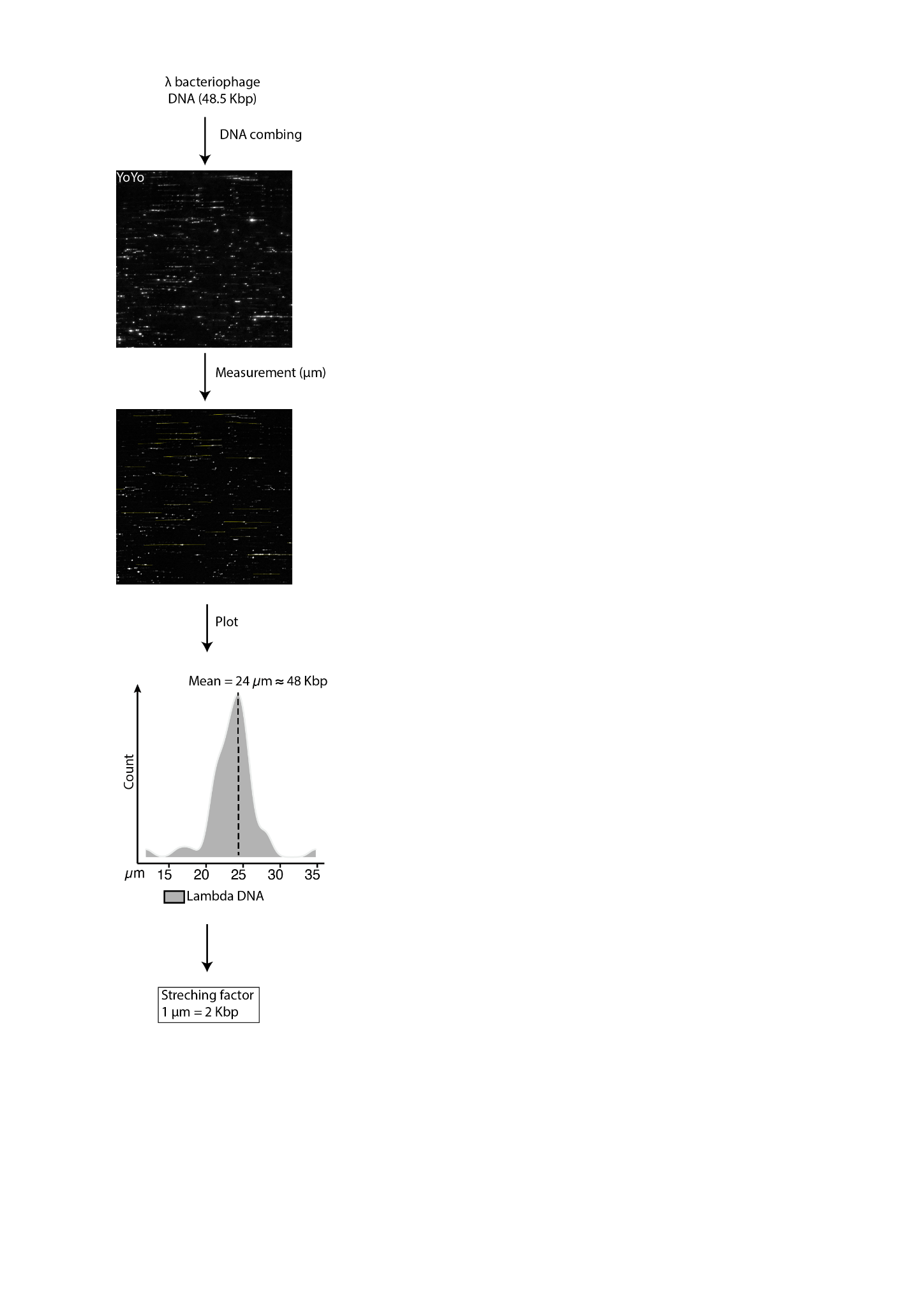


## Supplementary figure 7: DNA fiber combing of bacteriophage lambda DNA for calibration of stretching factor (methods, DNA combing).


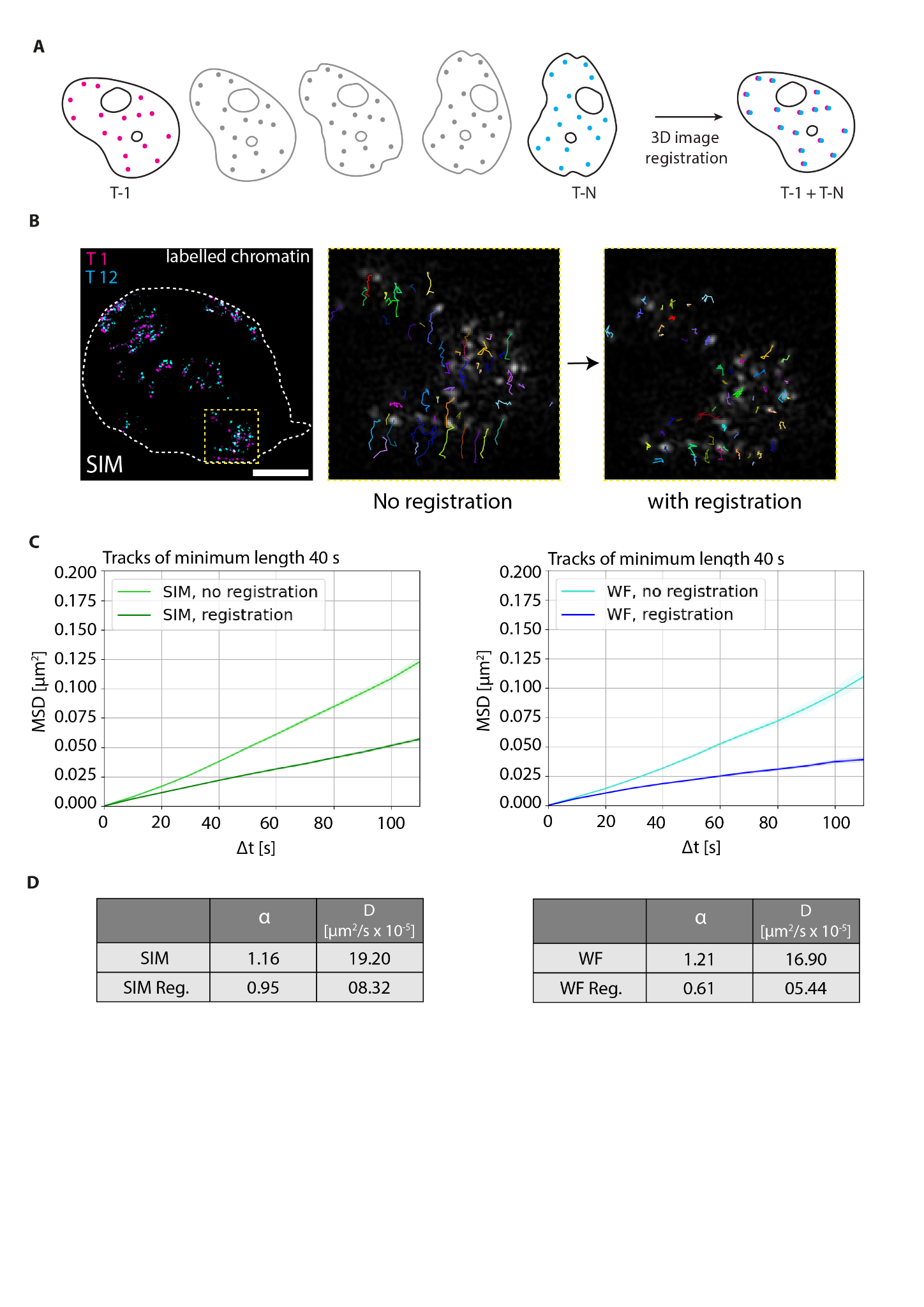


##

## Supplementary figure 8: 3D-registration of live cell datasets.

**(A)** Illustration of HeLa K GFP-PCNA live cells with labeled chromatin over time. The initial timepoint was used to perform 3D non-rigid registration of cells over time (methods, non-rigid registration). **(B)** PCNA (green) signal was used to identify the nuclear border (white dotted border). The labeled chromatin of time point 1 (T1, magenta) is overlaid on chromatin in time point 12 (T12, cyan). The chromatin tracks of the zoomed region (yellow insert) are shown with no registration and non-rigid registration. **(C)** The Mean Squared Displacement curves (MSD, µm^2^) over time intervals (s) for SIM (without registration, light green), SIM non-rigid registration (with registration, green), WF (without registration, light blue), WF non-rigid registration (with registration,dark blue) were then plotted. **(D)** The table details the values of the anomalous diffusion coefficient α and the diffusion coefficient D (µm^2^/s x 10^-5^). Scale bar: 5 µm.


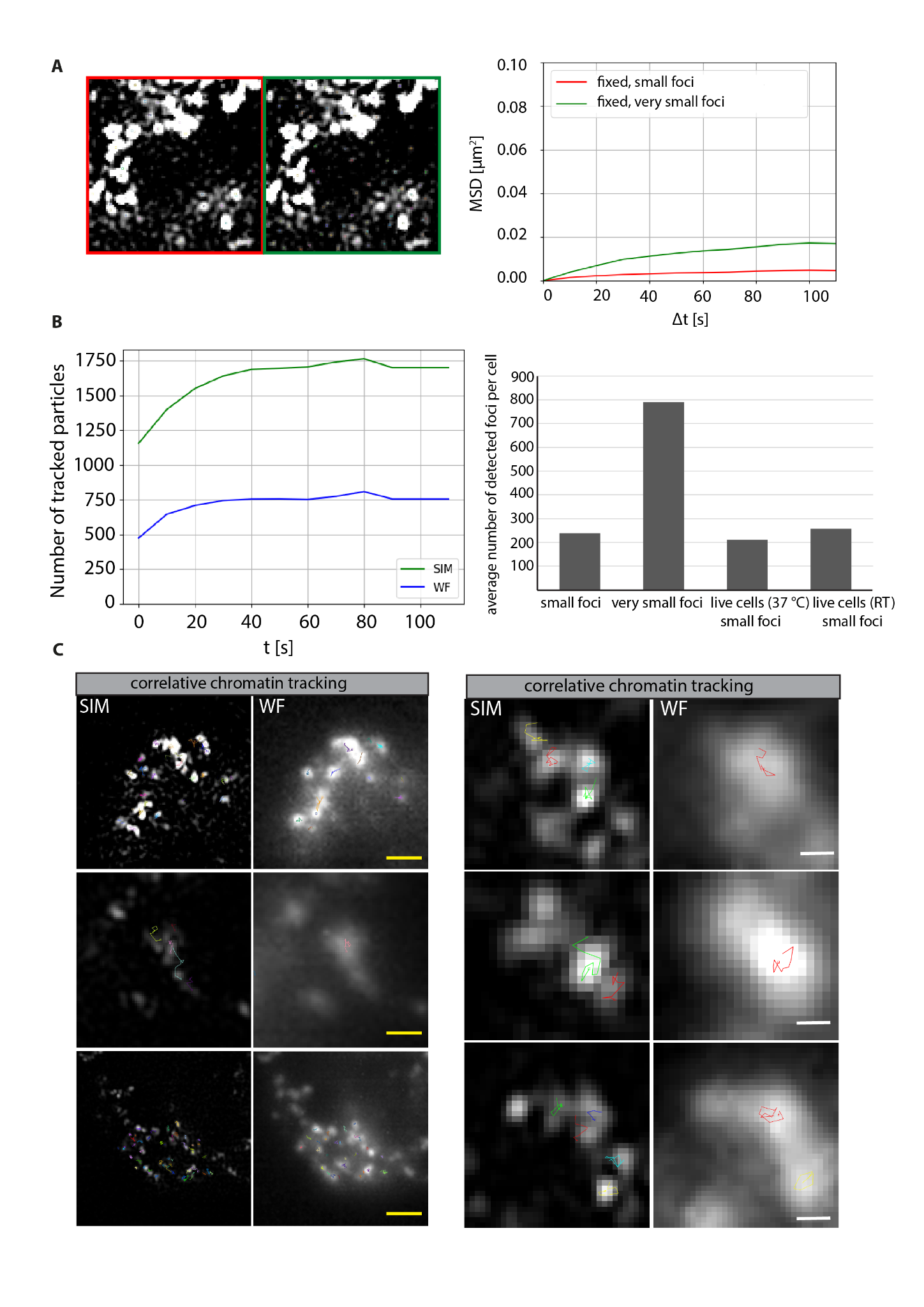


## Supplementary figure 9: Optimization of particle detection and comparison of particle detection at multiple resolutions.

**(A)** Representative image of fixed cell chromatin tracks with different strength of detection. Chromatin tracks of fixed cells with detection of small particles (red) and very small foci corresponding to fine structures (green) were used to obtain the Mean Squared Displacement (MSD, µm^2^) over time intervals (s) and plotted. Small and very small particles were determined by applying high and low thresholds on the LoG filter response, respectively. The high threshold was selected so that visually relevant foci are detected. **(B)** Comparison of the number of chromatin particles over time (s) in SIM and WF and average number of particles detected in fixed and live cells. **(C)** Representative images of particle detection and tracks of chromatin of same cells (regions) in SIM and WF microscopy. Scale bar: 1 µm (yellow) and 500 nm (white).

##


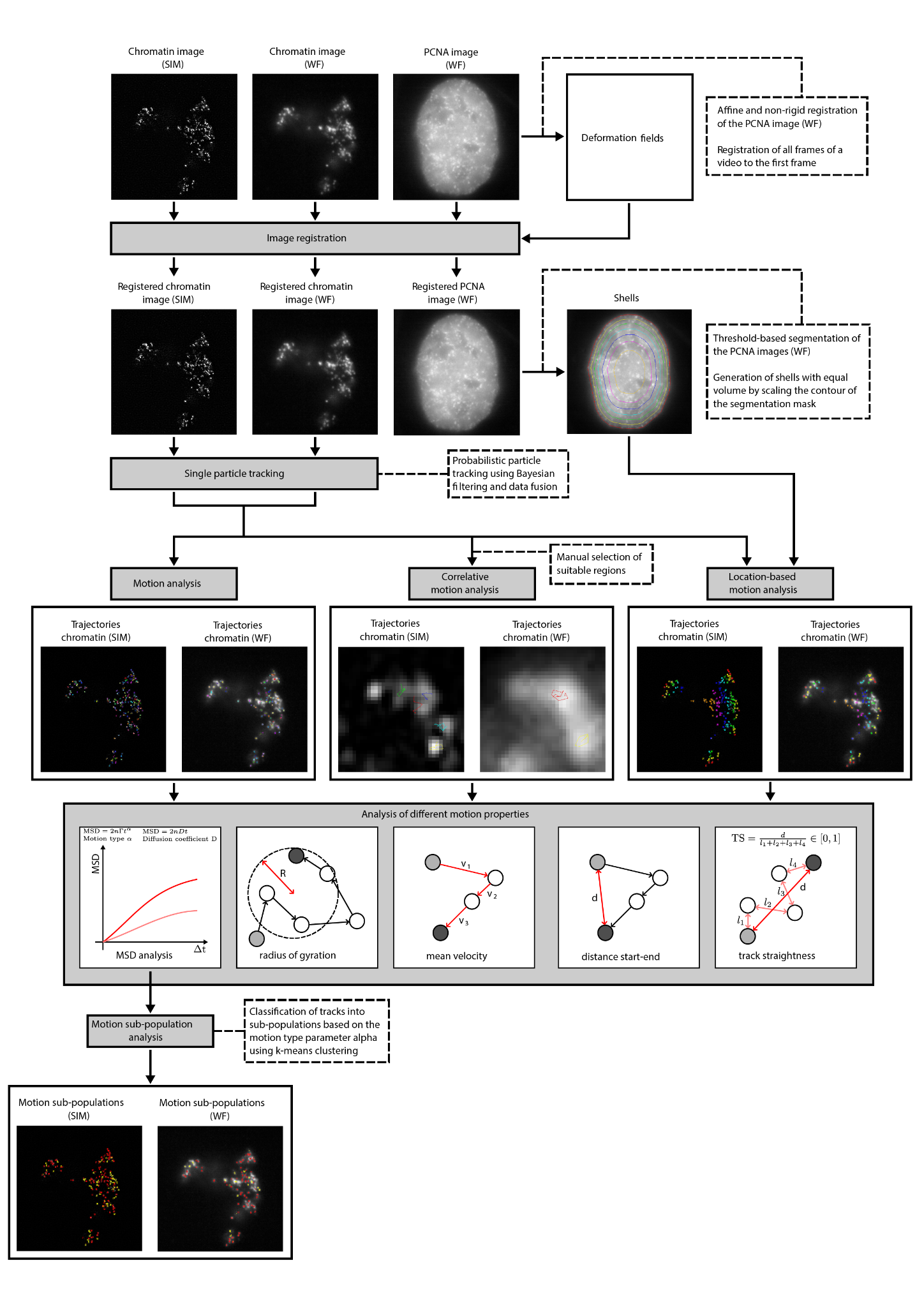


## Supplementary figure 10: Step by step pipeline description of chromatin tracking and motion analysis in 3D (x,y,z) for SIM and WF timelapse movies (methods, tracking).


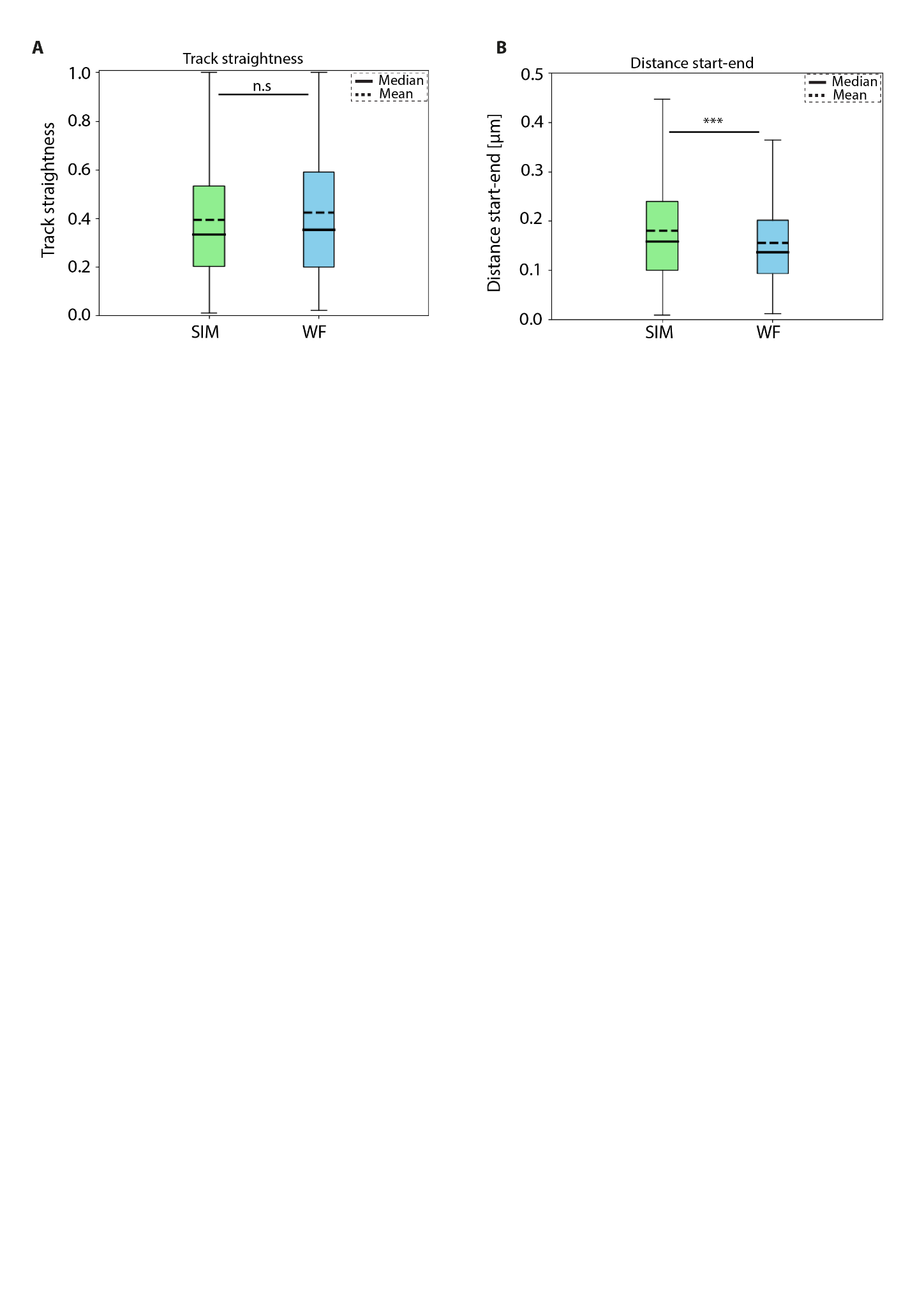


## Supplementary figure 11: Comparison of different parameters between SIM and WF chromatin tracks.

**(A)** Track straightness of labeled chromatin foci for SIM (green) and WF (blue) plotted as a box plot. **(B)** The distance start-end (µm) of labeled chromatin foci for SIM (green) and WF (blue) plotted as a box plot. The median and mean values of the measurements are indicated. The statistics of the plots are shown in the figure and listed in (Supplementary table 6).


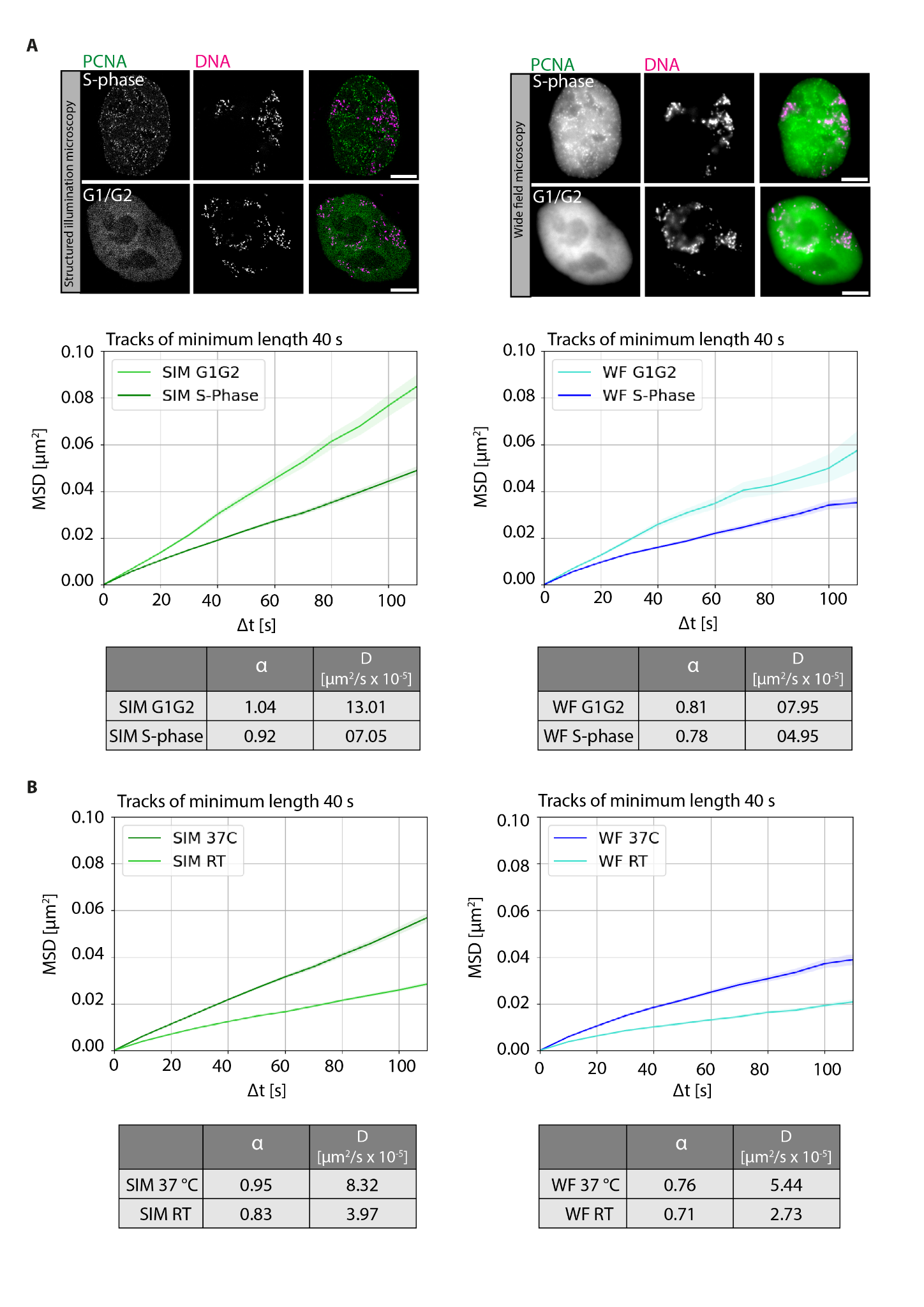


##
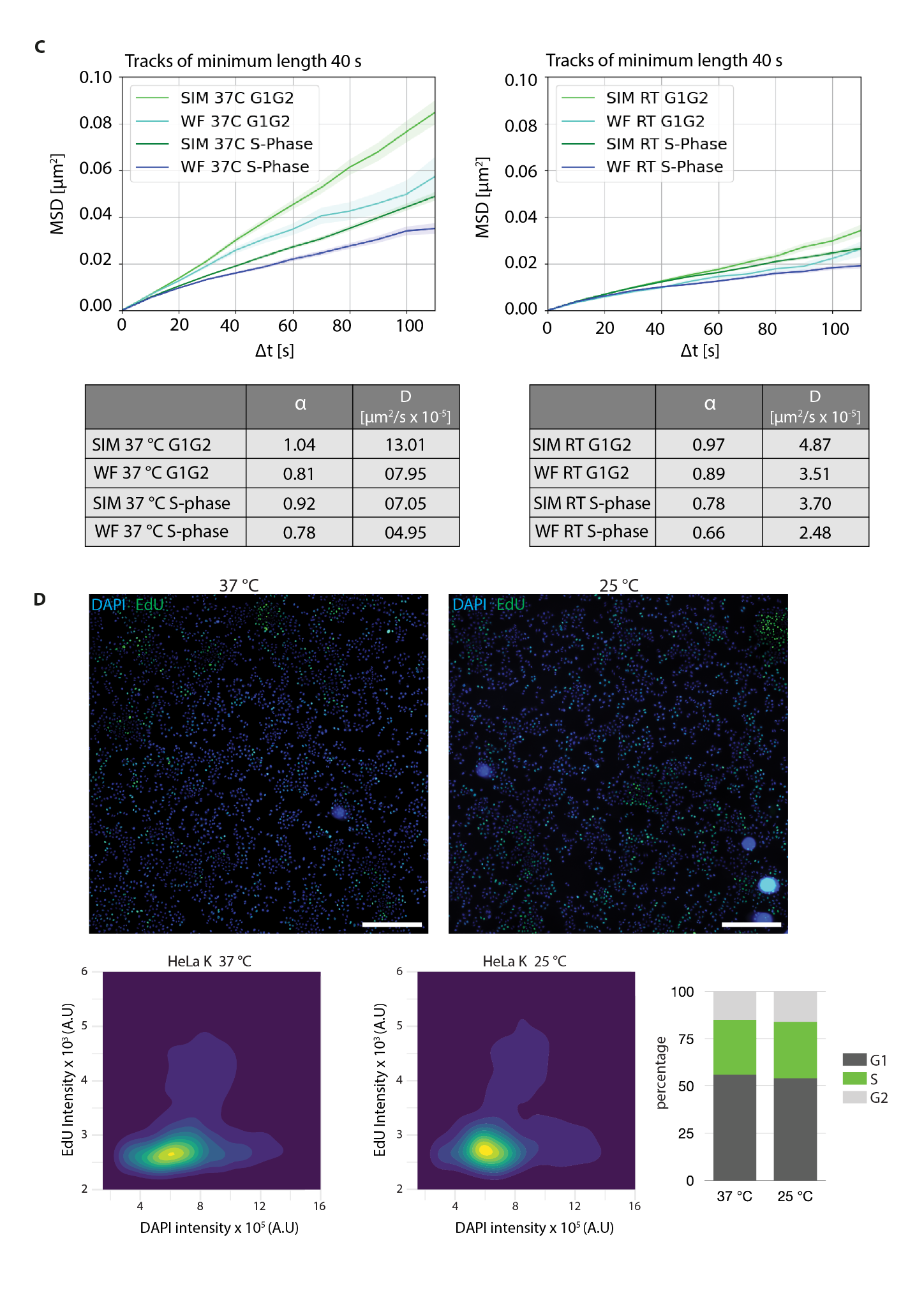
Supplementary figure 12: Effect of DNA replication and temperature on chromatin mobility.

**(A)** HeLa K cells with labeled DNA were used to obtain 3D live cell time lapse movies (frame interval of 10 sec) (Methods, Supplementary table 1, 2). Correlative imaging of two channels GFP-PCNA and labeled chromatin in WF and SIM were obtained (Supplementary table 4). During S-phase, proliferating cell nuclear antigen (PCNA) accumulates within the nucleus at sites of active DNA replication and exhibits a distinct puncta pattern. During G1 and G2, GFP-PCNA is diffusely distributed throughout the nucleus. GFP-PCNA patterns were used to classify cells in different cell cycle stages (Supplementary figure 3). The representative images show GFP-PCNA (green) and labeled DNA (magenta) for both SIM and WF resolutions. The registered time lapse movies were used to detect chromatin foci of both SIM and WF and then analyzed to obtain the Mean Squared Displacement curves (MSD, µm^2^) over time intervals (s) (Supplementary figure 8). The MSD curves over time intervals (s) were plotted for S-phase and G1/G2 for both SIM and WF. The table details the values of the anomalous diffusion coefficient α and the diffusion coefficient D (µm^2^/s x 10^-5^). Scale bar: 5 µm. **(B)** During live cell imaging of chromatin labeled HeLa K GFP-PCNA cells, experiments at two different temperatures (37 ℃ and room temperature (RT)) were performed. The MSD curves over time intervals (s) were plotted for imaging at 37 °C and RT for both SIM and WF. The table details the values of the anomalous diffusion coefficient α and the diffusion coefficient D (µm^2^/s x 10^-5^). (C) The MSD curves over time intervals (s) were plotted for S-phase and G1/G2 for RT and 37 ℃ for both SIM and WF. The table details the values of the anomalous diffusion coefficient α and the diffusion coefficient D (µm2/s x 10-5). (D) Representative images of fixed HeLa K cells labeled with EdU (green, 10 µM) for 15 minutes and DAPI (blue). Cell cycle profile analysis of HeLa K cells cultured at 37 ℃ and RT with 5% CO2. The boxplot shows the percentage of cells in G1, S, G2 for HeLa K cells cultured at 37 ℃ and RT. Scale bar: 200 µm.


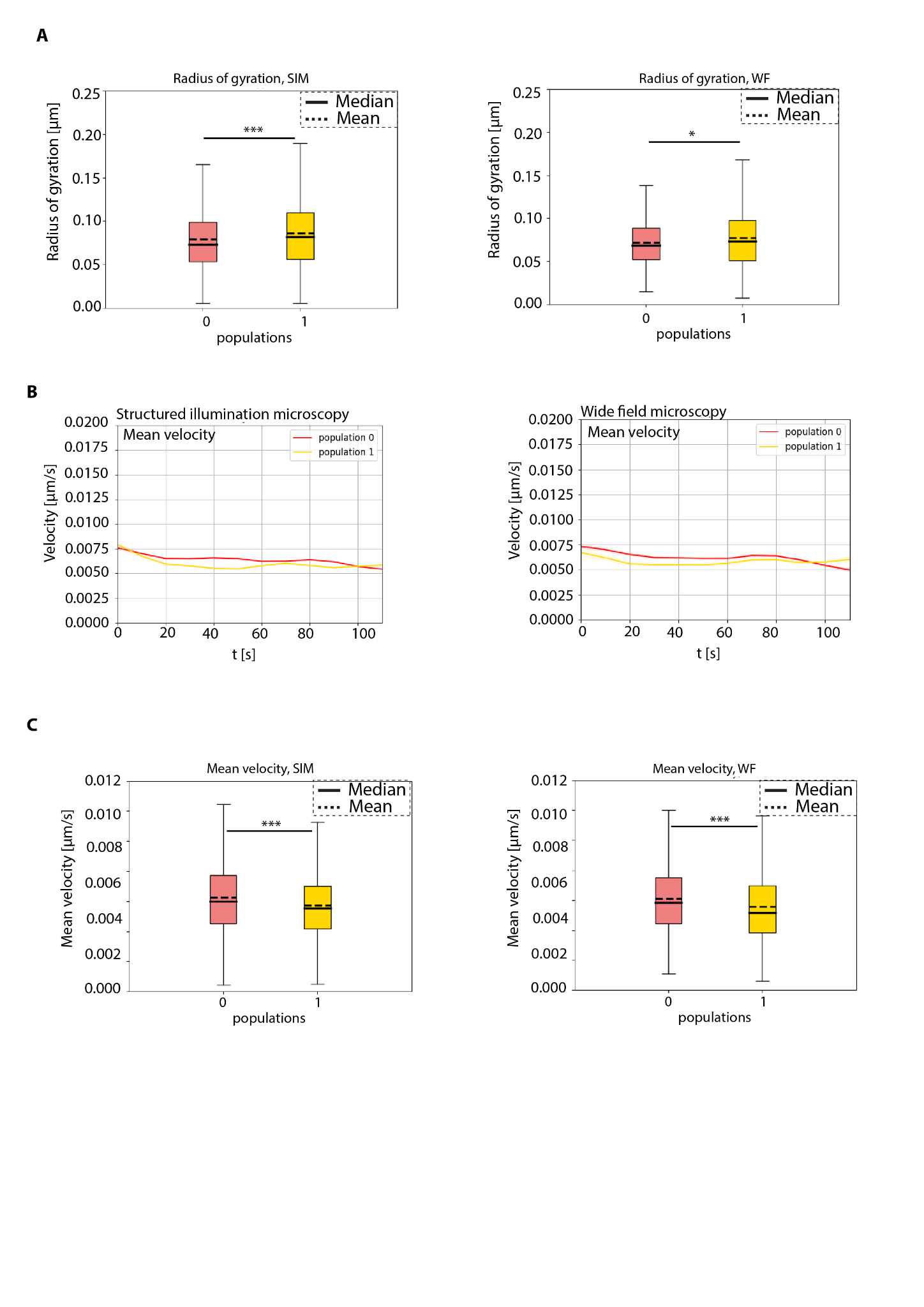


## Supplementary figure 13: Comparison of different parameters between SIM and WF chromatin tracks.

**(A)** The radius of gyration (µm) of labeled chromatin foci for SIM and WF time lapse videos for population 0 (red) and population 1 (yellow) plotted as a box plot (Figure 5). **(B)** Mean velocity (µm/s) of labeled chromatin foci for SIM and WF for population 0 (red) and population 1 (yellow) plotted as a curve over time (s). **(C)** The mean velocity (µm/s) of labeled chromatin foci for SIM and WF time lapses for population 0 (red) and population 1 (yellow) plotted as a box plot (Figure 5). The median and mean values of the measurements are indicated in the figure. The statistics of the plots are shown in the figure and listed in (Supplementary table 6).


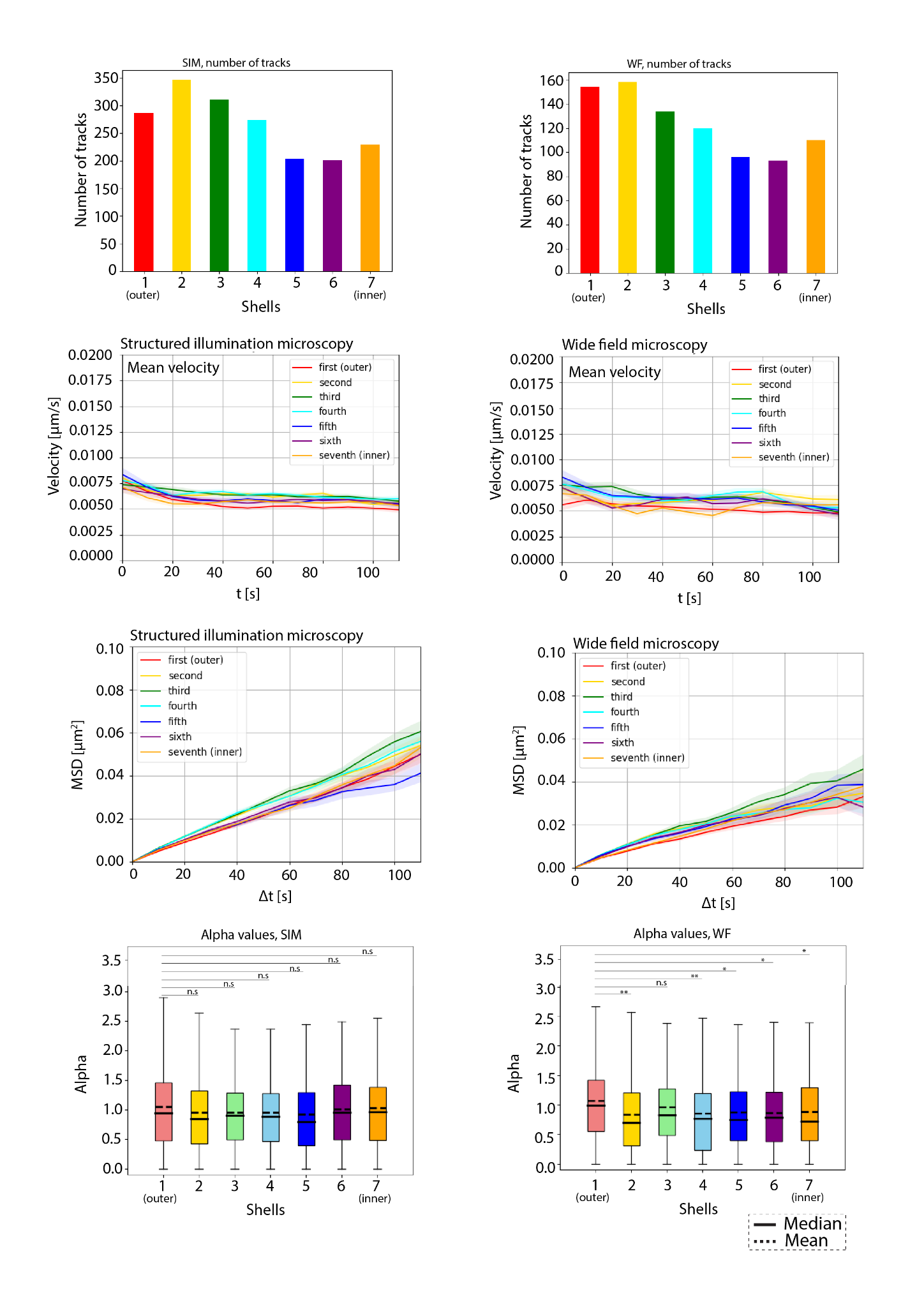


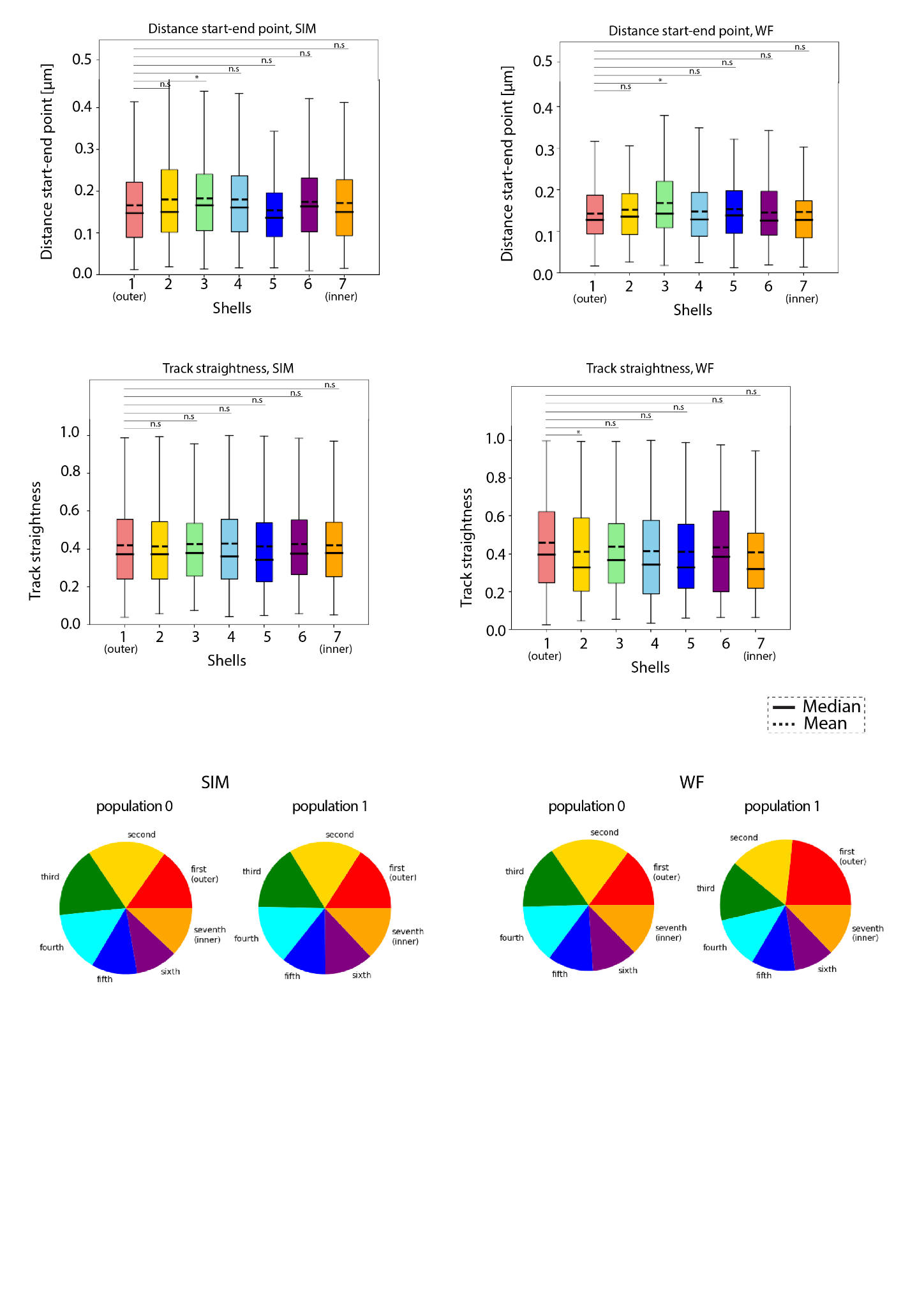


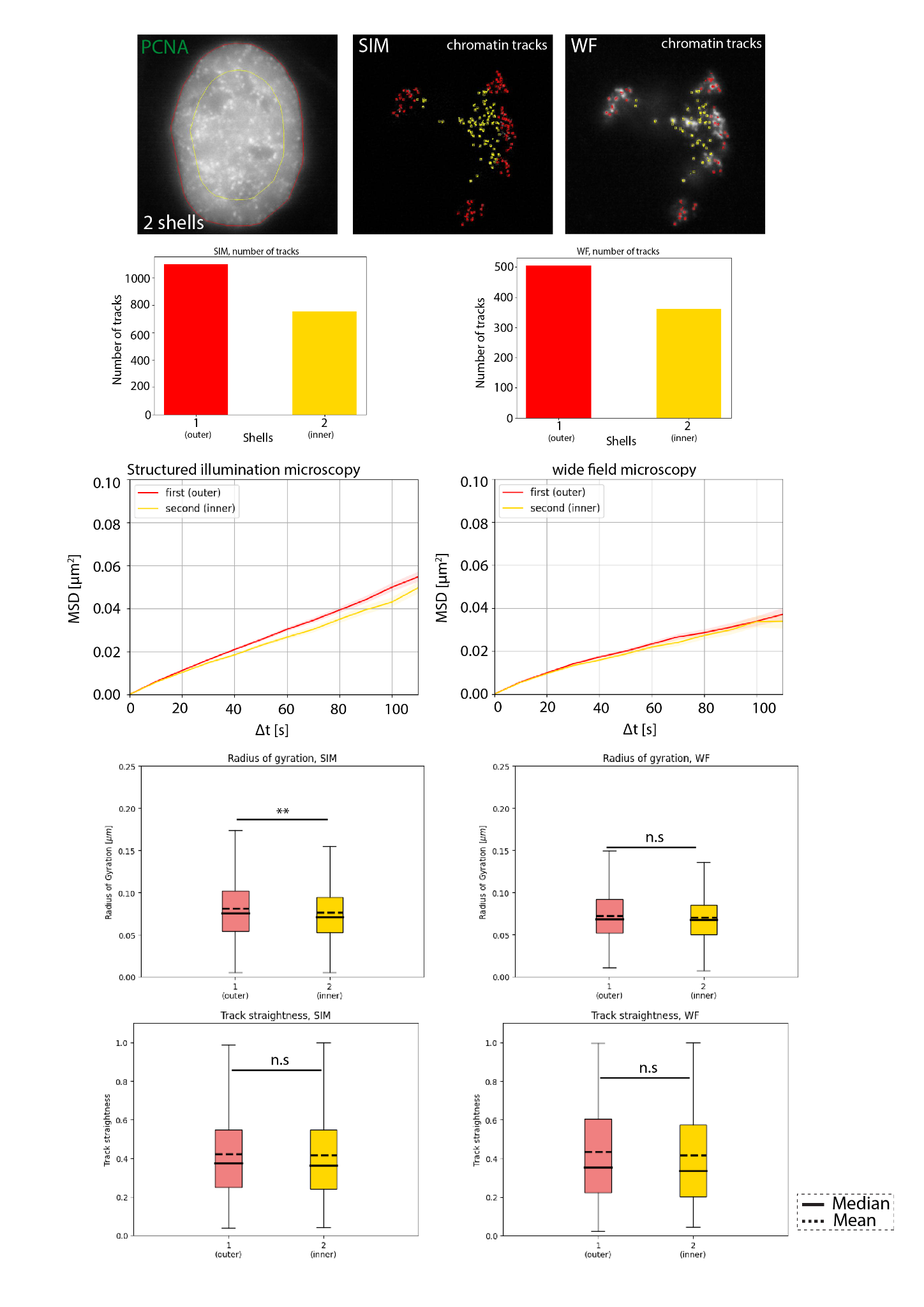


##
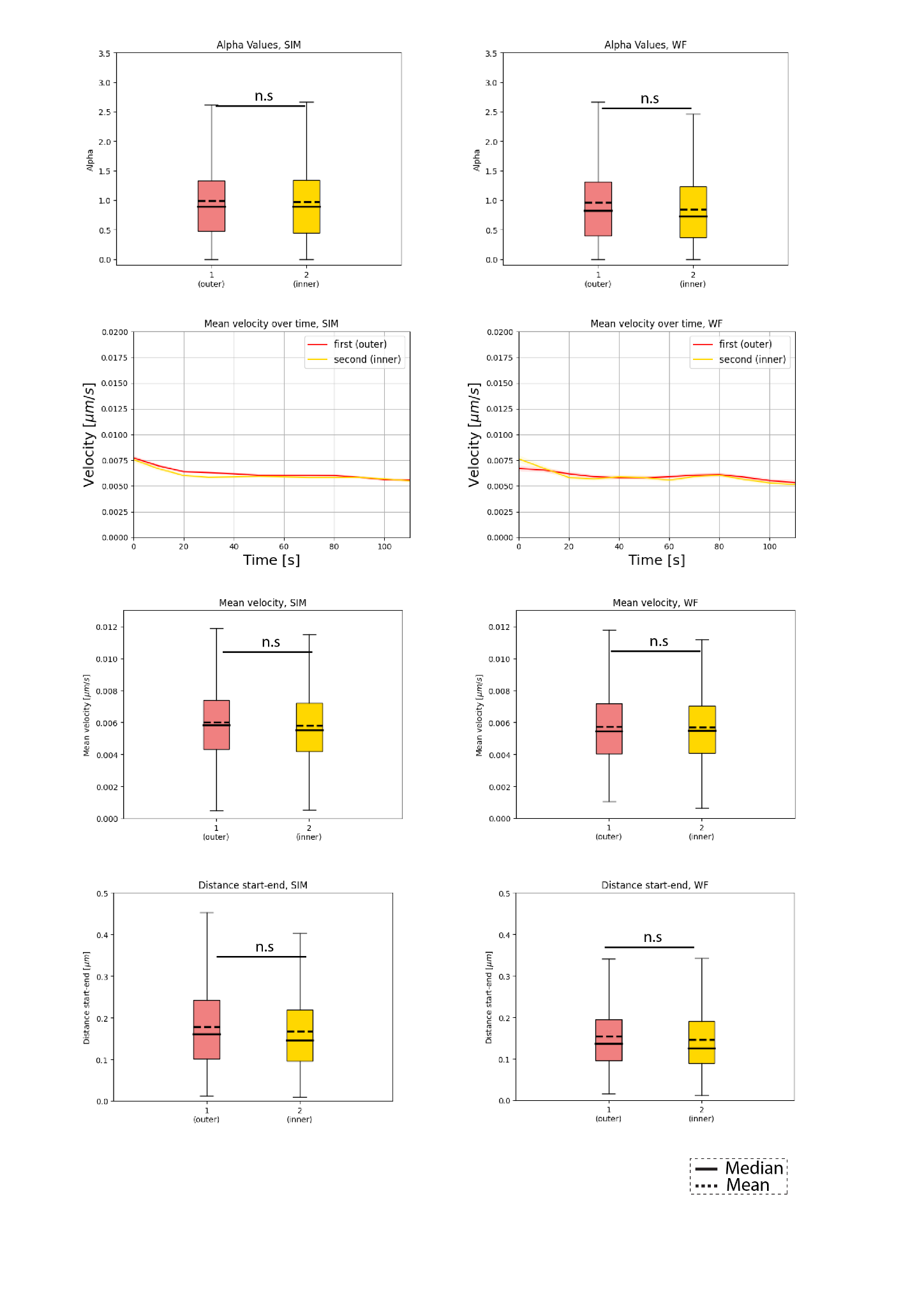


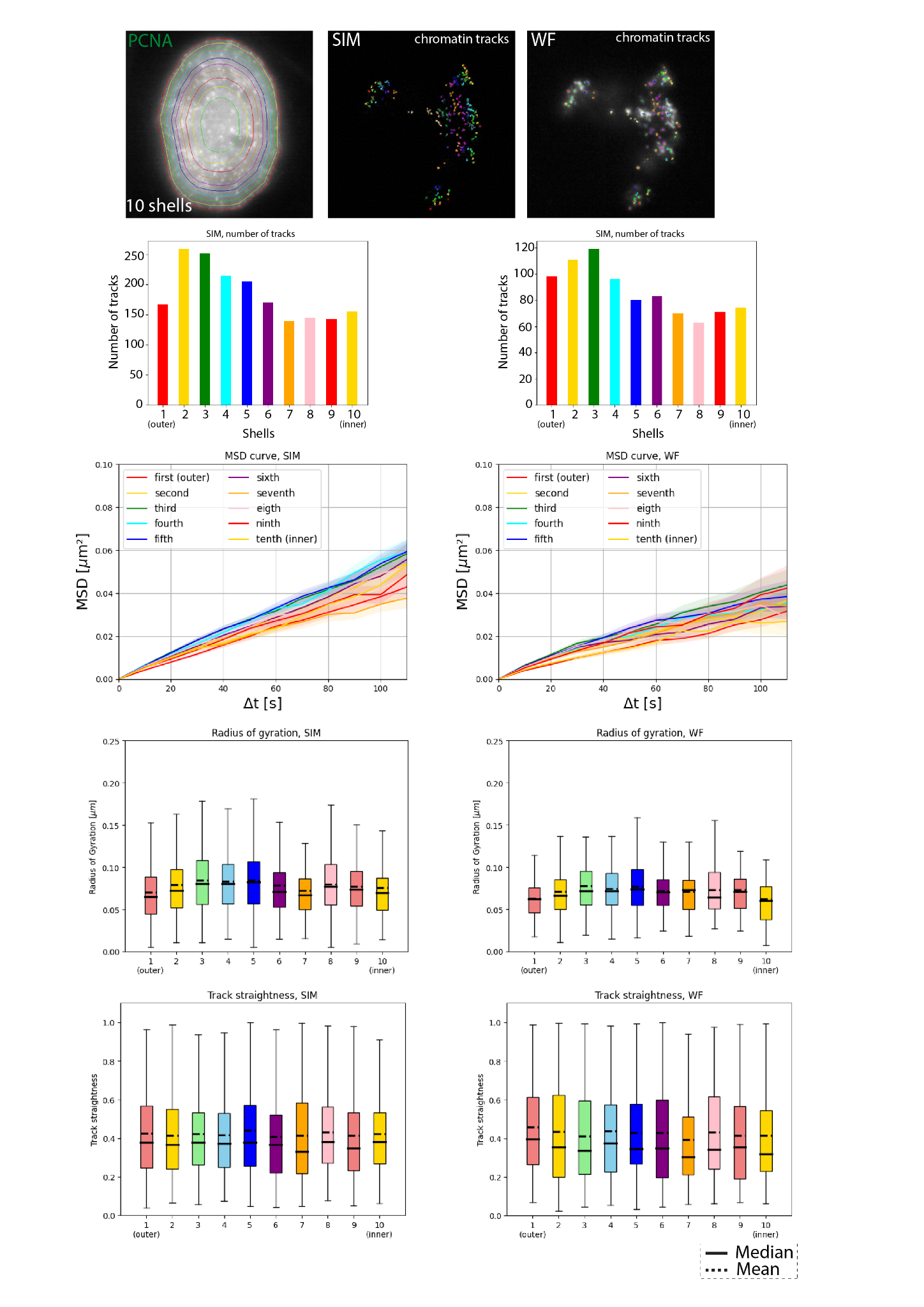


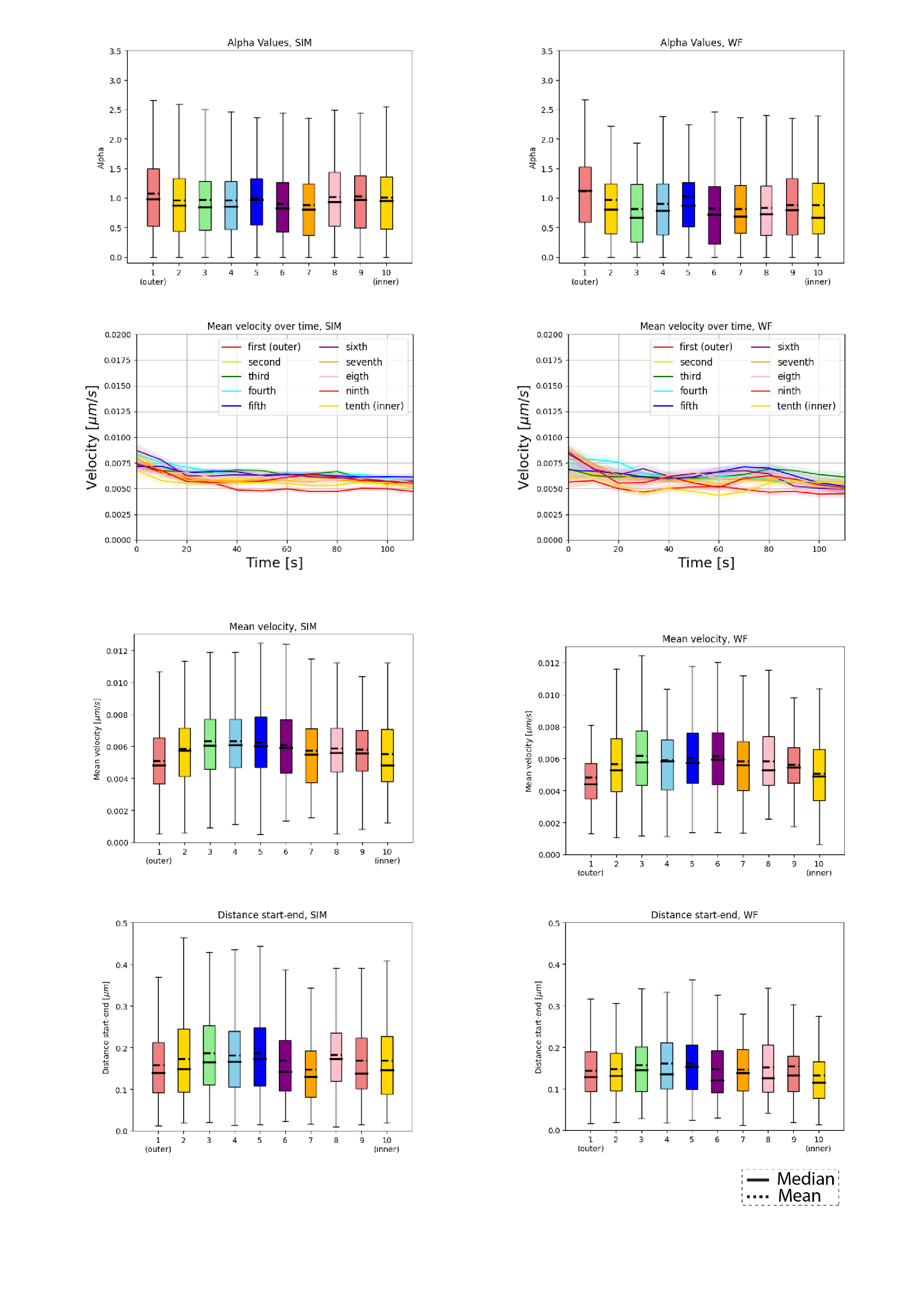


## Supplementary figure 14: Location-based analysis of chromatin domains with different shell numbers.

Location-based analysis of chromatin within the nucleus was performed using different numbers of shells (2, 7, 10) of equal volume. Chromatin was subdivided into different shells and color-coded according to the shell color. We computed and plotted different parameters within each shell such as the number of chromatin foci, mean velocity over time, Mean Square Displacement curves (MSD), radius of gyration, alpha values, distance start to end, and track straightness.


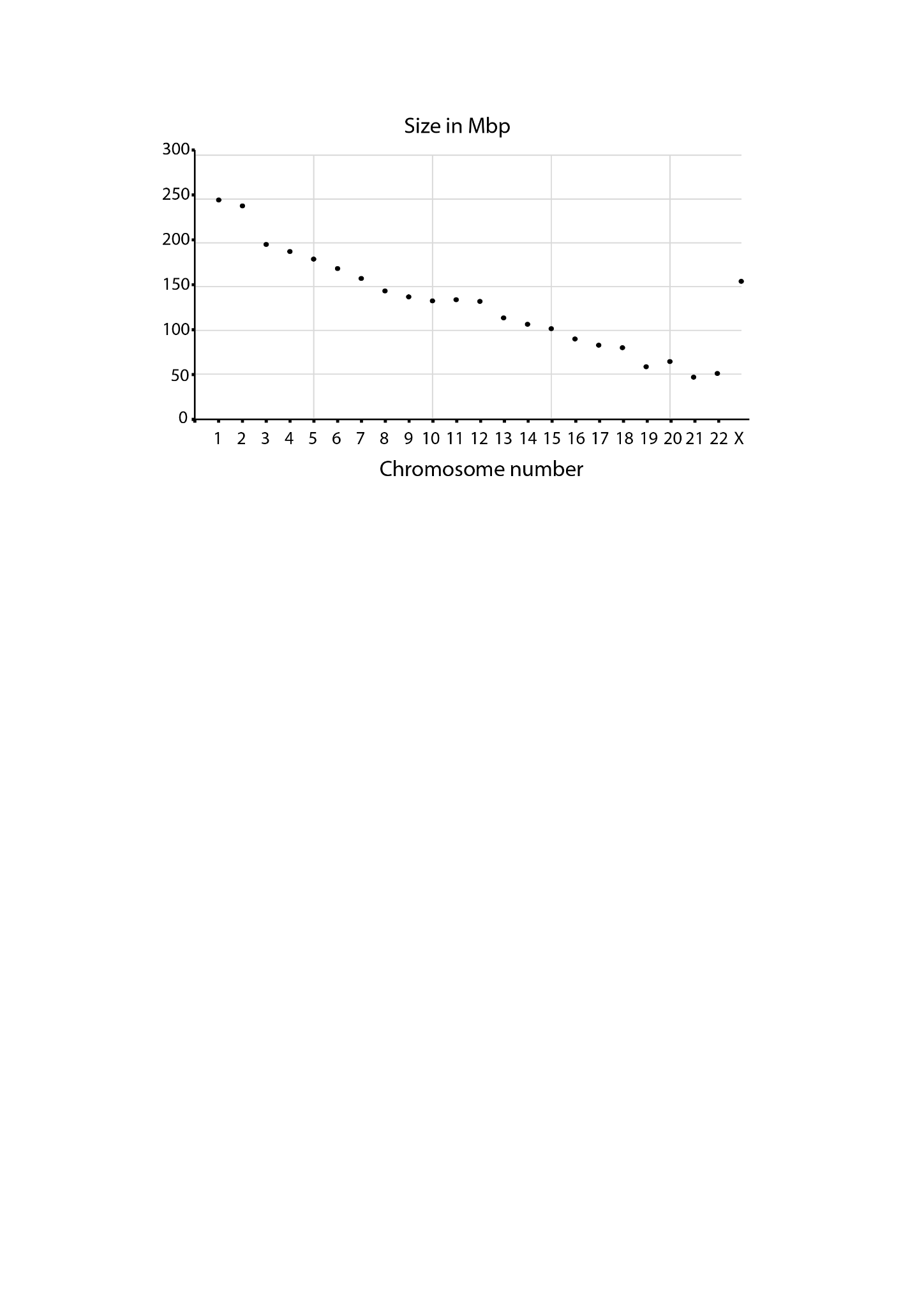


## Supplementary figure 15: Human chromosomes sizes.

#

#

#

#

#

#

#

#

# **SUPPLEMENTARY TABLES AND FIGURES**

## Supplementary table 1: Cell line characteristics

| **Name** | **Species** | **Type** | **Genome size**  **(Gbp)** | **Reference** |
| --- | --- | --- | --- | --- |
| HeLa K | Homo sapiens | Cervical adenocarcinoma | 9.682±0.002 | [(Erfle et al. 2007)](https://sciwheel.com/work/citation?ids=1001802&pre=&suf=&sa=0) |
| HeLa K GFP-PCNA | Homo sapiens | Cervical adenocarcinoma | 9.682±0.002 | [(Chagin et al. 2016)](https://sciwheel.com/work/citation?ids=2069726&pre=&suf=&sa=0) |
| HeLa K FRTLacZ | Homo sapiens | Cervical adenocarcinoma | 9.682±0.002 | [(Chagin et al. 2016)](https://sciwheel.com/work/citation?ids=2069726&pre=&suf=&sa=0) |

## Supplementary table 2: Nucleotide and chemical characteristics

| **Name** | **Application** | **Detection** | **Cat #** | **Company** |
| --- | --- | --- | --- | --- |
| ATTO590-dUTP | Replication labeling  (Labeling of nascent DNA) | - | NU-821-590 | Jena Biosciences, Germany |
| Cy3-dUTP | Replication labeling  (Labeling of nascent DNA) | - | ABD-17025 | Biomol GmbH,  Germany |
| 5-ethynyl-2’-  deoxyuridine (EdU) | Labeling of nascent DNA  in pulse (chase) experiments | ClickIT chemistry | 7773.1 | Carl Roth, Germany |
| 5-Bromo-2’-  Deoxyuridine  (BrdU) | Labeling of nascent DNA  in pulse (chase) experiments | Antibody | B5002 | Sigma Aldrich,  Merck, Germany |
| DIG-11-dUTP | DNA fibers | Antibody | NU-803-DIGXS | Jena Biosciences,  Germany |

## Supplementary table 3: Primary and secondary antibody characteristics

| **Reactivity** | **Host** | **Clonality** | **Dilution** | **Application** | **Cat / Clone^#^** | **Company / References** |
| --- | --- | --- | --- | --- | --- | --- |
| Anti BrdU* | rabbit | Monoclonal | 1:500 | IF | 600-401-C29 | Rockland, USA |
| anti digoxigenin | rabbit | Monoclonal | 1:500 | IF | #9H27L19 | Thermo fisher scientific, USA |
| anti-rabbit  IgG Cy3 | Donkey | Polyclonal | 1:500 | IF (fluorescent  secondary) | JIM-711-165-152 | Jackson immuno research, USA |
| anti-rabbit  IgG Cy5 | Donkey | Polyclonal | 1:500 | IF (fluorescent  secondary) | 711-175-152 | Jackson immuno research, USA |

*****DNAse treatment #Clone number

## Supplementary table 4: Imaging systems characteristics

| **Microscope/**  **Company** | **Lasers/lamps** | **Filters (ex. &**  **em. [nm])*** | **Objectives/**  **lenses** | **Detection**  **system** | **Incubation**  **system** | **Application** |
| --- | --- | --- | --- | --- | --- | --- |
| DeltaVision OMX V3 Blaze system (Applied Precision) | 592 nm and 488 nm diode lasers | Ex (464/492 nm)  Em (500/523 nm)  Ex (531/556 nm)  Em (564/611 nm) | 60/1.42 NA PlanApo oil objective | sCMOS cameras (PCO) | 37°C incubation chamber, with 5% C02 and 60% humidity | 3D-SIM and 3D WF live cell time lapse microscopy |
| Nikon TiE2 inverted with crest spinning disk unit/ Nikon, Japan | SPECTRA X light engine  395/25 nm with 295 mW  440/20 nm with 256 mW  470/24 nm with 196 mW  510/25 nm with 62 mW  540/30 nm with 231 mW  550/15 nm with 260 mW  575/25 nm with 310 mW | LED-DA/FI/TR/Cy5-4X-B  Quadbandpassex:390/18,  475/35,  535/50  em:460/60,  530/43,  580LP | 40x air (0.95 NA) & 250 µm WD*** | Cooled Nikon Qi2 camera and 16.25 megapixel sCMOS sensor. readout noise is: 2.2. electron | Self build - 37°C incubation chamber, with 5% C02 and 60% humidity chamber | high throughput, high content imaging and image analysis |
| Bio Rad S3 cell sorter | 488 nm with 100 mW/ 561 nm with 100 mW | The filter sets are:  FL1: 525/30 nm  FL2: 587/25 nm  FL3: 615/25 nm  FL4: 655 nm LP | – | Forward scatter (FSC) with PMT  Side scatter (SSC) with PMT  2 fluorescence detectors with PMT  Minimum resolution: 0.5 µm | 4–37°C  Peltier solid-state system | Sorting of cells based on intensity |

* ex.: excitation & em.: emission, ** dichroic specification, *** WD: working distance.

## Supplementary Table 5: Software

| **Name** | **Version** | **Website** | **Company/University** | **Application** |
| --- | --- | --- | --- | --- |
| Volocity | 6.3 | - | PerkinElmer, USA | Analysis of DNA domain sizes |
| Fiji | 1.53c | <https://imagej.net/software/fiji/> | Wayne Rasband, National Institutes of Health, USA | Image processing and image analysis |
| RStudio | 1.1.447-1.2.5033 | <https://rstudio.com/> | RStudio | Statistical analysis and plotting |
| FCS express | RUO 7.20.0020 | <https://denovosoftware.com> | Denovo software by dotmatics | For analysis of flow cytometry data and plots |
| Adobe Illustrator | 2024 | <https://www.adobe.com/> | Adobe, USA | Graphical sketch and figures arrangement |

##

## Supplementary Table 6: Statistics

| **Figure** | **Sample** | **n/number of replicates** | **Total percentage** | **Percentage of overlap** | **P value** |
| --- | --- | --- | --- | --- | --- |
| **1B** | *HeLa K nucleotide overlap*  0’ chase  15’ chase  30’ chase  45’ chase  60’ chase  120’ chase | *3 biological*  *replicates* | 100% | 56 %  33 %  10 %  8 %  7 %  10 % | NA |
| **Figure** | **Sample** | **Number of cells** | **Median (Kbp)** | **Mode (Kbp)** | **P value** |
| **2A** | SIM datasets | 30 | 110 | 70 | NA |
|  | WF datasets | 30 | 210 | 70 | NA |
| **Figure** | **Sample** | **Number of tracks** | **Alpha (ɑ)** | **Diffusion coefficient (D, µm^2^/s x 10^-5^)** | **Gamma (Γ, µm^2^/s^ɑ^ x 10^-5^)** |
| **3C** | SIM | 2113 | 0.95 | 8.32 | 10.72 |
|  | WF | 1000 | 0.76 | 5.44 | 18.18 |
|  | Fixed | 1903 | 0.46 | 0.52 | 09.49 |
| **Figure** | **Sample** | **Number of tracks** | **Median (µm)** | **Mean (µm)** | **P value** |
| **3E** | Radius of gyration, SIM | 2113 | 0.0763 | 0.0818 | 0.0000006 |
|  | Radius of gyration, WF | 1000 | 0.0699 | 0.0739 | 0.0000006 |
| **Figure** | **Sample** | **Number of tracks** | **Median (µm^3^)** | **Mean (µm^3^)** | **P value** |
| **3F** | Mean particle size, SIM | 2113 | 0.0087 | 0.0127 | 0.0000 |
|  | Mean particle size, WF | 1000 | 0.0112 | 0.0256 | 0.0000 |
| **Figure** | **Sample** | **Number of tracks** | **Median (µm/s)** | **Mean (µm/s)** | **P value** |
| **3H** | Mean velocity, SIM | 2113 | 0.00585 | 0.00604 | 0.098254 |
|  | Mean velocity, WF | 1000 | 0.00556 | 0.00590 | 0.098254 |
| **Figure** | **Sample** | **Number of tracks** | **Alpha (ɑ)** | **Diffusion coefficient (D, µm^2^/s x 10^-5^)** | **Gamma (Γ, µm^2^/s^ɑ^ x 10^-5^)** |
| **4B, 4C** | G1/G2, SIM | 519 | 1.04 | 13.01 | 10.59 |
|  | G1/G2, WF | 257 | 0.81 | 07.95 | 20.79 |
|  | S-phase, SIM | 1594 | 0.92 | 07.05 | 10.45 |
|  | S-Phase, WF | 743 | 0.78 | 04.95 | 14.38 |
| **4E, 4F** | 37 ℃, SIM | 2113 | 0.95 | 08.32 | 10.72 |
|  | 37 ℃, WF | 2568 | 0.76 | 05.44 | 18.18 |
|  | RT, SIM | 1000 | 0.83 | 03.97 | 09.54 |
|  | RT, WF | 1104 | 0.71 | 02.73 | 11.88 |
| **Figure** | **Sample** | **Number of tracks** | **Median (µm)** | **Mean (µm)** | **P value** |
| **5B** | Population 0, SIM | 1172 | 1.8429 | 2.3067 | 0.560174 |
|  | Population 1, SIM | 678 | 1.9281 | 2.3652 | 0.560174 |
|  | Population 0, WF | 560 | 1.8756 | 2.3504 | 0.142176 |
|  | Population 1, WF | 305 | 1.7083 | 2.2399 | 0.142176 |
| **Figure** | **Sample** | **Number of tracks** | **Median (µm^3^)** | **Mean (µm^3^)** | **P value** |
| **5C** | Particle size, SIM  Population 0  Population 1 | 1308 805 | 0.0087 0.0088 | 0.0128 0.0125 | 0.959039 |
|  | Particle size, WF  Population 0  Population 1 | 619 381 | 0.0121 0.0107 | 0.0241 0.0282 | 0.328000 |
| **Figure** | **Sample** | **Number of tracks** | **Median** | **Mean** | **P value** |
| **5D** | Track straightness, SIM  Population 0  Population 1 | 1308 805 | 0.2874 0.5359 | 0.3310 0.5816 | 0.00000 |
|  | Track straightness, WF  Population 0  Population 1 | 619 381 | 0.2596 0.5842 | 0.3181 0.6252 | 0.00000 |
| **Figure** | **Sample** | **Number of tracks** | **Median (µm)** | **Mean (µm)** | **P value** |
| **5E** | Distance start-end, SIM  Population 0  Population 1 | 1308  805 | 0.1308 0.2227 | 0.1463 0.2339 | 0.000000 |
|  | Distance start-end, WF  Population 0  Population 1 | 619 381 | 0.1139 0.1934 | 0.1259 0.2047 | 0.000000 |
| **Figure** | **Sample** | **Sample** | **Median (µm/s)** | **Mean (µm/s)** | **P value (shell 1 to shell n)** |
| **6B** | Mean velocity, SIM | Shell 1 (outer) | 0.005100 | 0.005370 | NA |
|  |  | Shell 2 | 0.005982 | 0.006184 | 0.00003471 |
|  |  | Shell 3 | 0.006042 | 0.006252 | 0.00000440 |
|  |  | Shell 4 | 0.006122 | 0.006257 | 0.00000867 |
|  |  | Shell 5 | 0.005642 | 0.005824 | 0.05842831 |
|  |  | Shell 6 | 0.005344 | 0.005750 | 0.06446484 |
|  |  | Shell 7 (inner) | 0.005279 | 0.005687 | 0.18390360 |
|  | Mean velocity, WF | Shell 1 (outer) | 0.004692 | 0.005072 | NA |
|  |  | Shell 2 | 0.005620 | 0.006012 | 0.00023403 |
|  |  | Shell 3 | 0.005924 | 0.005985 | 0.00042127 |
|  |  | Shell 4 | 0.005820 | 0.006169 | 0.00012767 |
|  |  | Shell 5 | 0.005649 | 0.005944 | 0.00250355 |
|  |  | Shell 6 | 0.005220 | 0.005655 | 0.02411346 |
|  |  | Shell 7 (inner) | 0.005332 | 0.005265 | 0.31939619 |
| **Figure** | **Sample** | **Sample** | **Median (µm)** | **Mean (µm)** | **P value (shell 1 to shell n)** |
| **6C** | Radius of gyration, SIM | Shell 1 (outer) | 0.06907 | 0.07462 | NA |
|  |  | Shell 2 | 0.07400 | 0.08233 | 0.00531 |
|  |  | Shell 3 | 0.08140 | 0.08370 | 0.00037 |
|  |  | Shell 4 | 0.07905 | 0.08278 | 0.00300 |
|  |  | Shell 5 | 0.06826 | 0.07356 | 0.84909 |
|  |  | Shell 6 | 0.07356 | 0.07787 | 0.11634 |
|  |  | Shell 7 (inner) | 0.07295 | 0.07730 | 0.25357 |
|  | Radius of gyration, WF | Shell 1 (outer) | 0.06374 | 0.06544 | NA |
|  |  | Shell 2 | 0.06950 | 0.07477 | 0.00701 |
|  |  | Shell 3 | 0.07411 | 0.07686 | 0.00072 |
|  |  | Shell 4 | 0.07011 | 0.07390 | 0.01274 |
|  |  | Shell 5 | 0.07324 | 0.07253 | 0.05828 |
|  |  | Shell 6 | 0.06490 | 0.07055 | 0.27735 |
|  |  | Shell 7 (inner) | 0.06580 | 0.06756 | 0.90629 |
| **Figure** | **Sample** | **Percentage of population 0** | **Percentage of population 1** | **Ratio population0/**  **population 1** | **–** |
| **6D/ Supplementary figure 14** | Shell 1 (outer), SIM | 15 % | 16 % | 0.93 | – |
|  | Shell 2, SIM | 19 % | 18 % | 1.05 | – |
|  | Shell 3, SIM | 17 % | 16 % | 1.06 | – |
|  | Shell 4, SIM | 15 % | 15 % | 1 | – |
|  | Shell 5, SIM | 11 % | 11 % | 1 | – |
|  | Shell 6, SIM | 10 % | 12 % | 0.83 | – |
|  | Shell 7 (inner), SIM | 12 % | 13 % | 0.92 | – |
|  | Shell 1 (outer), WF | 15 % | 23 % | 0.65 | – |
|  | Shell 2, WF | 20 % | 16 % | 1.25 | – |
|  | Shell 3, WF | 16 % | 15 % | 1.06 | – |
|  | Shell 4, WF | 14 % | 13 % | 1.07 | – |
|  | Shell 5, WF | 11 % | 11 % | 1 | – |
|  | Shell 6, WF | 11 % | 10 % | 1.1 | – |
|  | Shell 7 (inner), WF | 13 % | 23 % | 0.56 | – |
| **Figure** | **Sample** | **Number of tracks** | **Alpha (ɑ)** | **Diffusion coefficient (D, µm^2^/s x 10^-5^)** | **Gamma (Γ, µm^2^/s^ɑ^ x 10^-5^)** |
| **7B, 7C** | SIM | 2113 | 0.95 | 08.32 | 10.72 |
|  | WF | 1000 | 0.76 | 05.44 | 18.18 |
|  | Territories | 48 | 1.06 | 03.18 | 02.45 |
| **Supp Figure** | **Sample** | **Cell cycle stage** | **Mode (DNA intensity)** | **Cell cycle correction factor (C)** | **–** |
| **3** | HeLa K GFP-PCNA cells asynchronous population propidium iodide stained | G1 | 16480 | 1.00 | – |
|  |  | Early S-phase | 17550 | 1.06 | – |
|  |  | Mid S-phase | 21050 | 1.27 | – |
|  |  | Late S-phase | 28100 | 1.71 | – |
|  |  | G2 | 32644 | 1.98 | – |
| **Supp Figure** | **Sample** | **Number of tracks** | **Alpha (ɑ)** | **Diffusion coefficient (D, µm^2^/s x 10^-5^)** | **Gamma (Γ, µm^2^/s^ɑ^ x 10^-5^)** |
| **8** | SIM, no registration | 2186 | 1.16 | 19.20 | 8.80 |
|  | SIM, registration | 2113 | 0.95 | 8.32 | 10.72 |
|  | WF, no registration | 1032 | 1.21 | 16.90 | 6.09 |
|  | WF, registration | 1000 | 0.76 | 5.44 | 18.18 |
| **Supp Figure** | **Sample** | **average number of tracks per cell** | – | – | – |
| **9B** | fixed cell small foci | 237.9 | – | – | – |
|  | fixed cell very small foci | 790.0 | – | – | – |
|  | live cell small foci (37 °C) | 211.3 | – | – | – |
|  | live cell small foci (RT) | 256.8 | – | – | – |
| **Supp Figure** | **Sample** | **Number of tracks** | **Median** | **Mean** | **P value** |
| **11 - A** | Track straightness, SIM | 2213 | 0.3775 | 0.4264 | 0.61907 |
|  | Track straightness, WF | 1000 | 0.3620 | 0.4351 | 0.61907 |
| **Supp Figure** | **Sample** | **Number of tracks** | **Median (µm)** | **Mean (µm)** | **P value** |
| **11 - B** | Distance start-end, SIM | 2213 | 0.1580 | 0.1797 | 0.000000 |
|  | Distance start-end, WF | 1000 | 0.1372 | 0.1559 | 0.000000 |
| **Supp Figure** | **Sample** | **Number of tracks** | **Alpha (ɑ)** | **Diffusion coefficient (D, µm^2^/s x 10^-5^)** | **Gamma (Γ, µm^2^/s^ɑ^ x 10^-5^)** |
| **12** | G1/G2, SIM | 519 | 1.04 | 13.01 | 10.59 |
|  | S-Phase, SIM | 1594 | 0.92 | 07.05 | 10.45 |
|  | G1/G2, WF | 257 | 0.81 | 07.95 | 20.79 |
|  | S-Phase, WF | 743 | 0.78 | 04.95 | 14.83 |
| **12** | 37 ℃, SIM | 2113 | 0.95 | 8.32 | 10.72 |
|  | 37 ℃, WF | 1000 | 0.76 | 5.44 | 18.18 |
|  | RT, SIM | 2568 | 0.83 | 3.97 | 9.54 |
|  | RT, WF | 1104 | 0.71 | 2.73 | 11.88 |
| **Supp Figure** | **Sample** | **Number of tracks** | **Median (µm)** | **Mean (µm)** | **P value** |
| **13-A** | Radius of gyration, SIM  Population 0  Population 1 | 1308 805 | 0.0730 0.0820 | 0.0792 0.0861 | 0.0001165 |
|  | Radius of gyration, WF  Population 0  Population 1 | 619 381 | 0.0686 0.0733 | 0.0719 0.0771 | 0.059529 |
| **Supp Figure** | **Sample** | **Number of tracks** | **Median (µm/s)** | **Mean (µm/s)** | **P value** |
| **13-C** | Mean velocity, SIM  Population 0  Population 1 | 1308 805 | 0.006013 0.005543 | 0.006242 0.005716 | 0.000002 |
|  | Mean velocity, WF  Population 0  Population 1 | 619 381 | 0.005827 0.005169 | 0.006101 0.005561 | 0.000057 |
| **Supp Figure** | **Sample** | **Number of tracks** | **–** | **–** | **–** |
| **14** | Shell 1 (outer), SIM | 286 | – | – | – |
|  | Shell 2, SIM | 346 | – | – | – |
|  | Shell 3, SIM | 311 | – | – | – |
|  | Shell 4, SIM | 273 | – | – | – |
|  | Shell 5, SIM | 204 | – | – | – |
|  | Shell 6, SIM | 201 | – | – | – |
|  | Shell 7 (inner), SIM | 229 | – | – | – |
|  | Shell 1 (outer), WF | 154 | – | – | – |
|  | Shell 2, WF | 158 | – | – | – |
|  | Shell 3, WF | 134 | – | – | – |
|  | Shell 4, WF | 120 | – | – | – |
|  | Shell 5, WF | 96 | – | – | – |
|  | Shell 6, WF | 93 | – | – | – |
|  | Shell 7 (inner), WF | 110 | – | – | – |
| **Supp Figure** | **Sample** | **Sample** | **Median** | **Mean** | **P value (shell 1 to shell n)** |
| **14** | Alpha values, SIM | Shell 1 (outer) | 0.9401 | 1.0485 | NA |
|  |  | Shell 2 | 0.8447 | 0.9526 | 0.15615 |
|  |  | Shell 3 | 0.9003 | 0.9567 | 0.20455 |
|  |  | Shell 4 | 0.8836 | 0.9552 | 0.28661 |
|  |  | Shell 5 | 0.7950 | 0.9244 | 0.09317 |
|  |  | Shell 6 | 0.9521 | 1.0155 | 0.99791 |
|  |  | Shell 7 (inner) | 0.9590 | 1.0287 | 0.97765 |
|  | Alpha values, WF | Shell 1 (outer) | 0.9908 | 1.0662 | NA |
|  |  | Shell 2 | 0.6977 | 0.8406 | 0.00150 |
|  |  | Shell 3 | 0.8242 | 0.9578 | 0.17688 |
|  |  | Shell 4 | 0.7667 | 0.8583 | 0.00269 |
|  |  | Shell 5 | 0.7446 | 0.8729 | 0.0194 |
|  |  | Shell 6 | 0.7854 | 0.8634 | 0.04262 |
|  |  | Shell 7 (inner) | 0.7149 | 0.8829 | 0.027413 |
| **Supp Figure** | **Sample** | **Sample** | **Median (µm)** | **Mean (µm)** | **P value (shell 1 to shell n)** |
| **14** | Distance start-end, SIM | Shell 1 (outer) | 0.1473 | 0.1667 | NA |
|  |  | Shell 2 | 0.1501 | 0.1795 | 0.135905 |
|  |  | Shell 3 | 0.1667 | 0.1826 | 0.024090 |
|  |  | Shell 4 | 0.1615 | 0.1803 | 0.066405 |
|  |  | Shell 5 | 0.1369 | 0.1546 | 0.210906 |
|  |  | Shell 6 | 0.1641 | 0.1748 | 0.167986 |
|  |  | Shell 7 (inner) | 0.1507 | 0.1723 | 0.453838 |
|  | Distance start-end, WF | Shell 1 (outer) | 0.1273 | 0.1428 | NA |
|  |  | Shell 2 | 0.1357 | 0.1512 | 0.559429 |
|  |  | Shell 3 | 0.1416 | 0.1680 | 0.018751 |
|  |  | Shell 4 | 0.1292 | 0.1478 | 0.882723 |
|  |  | Shell 5 | 0.1385 | 0.1533 | 0.479737 |
|  |  | Shell 6 | 0.1257 | 0.1448 | 0.975072 |
|  |  | Shell 7 (inner) | 0.1273 | 0.1456 | 0.500560 |
| **Supp Figure** | **Sample** | **Sample** | **Median** | **Mean** | **P value (shell 1 to shell n)** |
| **14** | Track straightness, SIM | Shell 1 (outer) | 0.3730 | 0.4212 | NA |
|  |  | Shell 2 | 0.3731 | 0.4140 | 0.672754 |
|  |  | Shell 3 | 0.3783 | 0.4257 | 0.782575 |
|  |  | Shell 4 | 0.3608 | 0.4281 | 0.979520 |
|  |  | Shell 5 | 0.3434 | 0.4126 | 0.398322 |
|  |  | Shell 6 | 0.3741 | 0.4255 | 0.810307 |
|  |  | Shell 7 (inner) | 0.3778 | 0.4197 | 0.8707723 |
|  | Track straightness, WF | Shell 1 (outer) | 0.3947 | 0.4595 | NA |
|  |  | Shell 2 | 0.3268 | 0.4106 | 0.047195 |
|  |  | Shell 3 | 0.3672 | 0.4384 | 0.577206 |
|  |  | Shell 4 | 0.3420 | 0.4137 | 0.110704 |
|  |  | Shell 5 | 0.3290 | 0.4099 | 0.110695 |
|  |  | Shell 6 | 0.3856 | 0.4362 | 0.461081 |
|  |  | Shell 7 (inner) | 0.3188 | 0.4069 | 0.086930 |
| **Supp Figure** | **Sample** | **Number of tracks** | **–** | **–** | **–** |
| **14** | Shell 1 (outer), SIM | 1098 | – | – | – |
|  | Shell 2 (inner), SIM | 752 | – | – | – |
|  | Shell 1 (outer), WF | 504 | – | – | – |
|  | Shell 2 (inner), WF | 361 | – | – | – |
| **Supp Figure** | **Sample** | **Number of tracks** | **Median** | **Mean** | **P value** |
| **14** | Radius of gyration, SIM  Shell 1 (outer)  Shell 2 (inner) | 1098 752 | 0.07614 0.07132 | 0.08111 0.07680 | 0.0086527 |
|  | Radius of gyration, WF  Shell 1 (outer)  Shell 2 (inner) | 504 361 | 0.06833 0.06752 | 0.07269 0.07030 | 0.1709711 |
| **14** | Track straightness, SIM  Shell 1 (outer)  Shell 2 (inner) | 1098 752 | 0.3744 0.3638 | 0.4230 0.4180 | 0.484973 |
|  | Track straightness, WF  Shell 1 (outer)  Shell 2 (inner) | 504 361 | 0.3531 0.3380 | 0.4336 0.4159 | 0.226971 |
| **14** | Alpha values, SIM  Shell 1 (outer)  Shell 2 (inner) | 1098 752 | 0.8909 0.8959 | 0.9900 0.9695 | 0.7855995 |
|  | Alpha values, WF  Shell 1 (outer)  Shell 2 (inner) | 504 361 | 0.8268 0.7282 | 0.9589 0.8485 | 0.066803 |
| **14** | Mean velocity, SIM  Shell 1 (outer)  Shell 2 (inner) | 1098 752 | 0.005836 0.005518 | 0.006016 0.005809 | 0.029837 |
|  | Mean velocity, WF  Shell 1 (outer)  Shell 2 (inner) | 504 361 | 0.005440 0.005479 | 0.005730 0.005709 | 0.902479 |
| **14** | Distance start-end, SIM  Shell 1 (outer)  Shell 2 (inner) | 1098 752 | 0.1605 0.1463 | 0.1783 0.1677 | 0.023000 |
|  | Distance start-end, WF  Shell 1 (outer)  Shell 2 (inner) | 504 361 | 0.1365 0.1263 | 0.1538 0.1463 | 0.089760 |
| **Supp Figure** | **Sample** | **Number of tracks** | **–** | **–** | **–** |
| **14** | Shell 1 (outer), SIM | 167 | – | – | – |
|  | Shell 2, SIM | 259 | – | – | – |
|  | Shell 3, SIM | 252 | – | – | – |
|  | Shell 4, SIM | 215 | – | – | – |
|  | Shell 5, SIM | 205 | – | – | – |
|  | Shell 6, SIM | 170 | – | – | – |
|  | Shell 7, SIM | 139 | – | – | – |
|  | Shell 8, SIM | 145 | – | – | – |
|  | Shell 9, SIM | 143 | – | – | – |
|  | Shell 10 (inner), SIM | 155 | – | – | – |
|  | Shell 1 (outer), WF | 98 | – | – | – |
|  | Shell 2, WF | 111 | – | – | – |
|  | Shell 3, WF | 119 | – | – | – |
|  | Shell 4, WF | 96 | – | – | – |
|  | Shell 5, WF | 80 | – | – | – |
|  | Shell 6, WF | 83 | – | – | – |
|  | Shell 7, WF | 70 | – | – | – |
|  | Shell 8, WF | 63 | – | – | – |
|  | Shell 9, WF | 71 | – | – | – |
|  | Shell 10 (inner), WF | 74 | – | – | – |
| **Supp Figure** | **Sample** | **Sample** | **Median (µm)** | **Mean (µm)** | **P value (shell 1 to shell n)** |
| **14** | Radius of gyration, SIM | Shell 1 (outer) | 0.06506 | 0.07068 | NA |
|  |  | Shell 2 | 0.07227 | 0.07930 | 0.0216720 |
|  |  | Shell 3 | 0.08057 | 0.08493 | 0.0000507 |
|  |  | Shell 4 | 0.08073 | 0.08353 | 0.0001354 |
|  |  | Shell 5 | 0.08274 | 0.08464 | 0.0000772 |
|  |  | Shell 6 | 0.07102 | 0.07819 | 0.0656678 |
|  |  | Shell 7 | 0.06727 | 0.07231 | 0.6223969 |
|  |  | Shell 8 | 0.07690 | 0.08005 | 0.0065577 |
|  |  | Shell 9 | 0.07356 | 0.07749 | 0.0493245 |
|  |  | Shell 10 (inner) | 0.07005 | 0.07563 | 0.0187361 |
|  | Radius of gyration, WF | Shell 1 (outer) | 0.06233 | 0.06298 | NA |
|  |  | Shell 2 | 0.06646 | 0.07138 | 0.0577303 |
|  |  | Shell 3 | 0.07209 | 0.07753 | 0.0007889 |
|  |  | Shell 4 | 0.07171 | 0.07436 | 0.0022220 |
|  |  | Shell 5 | 0.07399 | 0.07721 | 0.0023389 |
|  |  | Shell 6 | 0.07024 | 0.07196 | 0.0184034 |
|  |  | Shell 7 | 0.07315 | 0.07132 | 0.0686121 |
|  |  | Shell 8 | 0.06422 | 0.07337 | 0.1207169 |
|  |  | Shell 9 | 0.07116 | 0.07283 | 0.0543791 |
|  |  | Shell 10 (inner) | 0.05993 | 0.06243 | 0.7736363 |
| **14** | Track straightness, SIM | Shell 1 (outer) | 0.3790 | 0.4264 | NA |
|  |  | Shell 2 | 0.3650 | 0.4130 | 0.436420 |
|  |  | Shell 3 | 0.3773 | 0.4223 | 0.800939 |
|  |  | Shell 4 | 0.3728 | 0.4158 | 0.704146 |
|  |  | Shell 5 | 0.3782 | 0.4412 | 0.7007078 |
|  |  | Shell 6 | 0.3670 | 0.4089 | 0.406041 |
|  |  | Shell 7 | 0.3300 | 0.4137 | 0.229260 |
|  |  | Shell 8 | 0.3815 | 0.4332 | 0.672914 |
|  |  | Shell 9 | 0.3494 | 0.4134 | 0.420675 |
|  |  | Shell 10 (inner) | 0.3816 | 0.4220 | 0.868215 |
|  | Track straightness, WF | Shell 1 (outer) | 0.3964 | 0.4582 | NA |
|  |  | Shell 2 | 0.3536 | 0.4360 | 0.290698 |
|  |  | Shell 3 | 0.3377 | 0.4124 | 0.086100 |
|  |  | Shell 4 | 0.3765 | 0.4369 | 0.420457 |
|  |  | Shell 5 | 0.3462 | 0.4277 | 0.416261 |
|  |  | Shell 6 | 0.3488 | 0.4287 | 0.241937 |
|  |  | Shell 7 | 0.3031 | 0.3933 | 0.059388 |
|  |  | Shell 8 | 0.3422 | 0.4315 | 0.450187 |
|  |  | Shell 9 | 0.3557 | 0.4132 | 0.167848 |
|  |  | Shell 10 (inner) | 0.3181 | 0.4124 | 0.151283 |
| **14** | Alpha values, SIM | Shell 1 (outer) | 0.9836 | 1.0834 | NA |
|  |  | Shell 2 | 0.8700 | 0.9604 | 0.0968973 |
|  |  | Shell 3 | 0.8447 | 0.9735 | 0.0786903 |
|  |  | Shell 4 | 0.8592 | 0.9586 | 0.0685896 |
|  |  | Shell 5 | 0.9716 | 1.0042 | 0.4269511 |
|  |  | Shell 6 | 0.8212 | 0.9010 | 0.0249519 |
|  |  | Shell 7 | 0.8105 | 0.8854 | 0.0154331 |
|  |  | Shell 8 | 0.9295 | 1.0226 | 0.5605983 |
|  |  | Shell 9 | 0.9683 | 1.0291 | 0.6559403 |
|  |  | Shell 10 (inner) | 0.9501 | 1.0153 | 0.4211240 |
|  | Alpha values, WF | Shell 1 (outer) | 1.1302 | 1.1148 | NA |
|  |  | Shell 2 | 0.8063 | 0.9704 | 0.0274637 |
|  |  | Shell 3 | 0.6748 | 0.8127 | 0.0005340 |
|  |  | Shell 4 | 0.7830 | 0.9051 | 0.0222278 |
|  |  | Shell 5 | 0.8788 | 1.0337 | 0.0689346 |
|  |  | Shell 6 | 0.7156 | 0.8222 | 0.0019695 |
|  |  | Shell 7 | 0.6909 | 0.8137 | 0.0032087 |
|  |  | Shell 8 | 0.7282 | 0.8369 | 0.0144681 |
|  |  | Shell 9 | 0.7986 | 0.8872 | 0.0259881 |
|  |  | Shell 10 (inner) | 0.6718 | 0.8834 | 0.0119245 |
| **14** | Mean velocity, SIM | Shell 1 (outer) | 0.004808 | 0.005092 | NA |
|  |  | Shell 2 | 0.005723 | 0.005855 | 0.0024424 |
|  |  | Shell 3 | 0.006069 | 0.006326 | 0.0000009 |
|  |  | Shell 4 | 0.006091 | 0.006318 | 0.0000008 |
|  |  | Shell 5 | 0.006019 | 0.006275 | 0.0000012 |
|  |  | Shell 6 | 0.005928 | 0.006086 | 0.000319 |
|  |  | Shell 7 | 0.005476 | 0.005736 | 0.067785 |
|  |  | Shell 8 | 0.005608 | 0.005874 | 0.007485 |
|  |  | Shell 9 | 0.005543 | 0.005789 | 0.0044535 |
|  |  | Shell 10 (inner) | 0.004838 | 0.005529 | 0.2769457 |
|  | Mean velocity, WF | Shell 1 (outer) | 0.004413 | 0.004804 | NA |
|  |  | Shell 2 | 0.005269 | 0.005651 | 0.002930 |
|  |  | Shell 3 | 0.005768 | 0.006202 | 0.000011 |
|  |  | Shell 4 | 0.005854 | 0.005911 | 0.000226 |
|  |  | Shell 5 | 0.005719 | 0.006057 | 0.000254 |
|  |  | Shell 6 | 0.005941 | 0.006149 | 0.0000491 |
|  |  | Shell 7 | 0.005598 | 0.005828 | 0.002264 |
|  |  | Shell 8 | 0.005292 | 0.005825 | 0.002496 |
|  |  | Shell 9 | 0.005442 | 0.005638 | 0.0028115 |
|  |  | Shell 10 (inner) | 0.004881 | 0.005071 | 0.376419 |
| **14** | Distance start-end, SIM | Shell 1 (outer) | 0.1390 | 0.1584 | NA |
|  |  | Shell 2 | 0.1487 | 0.1730 | 0.311918 |
|  |  | Shell 3 | 0.1649 | 0.1868 | 0.006579 |
|  |  | Shell 4 | 0.1667 | 0.1818 | 0.017904 |
|  |  | Shell 5 | 0.1733 | 0.1874 | 0.004527 |
|  |  | Shell 6 | 0.1419 | 0.1687 | 0.592970 |
|  |  | Shell 7 | 0.1298 | 0.1478 | 0.249994 |
|  |  | Shell 8 | 0.1733 | 0.1822 | 0.0141171 |
|  |  | Shell 9 | 0.1383 | 0.1690 | 0.3011126 |
|  |  | Shell 10 (inner) | 0.1461 | 0.1697 | 0.4572556 |
|  | Distance start-end, WF | Shell 1 (outer) | 0.1283 | 0.1436 | NA |
|  |  | Shell 2 | 0.1308 | 0.1470 | 0.901501 |
|  |  | Shell 3 | 0.1453 | 0.1575 | 0.373059 |
|  |  | Shell 4 | 0.1351 | 0.1610 | 0.292015 |
|  |  | Shell 5 | 0.1536 | 0.1614 | 0.141326 |
|  |  | Shell 6 | 0.1205 | 0.1480 | 0.925145 |
|  |  | Shell 7 | 0.1385 | 0.1469 | 0.907794 |
|  |  | Shell 8 | 0.1257 | 0.1513 | 0.835364 |
|  |  | Shell 9 | 0.1325 | 0.1540 | 0.858436 |
|  |  | Shell 10 (inner) | 0.1146 | 0.1322 | 0.154839 |
| **Supp Figure** | **Sample** | **Sample** | **Mbp** | **–** | **–** |
| **15** | **chromosome sizes** | 1 | 248.95642 | **–** | **–** |
|  |  | 2 | 242.19353 | **–** | **–** |
|  |  | 3 | 198.29556 | **–** | **–** |
|  |  | 4 | 190.21456 | **–** | **–** |
|  |  | 5 | 181.53826 | **–** | **–** |
|  |  | 6 | 170.80598 | **–** | **–** |
|  |  | 7 | 159.34597 | **–** | **–** |
|  |  | 8 | 145.13864 | **–** | **–** |
|  |  | 9 | 138.39472 | **–** | **–** |
|  |  | 10 | 133.79742 | **–** | **–** |
|  |  | 11 | 135.08662 | **–** | **–** |
|  |  | 12 | 133.27531 | **–** | **–** |
|  |  | 13 | 114.36433 | **–** | **–** |
|  |  | 14 | 107.04372 | **–** | **–** |
|  |  | 15 | 101.99119 | **–** | **–** |
|  |  | 16 | 90.338345 | **–** | **–** |
|  |  | 17 | 83.257441 | **–** | **–** |
|  |  | 18 | 80.373285 | **–** | **–** |
|  |  | 19 | 58.617616 | **–** | **–** |
|  |  | 20 | 64.444167 | **–** | **–** |
|  |  | 21 | 46.709983 | **–** | **–** |
|  |  | 22 | 50.818468 | **–** | **–** |
|  |  | X | 156.0409 | **–** | **–** |

**Video captions:**

**Video 1:** Simultaneous live cell imaging of GFP-PCNA (green) and labeled DNA (magenta) in Wide-Field (WF) and Structured Illumination Microscopy (SIM). Scale bar: 5 µm.

**Video 2:** HeLa K GFP-PCNA live cells labeled with Atto590-dUTP (magenta). Chromatin tracking over time of labeled DNA before and after registration. Scale bar: 5 µm.

**Video 3:** HeLa K GFP-PCNA live cells labeled with Atto590-dUTP (magenta). Chromatin tracking over time of labeled DNA at WF and SIM resolutions. Scale bar: 5 µm.

**Video 4:** Correlative chromatin tracking of labeled DNA at WF and SIM resolutions. Scale bar: 100 nm.

**Video 5:** HeLa K GFP-PCNA live cells labeled with Atto590-dUTP (magenta). G1G2 and S-phase cells at WF and SIM resolutions. Correlative chromatin tracking of labeled DNA at different resolutions and cell cycle stages. Scale bar: 5 µm.

**Video 6:** HeLa K GFP-PCNA live cells labeled with Atto590-dUTP (magenta) showing chromatin tracking at segregated chromosome territories, TAD domains (WF) and chromatin loops (SIM). Scale bar: 5µm.

**References**

[Chagin VO, Casas-Delucchi CS, Reinhart M, et al (2016) 4D Visualization of replication foci in mammalian cells corresponding to individual replicons. Nat Commun 7:11231. https://doi.org/10.1038/ncomms11231](https://sciwheel.com/work/bibliography/2069726)

[Erfle H, Neumann B, Liebel U, et al (2007) Reverse transfection on cell arrays for high content screening microscopy. Nat Protoc 2:392–399. https://doi.org/10.1038/nprot.2006.483](https://sciwheel.com/work/bibliography/1001802)
